# Computational prediction of human deep intronic variation

**DOI:** 10.1093/gigascience/giad085

**Published:** 2023-10-25

**Authors:** Pedro Barbosa, Rosina Savisaar, Maria Carmo-Fonseca, Alcides Fonseca

**Affiliations:** LASIGE, Departamento de Informática, Faculdade de Ciências, Universidade de Lisboa, 1749-016,, Lisboa, Portugal; Instituto de Medicina Molecular João Lobo Antunes, Faculdade de Medicina, Universidade de Lisboa, 1649-028, Lisboa, Portugal; Mondego Science, 3030-480, Coimbra, Portugal; Instituto de Medicina Molecular João Lobo Antunes, Faculdade de Medicina, Universidade de Lisboa, 1649-028, Lisboa, Portugal; LASIGE, Departamento de Informática, Faculdade de Ciências, Universidade de Lisboa, 1749-016,, Lisboa, Portugal

**Keywords:** abnormal splicing, introns, machine learning, model interpretability, variant prediction

## Abstract

**Background:**

The adoption of whole-genome sequencing in genetic screens has facilitated the detection of genetic variation in the intronic regions of genes, far from annotated splice sites. However, selecting an appropriate computational tool to discriminate functionally relevant genetic variants from those with no effect is challenging, particularly for deep intronic regions where independent benchmarks are scarce.

**Results:**

In this study, we have provided an overview of the computational methods available and the extent to which they can be used to analyze deep intronic variation. We leveraged diverse datasets to extensively evaluate tool performance across different intronic regions, distinguishing between variants that are expected to disrupt splicing through different molecular mechanisms. Notably, we compared the performance of SpliceAI, a widely used sequence-based deep learning model, with that of more recent methods that extend its original implementation. We observed considerable differences in tool performance depending on the region considered, with variants generating cryptic splice sites being better predicted than those that potentially affect splicing regulatory elements. Finally, we devised a novel quantitative assessment of tool interpretability and found that tools providing mechanistic explanations of their predictions are often correct with respect to the ground - information, but the use of these tools results in decreased predictive power when compared to black box methods.

**Conclusions:**

Our findings translate into practical recommendations for tool usage and provide a reference framework for applying prediction tools in deep intronic regions, enabling more informed decision-making by practitioners.

## Background

Genetic variation plays a crucial role in understanding human disease and trait inheritance. Yet, for a long time, studies paid scant attention to variants in intronic gene regions [1], which were thought to harbor little functional variation. With the advent of whole-genome sequencing (WGS) and the possibility to apply it at the population scale [2, 3], rare intronic variation can be identified at unprecedented levels. However, the sheer amount of candidate variants detected in the genome of an individual poses challenges for functional interpretation [4], particularly for variants affecting RNA splicing [5].

Splicing consists of removing introns from the primary transcript and is mediated by the spliceosome complex with the help of many RNA-binding proteins (RBPs) that recognize regulatory signals in exons and introns [6]. Splicing is tightly regulated across cell types and is sensitive to genetic variants occurring in *cis* (within the exons and introns of the splicing substrate) and in *trans* (within the genes encoding for splicing factors) [7]. It is estimated that 10% to 50% of all monogenic disease-causing variants affect pre–messenger RNA (mRNA) splicing [8–10]. In addition, cancer driver mutations are often associated with splicing alterations, notably in the case of *trans* variants that occur in genes encoding core components of the splicing machinery [11].

Of the *cis* variants that affect splicing, those that disrupt the splice sites (typically AG for the 3′ss and GT for the 5′ss) or the consensus region around the splice sites (nucleotides −12/+2 around the 3′ss and −3/+6 around the 5′’ss) have been studied the most thoroughly. Variants in these regions, especially if they affect the splice sites themselves, are fairly easy to recognize because the sequences are short and adhere to a highly conserved motif [12]. In contrast, other splicing variants can impact the binding of regulatory factors to splicing enhancers or silencers. These enhancers and silencers consist of short, poorly defined sequence motifs, which can occur at varying distances to the splice sites and can overlap either exons or introns [13]. It is thus difficult to identify them—and even more difficult to know when a mutation has disrupted them. Disruption of splicing information can lead to aberrant splice events such as exon skipping, full intron retention, or exon shortening or lengthening. Splicing variants can also create entirely new exons (“pseudoexons”). This can happen when a mutation creates a novel splice site, as well as when an existing but inactive (“cryptic”) splice site is activated by the creation of an enhancer motif or the disruption of a silencer motif [14].

There have been continuous efforts to systematically catalog disease-causing variation in databases such as ClinVar [15] or the Human Gene Mutation Database (HGMD) [16]. These resources show the enrichment of splicing-related variants in the vicinity of splice site regions. Partly, this reflects a biological reality, where the sequence around the splice sites is particularly dense in splicing-relevant information. However, this enrichment may also stem from the easier detection of splice site mutations, as well as biases in clinical guidelines for variant interpretation that may contribute to underestimating the significance of noncanonical splicing mutations, as there is a lack of standardized criteria for their interpretation [17, 18].

Therefore, it is expected that many splicing variants in other gene regions remain to be discovered. Our dearth of knowledge is greatest deep inside the introns, where the detection problem is the hardest given the large search space and the fact that the rare splice-affecting variants are greatly outnumbered by mutations with no effect. As a result, deep intronic variants often end up labeled as a variant of uncertain significance (VUS) [19], although a subset may have great clinical importance. Indeed, recent evidence has shown that deep intronic mutations triggering pseudoexon activation are an overlooked cause of human disease [20, 21].

Given the additional challenges of interpreting deep intronic mutations, computational tools are often used to prioritize variants based on their likelihood of being deleterious. The first wave of methods used large genomics datasets to engineer features (e.g., allele frequencies from ExaC [22] or histone modification levels across cell lines from ENCODE [23]) and to build classifiers that work on tabular data. More recently, end-to-end deep learning methods predict the impact of genetic variants from sequence alone, with the features automatically extracted within the network [24]. SpliceAI [10] is widely recognized as the most successful method of this kind, although its performance has been shown to vary across studies and datasets considered [5]. Recently, new models have been developed based on SpliceAI, either combining its predictions with other sources of information (such as genetic constraint for ConSpliceML [25] and PDIVAS [26] or tissue-specific splice site usage for AbSplice-DNA [27]) or creating an entirely new model based on SpliceAI architecture. For example, Pangolin [28] uses splicing quantifications from multiple species and tissues to not only predict whether a position is a splice site (as SpliceAI does) but also to predict splice site usage (e.g., how much a splice site is being used in a given tissue). In contrast, CI-SpliceAI [29] uses different training labels for true and false splice site positions based on a collapsed transcript structure derived from GENCODE [30] annotations.

Most intronic variant prediction benchmarking studies are performed by the authors of the tools to present a comparative analysis with existing methods. Even subconsciously, biases might be favoring the proposed model, be it because of the dataset selected or the methodology employed for the comparison [31, 32]. Multiple independent benchmark studies do exist [33–39], but their scope is often somewhat limited. First, some studies only focus on variants overlapping particular types of splicing information (e.g., splicing regulatory elements) [33, 35]. Second, only using variants from a small number of genes can render the genome-wide extrapolation of conclusions difficult [34, 36, 38]. Lastly, to our knowledge, no study compares the performance of promising and recently developed methods such as Pangolin, CI-SpliceAI, ConSpliceML, AbSplice-DNA, PDIVAS, and SPiP [40].

To help researchers and clinical practitioners understand prediction tools and how they can be applied to interpret genetic variants in introns, we conducted a comprehensive evaluation of a series of tools for the task of predicting functional variation in the intronic space far from canonical splice sites. To this end, we carefully selected intronic variants from multiple sources and curated a new set of disease-causing deep intronic variants affecting RNA splicing. Besides evaluating the capacity of tools to predict functional variants deep within the introns, we report, for the first time, an assessment of the interpretability of the output of these tools. We finally provide clear recommendations for tool usage depending on the variant’s location within the intron and its molecular effect.

## Results

### The prediction tools studied are diverse in methodology and objectives

In this study, we have provided a snapshot of the state-of-the-art of methods that predict, in any way, functional variation in introns (Table 1). We divided the methods into 4 different categories: conservation scores that measure the degree of evolutionary conservation at a given position or region of the genome, genome-wide predictors that integrate multiple feature types to predict variant effects regardless of the variant type, methods that focus on splice-disrupting variants and allow for automated batch predictions, and splicing-specific methods that solely target specific types of splicing information (e.g., Branchpoint [BP]) or require the use of a web application to retrieve results. For many tools, there are 2 fundamentally different ways to obtain predictions: making *de novo* model inferences given an input variant set or using precomputed predictions, which is faster computationally. We decided to use precomputed predictions when available because it considerably simplifies the variant annotation pipeline and is thus accessible to a more diverse set of end users. However, it should be noted that this approach may miss some indels that are not represented in the precomputed databases. Of the 38 tools used to score at least 1 dataset in this article, 19 had precomputed databases available (Table 1). Because some of them only provide predictions for the GRCh37 genome build, we ran all experiments using this genome version. Of note, precomputed predictions are a permanent representation of a model version, which may not be updated along with developments to the tool. However, we observed that only 1 tool, CAPICE [41], had outdated precomputed scores.

**Table 1:** Summary of the computational methods used in this study

Tool*	RRID	Threshold^†^	Description	Method	Training data	Predictions from^‡^	Used in analysis**
Conservation							
phastCons 100way [42]	—	>0.99 [43]	Probability that each nucleotide belongs to a conserved element	Hidden Markov model	Genomes of 100 vertebrates	Precomputed (UCSC)	ClinVar
phyloP 100way [44]	—	>1.6 [45]	*P* value that indicates how aligned sequences deviate from the null hypothesis of neutral evolution	Hidden Markov model	Genomes of 100 vertebrates	Precomputed (UCSC)	ClinVar
SiPhy 29way [46]	SCR_000564	>12.7 [45]	Identification of constrained sites as those with a nucleotide substitution pattern significantly deviating from the neutral pattern	Maximum likelihood and hidden Markov model	Genomes of 29 mammals	Precomputed (dbNSFP)	ClinVar
GERP [47]	SCR_000563	>4.4 [45]	Identification of evolutionarily constrained elements	Maximum likelihood to estimate the evolutionary rate and dynamic programming	Genomes of 34 mammals	Precomputed (UCSC)	ClinVar
Genome-wide predictors							
FATHMM-MKL [48]	—	>0.5 [49]	Prediction of functional consequences of coding and noncoding SNVs using genomic annotations from ENCODE and conservation scores	Support vector machine based on multiple kernel learning	3,063 disease-implicated SNVs from HGMD; 5,252 negative instances from 1000G project [50]	Precomputed (dbNSFP)	ClinVar
Eigen v1.1 [51]	—	>4.87 [52]	Unsupervised learning approach to leverage the functional importance of genetic variants across the whole genome	Linear combination of the components of the leading eigenvector determined from a rank 1 matrix estimated from 3 genome-wide annotation blocks	418,997 variants from 1000G project	Precomputed (dbNSFP)	ClinVar
ReMM v0.3.1 [53]	SCR_023095	>0.984	Classifier to predict the potential of an arbitrary position in the genome to cause a Mendelian disease	Random forest	453 disease-implicated variants by manual curation	Precomputed (tool webpage)	ClinVar
LINSIGHT [54]	—	>0.056 [52]	Prediction of noncoding nucleotide sites at which mutations are likely to have deleterious fitness consequences	INSIGHT and Online stochastic gradient descent	Genomes of 54 unrelated human individuals	Precomputed (dbNSFP)	ClinVar
CAPICE v1.0 [41]	—	>0.02	A consequence-agnostic method for pathogenicity prediction	XGBoost	Data from ClinVar, VKGL [55], and specific publication	Precomputed (Zenodo)	ClinVar
CADD-Splice v1.6 [56]	—	>15 [45]	Prediction of the deleterious effect a variant has on an individual’s fitness	Logistic regression	16,627,775 of both proxy-neutral and proxy-deleterious variants	Precomputed (tool webpage)	ClinVar; Splicing Pathogenic
Splicing							
MaxEntScan [57]	SCR_016707	|Δ*Entropy*| > 3	Prediction of RNA splice site signal based on the maximum entropy principle	Maximum entropy distribution	8,500 real 5′SS and 3′SS; 180,000 decoy 5′SS and 3′SS	VEP plugin [58]	ClinVar; AU; NSD
dbscSNV v1.1 [59]	—	>0.6	*In silico* prediction of splice-altering variants based on an ensemble of individual methods	AdaBoost and random forest	Splice-altering variants from HGMD, SpliceDisease [60], and DBASS [60] databases. Negative variants from 1000G Project	Precomputed (dbNSFP)	ClinVar; Splicing Pathogenic
SPANR/SPIDEX v1.0 [61]	—	$\vert \Delta {PSI\_zscore}\vert > 2$	Prediction of how much SNVs cause splicing misregulation by measuring differential exon inclusion events	Bayesian deep neural network	RNA-seq data in 10,700 exons across 16 tissues	Precomputed (tool webpage)	ClinVar; SplicingPathogenic; BP
HAL [62]	SCR_022581	|Δ*PSI*| > 0.05^§^	Variant effect prediction on different isoform usage from alternative splicing events (alternative 5′ss and exon skipping)	Linear model using hexamer motif frequencies	Massive parallel reporter assay (MPRA) containing 265,137 minigenes in a library of alternative 5′ splice donors	Kipoi (only 5′ss model)	ClinVar; NSD; DD
TrAP v3.0 [63]	—	>0.174	Prediction of the damage caused by SNVs at the transcript level by incorporation of splicing-engineered features	Random forest	75 pathogenic synonymous variants; 402 synonymous variants as benign	Precomputed (tool webpage)	All
S-CAP v1.0 [52]	—	Several thresholds^‖^	Splicing-specific pathogenicity score derived from variant, exon, and gene importance measurements	Gradient boosting tree	17,059 splicing-related pathogenic variants from HGMD and ClinVar and 6,760,450 splicing region benign variants from gnomAD	Precomputed (tool webpage)	ClinVar; SplicingPathogenic; BP
KipoiSplice4 v0.1 [64]	—	>0.5	Ensemble method that incorporates predictions from 4 splicing-related models (HAL, MaxEntScan5, MaxEntScan3, and LaBranchoR)	Logistic regression	10,715 splice region variants from ClinVar and 2,959 variants from the dbscSNV paper [59]	Kipoi	ClinVar; SplicingPathogenic; BP
SpliceAI v1.3 [10]	—	>0.2	Splice site prediction from primary sequence	Deep residual neural network	Primary transcript of 13,384 genes, accounting for 130,796 donor-acceptor pairs, plus novel splice junctions observed in the Genotype-Tissue Expression (GTEx) data [65]	Precomputed (tool webpage)	All
MMSplice v1.03 [66]	—	|Δ*logitPSI*| > 1	Modular approach to study functional effects of variants on splicing	Linear model that combines coefficient of 5 neural network modules	MPRA (Vex-seq) designed to evaluate the effect of 2,059 ExAC variants on exon skipping^#^	Kipoi	ClinVar; SplicingPathogenic; BP
SQUIRLS v2.0.1 [67]	—	>0.074 [29]	Prediction of the effect of variants on splicing providing interpretable outputs	Logistic regression model combining predictions from 2 random forest classifiers (donor and acceptor)	Cytoband-aware split of 73,203 benign variants from ClinVar and 8,314 deleterious variants from ClinVar and manual curation of variants from literature	Model inference	All
Pangolin v1.02 [28]	—	>0.2	Splice site prediction from primary sequence across multiple tissues	Deep residual neural network	Sequences and splice site quantifications from 4 species: human, rhesus macaque, rat, and mouse	Model inference	All
CI-SpliceAI v1.0 [29]	—	>0.190	Same as SpliceAI	Deep residual neural network	Sequences of 18,580 genes with splice sites (428,275) collapsed from GENCODE isoforms	Model inference	All
ConSpliceML v0.0.6 [25]	—	>0.5	Combination of SpliceAI and SQUIRLS predictions along with a metric of genetic constraint against deleterious splicing variation	Random forest	18,317 splicing-altering HGMD variants plus benign *de novo* variants collected from whole-genome sequencing studies and GTEx	Precomputed (tool webpage)	All
AbSplice-DNA v0.0.1 [27]	—	>0.01	Aberrant splicing prediction using MMSplice, SpliceAI, and tissue-specific annotations derived from GTEx	Generalized additive model	Splicing outliers detected from 946 GTEx individuals with paired RNA-seq and WGS data	Precomputed (Zenodo)	All
MLCsplice [68]	—	>0.5	Meta-predictor incorporating multiple splicing-related scores to predict region-specific variants	Hybrid model based on XGBoost, CGBoost, and LightGBM	Positive variants obtained from DBASS and HGMD database. Negative variants retrieved from gnomAD, ExAC, and dbSNP [69] with MAF $> 10\%$	Precomputed (tool webpage)	ClinVar; SplicingPathogenic; BP
SPiP v2.1 [40]	—	>0.452	Prioritization of splicing variants by running complementary bioinformatic tools that model different splicing elements	Random forest	Random 50% split of 4,416 curated splicing-altering variants and 95,000 control variants	Model inference	All
PDIVAS v1.0.0 [26]	—	>0.151	Pathogenic prediction of deep intronic variation combining SpliceAI (including raw scores), MaxEntScan, and ConSplice features	Random forest	374 pathogenic variants from HGMD and Keegan et al. [21]; 153,794 benign variants from the 1000G project	Model inference^‡‡^	SplicingPathogenic; AU; EL; NSD; DD
Splicing (region-specific or web-based)							
ESEfinder v3.0 [70]	SCR_007088	|Δ*score*| > 0.5**	Identification of exonic splicing enhancers from weight matrices of 4 SR proteins derived from SELEX experiments	Scoring motifs of each SR protein against a predefined threshold (inferred from high-scoring randomly chosen sequences from the initial SELEX library)	—	Webpage & Own code	EL
ESRseq [71]	SCR_022270	|Δ*score*| > 0.5 [34]	QUEPASA, a minigene assay that measured the impact of 6-mer motifs in RNA splicing	Statistical comparison of observed splicing strengths in sequences where the motif is present vs. absent	—	Own code	EL
HEXplorer [72]	SCR_022269	|Δ*score*| > 14 [34]	RESCUE-based approach to score elements that enhance or repress splice site usage	Average *z* score HZei (based on hexamer frequencies in exonic vs. intronic sequences) of all 6 hexamers overlapping with any given nucleotide	—	Webpage & Own code	EL
IntSplice2 v2.0 [73]	—	>0.5	Prediction of pathogenic intronic SNVs upstream of splicing acceptors	LightGBM	1,787 of each class located at −50 to −3 bp of splicing acceptors. Pathogenic variants from HGMD and ClinVar. Neutral variants from dbSNP	Precomputed (tool webpage)	SplicingPathogenic; BP; AU
SVM-BPFinder [74]	—	|*score*| > 0.136 [33]	Branchpoint prediction using sequence signals and additional polypyrimidine tract features	Support vector machine	Positive sequences: intronic 9-mers conserved across multiple species. Negative sequences: random intronic 9-mers. Both sets had T and A at positions 4 and 6	Model inference & Own code (as in [33])	BP
BPP [75]	—	|*score*| > 0.0006 [33]	Branchpoint prediction using sequence features extracted from conserved intronic regions of the human genome	Mixture model to predict branchpoint motif combined with octanucleotide frequencies in the PPT region	223,606 human introns longer than 300 bp	Model inference & Own code (as in [33])	BP
LaBranchoR [76]	—	|Δ*score*| > 0.1	Prediction of splicing branchpoint signals from raw sequence	Bi-LSTM neural network	Highly confident branchpoints that matched GENCODE-annotated 3′ss	Kipoi	BP
BPHunter v2[77]	—	>1^††^	Detection of intronic variants that disrupt the branchpoint sequence	Integration of gradient boosting tree, random forest, and logistic regression with the majority voting for the final prediction	198,256 branchpoint positions with flanking 13-bp and 1 million 13-bp random intronic and exonic positions	Webpage & Own code	BP
SpliceRover [78]	—	>0.5***	Splice site prediction from primary sequence	Convolutional neural network	Sequences of *Arabidopsis* and human surrounding canonical splice donors and acceptors	Webpage & Own code	AU; NSD; DD
DSSP [79]	—	>0.5***	Prediction of the impact of SNVs on splicing using a combination of deep learning and standard machine learning with handcrafted features	Stack generalization to combine a convolutional neural network with random forest, XGBoost, and linear regression models	170-bp sequences representing 4,964 variants (with corresponding wild-type sequences) from the MaPSy experiment [80]	Model inference & Own code	NSD
Spliceator v1.0 [81]	—	>0.5***	Splice site prediction for multispecies data	Convolutional neural network	Sequences from multiple species, from protists to human	Model inference & Own code	AU; NSD; DD

* We refer to the specific tool version used, although for several tools, we did not find a reference pointing to any version.

† Cutoff used to discriminate pathogenic/functional variants. If the original paper did not provide a reference threshold, it was extracted from elsewhere, with another reference assigned.

‡ “Own code” refers to our package [82].

** Analysis where tool was used. Where acronyms are seen, it refers to the region-specific splicing analysis: BP = branchpoint associated; NSA = new splice acceptor; NSD = new splice donor; AU = acceptor upstream; DD = donor downstream; EL = exonic-like.

§ HAL scores PSI for the sequence containing alternative 5′ss variants. Therefore, for this work, a change in PSI >0.05 was defined as the relevant threshold.

‖ S-CAP authors provide different reference thresholds depending on the location and context of the variant. 3intronic: 0.006, exonic: 0.009, 5intronic: 0.006, 5core_dominant: 0.034, 5core_recessive: 0.367, 5extended: 0.005, 3core_dominant: 0.033, 3core_recessive: 0.264.

# Several models were fitted in the MMSplice paper. In the table, the details of a single model are provided, the one that predicts *DeltalogitPSI* changes, as it was the primary goal defined by the authors.

*** When no reported threshold was found, we set 0.5 as the default value.

†† BPHunter threshold adjusted to 1 after discussing with the tool’s author. Annotated variants with 0 score are shifted to 1, and all unannotated variants are assigned a score of 0.

‡‡ PDIVAS does provide precomputed scores, but those only include pathogenic predictions. To get scores for variants that are not predicted as pathogenic, we need to perform raw model inferences.

Importantly, not every tool considered was built with deep intronic regions in mind. For example, some tools were explicitly trained only to score consensus splice site variants (e.g., MaxEntScan [57], dbscSNV [59]), while others only output predictions up to an approximately defined distance between the variant and the nearest splice site (e.g., 300 bp for SPIDEX [61] or 50 bp for MLCSplice [68]). In addition, we ran certain models (KipoiSplice4 [64], HAL [62], MMSplice [66]) using the Kipoi framework [64], which further restricts predictions to a tool-specific distance between the splice site and the variant. Therefore, we expected these methods to perform poorly on some comparisons simply because the fraction of missing predictions should increase when moving further into the intron. Still, we decided to include these tools in the study because many of the variants evaluated locate within the distance that we expected these tools to cover.

It should also be noted that the tools were built for different tasks. While some models were designed to distinguish between pathogenic and benign variants (e.g., S-CAP [52], KipoiSplice4), others predict variant effects on splicing outcome, which does not necessarily translate into disease (e.g., SPiP, MMSplice). The latter category comprises sequence-based deep learning models such as SpliceAI or Pangolin. While these packages accept genetic variants in VCF format as input, it is important to note that the models primarily operate on sequences. They predict the probability of a given sequence position functioning as a splice site. If the model is run twice, once with the reference and once with the mutated sequence, it is possible to assess splice site alterations caused by genetic variants with the so-called delta score (mutated – reference allele). This has been the major practical use of the tool so far. Using the same approach, we also included several sequence-based methods that predict splicing-related elements. These include SpliceRover [78], DSSP [79], and Spliceator [81] for splice site-associated variants; ESEfinder [70], ESRseq [71], and HEXplorer [72] for variants affecting splicing regulatory elements; and SVM-BPFinder [74] and BPP [75] for variants impacting the BP signal. Of note, we only employed these methods for the datasets deemed to be relevant given their original task.

### Intronic pathogenic variants located beyond 10 bp from the splice sites are poorly predicted

**Figure 1:**
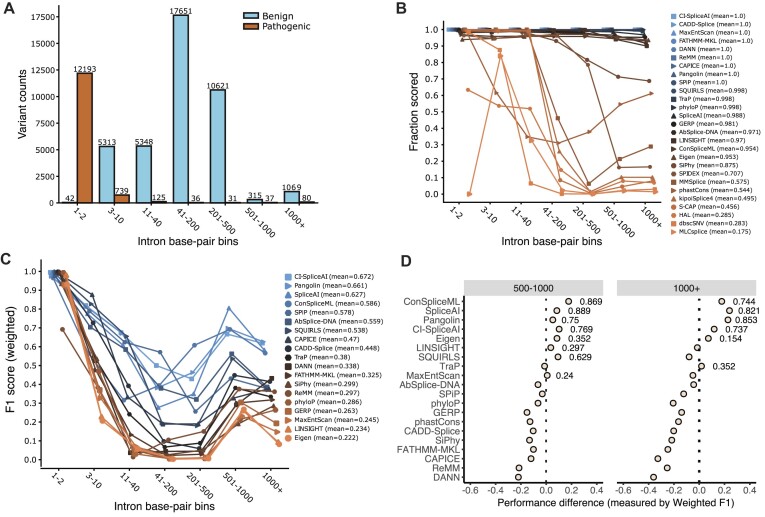
Intronic variant prediction in ClinVar. (A) Distribution of variants across each intronic bin considering the RefSeq transcript associated with each ClinVar variant. (B) Fraction of variants scored (with predictions) at each intronic bin. Mean values in the legend represent the average fraction of variants scored across all bins. (C) Performance of tools that predict entire introns (defined as >90% scored variants) at each intronic bin. Mean values in the legend represent the average weighted F1 score across all bins. (D) Differences in performance per deep intronic bins (“501–1000” and “1000+”) after removing variants that are exonic or closer to splice sites in other transcripts of the associated gene. Points refer to the weighted F1 difference between this new analysis minus the values obtained originally (displayed in C). Annotations next to points refer to the weighted F1 scores in the new analysis for the tools whose performance difference is positive.

We employed a bin-based analysis to evaluate ClinVar data (Supplementary Table S1). Because ClinVar contains disease-causing variants that act through different molecular mechanisms, we included not only splicing-related tools but also conservation scores and whole-genome predictors in the evaluations. Some of the models were trained using ClinVar data (Table 1), potentially leading to a circularity type I problem [83]. Fully correcting for this issue would have signified removing all ClinVar variants that were used in the training of any of the tools. This would have been problematic, as we would have lost many valuable deep intronic variants, which are typically scarce. However, most of the tools that were trained with ClinVar variants performed poorly. CAPICE was the only one to achieve a weighted F1 score above 0.6 across all bins (Supplementary Fig. S1A). We therefore only removed ClinVar variants that were used for training CAPICE (*N* = 14,189: 5,205 pathogenic and 8,984 benign). This is a trade-off, allowing for overestimated performance for some of the more underperforming tools while ensuring a sufficiently large dataset for the evaluation of all tools. After this filtering step, 53,600 variants remained for evaluation. As expected, the distribution of the 2 variant classes (pathogenic and benign) across bins is highly unbalanced (Fig. 1A). More than 90% of the intronic pathogenic variants occur at splice site positions, and more than 95% occur within 10 nucleotides from an exon–intron boundary.

Due to the spatial limitations discussed previously, we expected that some splicing tools would only output predictions for ClinVar variants located close to splice sites. Our results confirmed that several methods make predictions for less than 50% of the variants located at a distance of more than 40 bp from the nearest splice junction (Fig. 1B). The fraction of predicted variants decreases according to the expected regions that each model covers: 50 bp for S-CAP and MLCsplice and 300 bp for SPIDEX. MLCsplice was designed to predict noncanonical splicing variants (i.e., excluding splice site variants); thus, it is the only tool that displays no predictions at 1–2 positions (Fig. 1B). In addition, we observed that the tools run using the Kipoi framework (KipoiSplice4, HAL, MMSplice) displayed a notable drop 41 to 200 bp from the splice site. On the other hand, SQUIRLS [67], Pangolin, CI-SpliceAI, SPiP, TraP [63], SpliceAI, ConSpliceML, and AbSplice-DNA predicted across entire introns (Fig. 1B). Regarding the remaining tool categories, we observed that both whole-genome predictors and conservation scores (except phastCons [42]) output predictions for most ClinVar variants (Fig. 1B).

Next, we evaluated how the tools that score across full introns perform with ClinVar data. Performance dropped considerably for variants located deeper in intronic regions, especially once a distance of 10 nucleotides from the splice site had been reached (Fig. 1C). The splicing tools with the smallest and largest performance decrease between the splice site bin (“1–2”) and the “11–40” bin were Pangolin and TraP, with weighted F1 scores decreasing by 0.303 and 0.757, respectively (Supplementary Table S2, Fig. 1C). Conservation scores and whole-genome predictors performed poorly as well. Except for CAPICE and CADD-Splice, most methods displayed weighted F1 scores below 0.15 at the 11–40 bin (Supplementary Table S2). Overall, the most performant tools were CI-SpliceAI, Pangolin, and SpliceAI with average weighted F1 scores across all intronic bins of 0.672, 0.661, and 0.627, respectively (Fig. 1C).

Strikingly, we noticed an increase in performance in the deepest intronic bins when compared to intermediate distances (Fig. 1C). We hypothesized that variability in transcript structures could be the reason: despite these variants being assigned as occurring very deep within introns (>500 bp from the splice site) according to the associated RefSeq transcript, they may be exonic or near-splice site variants in other isoforms of the associated gene. To tackle this question, we looked at the raw transcript annotations (without picking Ensembl variant effect predictor [VEP] consequences) of the variants assigned to the >500-bp bin (*N* = 1,501) and decomposed them into several subcategories based on their localization in different transcript isoforms (see Methods). Our analysis revealed that 304 variants are located in exons in other transcripts and 274 variants mapped to introns but closer to splice sites than in the transcript isoform originally considered (Supplementary Fig. S1B). In particular, some of the intronic variants are located at splice sites in other transcripts (Supplementary Fig. S1C). We found that the performance of the tools was generally better for these categories than for categories where the variant distance to the splice site remained unchanged (Supplementary Fig. S1D), which is consistent with the hypothesis that deep intronic pathogenic variants are hard to predict. After excluding variants from exonic and closer-to-splice sites categories, we repeated the per-bin analysis to see whether the performance increase in the deepest bins remained. We observed that most conservation-based methods and whole-genome predictors displayed a decline in performance compared to the original analysis (Fig. 1D). On the other hand, a subset of splicing tools such as ConSplicemL, SpliceAI, Pangolin, or CI-SpliceAI showed better performance than before, suggesting that unequivocal deep intronic variants in ClinVar are associated with splicing and that SpliceAI-based methods can identify them reasonably well.

### Pathogenic splicing-affecting variants are captured well by deep learning–based methods

Not all ClinVar intronic variants are associated with splicing defects. However, splicing-related tools were the most successful at predicting the pathogenicity of deep intronic mutations. Therefore, we decided to narrow our focus to variants that specifically affected splicing. We previously published a dataset of deep intronic variants causing human disease via disruption of splicing (*N* = 81) [20]. In the current study, we augment the dataset by performing a comprehensive literature search for case reports published after 2017, where the association between a variant and a splicing defect was supported by experimental evidence, such as from reverse transcriptase polymerase chain reaction (RT-PCR), sequencing of complementary DNA (cDNA) products, RNA sequencing (RNA-seq), or minigene/midigene assays (Supplementary Table S3). This new curation effort is composed of 161 variants covering a diverse range of disease phenotypes, with most diseases represented by fewer than 3 variants (Supplementary Fig. S2A). A great number of these variants are not yet reported in ClinVar (*N* = 90), and of those that are reported, a few (*N* = 11) are incorrectly classified as VUS, with review status ranging from 0 to 1 stars (Supplementary Fig. S2B). As further evidence of their pathogenicity, the variants are very rare in the general population, as most of them are absent from gnomAD [2], a widely used catalog of genetic variation across human populations (Supplementary Fig. S2B, C).

**Figure 2:**
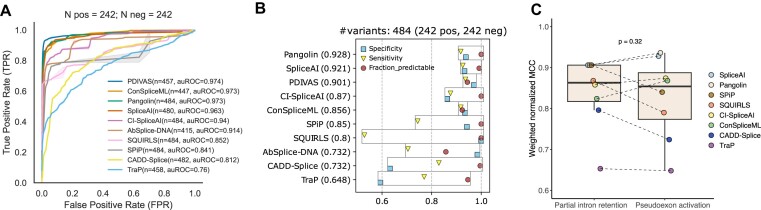
Pathogenic variant prediction of deep intronic variants affecting RNA splicing (81 variants from Vaz-Drago et al. [20] and 161 curated for this article). (A) Receiving operating characteristic (ROC) analysis for all splicing-associated methods. (B) Performance using optimized thresholds for intronic variation outside of canonical splice regions. The weighted normalized MCC was used to rank the tools. (C) Performance using optimized thresholds on 2 different subsets of variants: variants leading to partial intron retention and variants leading to pseudoexon activation. PDIVAS and AbSplice-DNA were excluded as they do not score the 2 groups equally (PDIVAS only predicts variants located 50 bp beyond the nearest splice site, while AbSplice-DNA scores variants within 100 bp of any splice junction observed in GTEx data). Wilcoxon signed rank test, performed as a 1-sided test, was used to compare the values between the 2 groups of variants.

The results showed that SpliceAI-derived methods outperformed the remaining tools. PDIVAS displayed the highest area under the receiver operating characteristic (auROC), followed by Pangolin, ConSpliceML, and SpliceAI (Fig. 2A). However, evaluation using single thresholds revealed lower performance than using auROC, which is based on multiple thresholds (Supplementary Fig. S2D). As practical clinical applications usually require a binary decision, this prompted us to optimize reference thresholds for detecting splice-affecting pathogenic intronic variation outside of the canonical splice site regions (see Methods). After threshold recalibration, we reveal SpliceAI and Pangolin as the best tools (weighted normalized Matthews correlation coefficient [MCC] >0.92) to identify pathogenic variants using a single cutoff value (Fig. 2B, Supplementary Table S4). As a practical outcome of this analysis, we provide recalibrated thresholds for different trade-offs between precision and recall (Supplementary Table S5).

When available, we recorded information on the molecular consequences of each variant on splicing. Pseudoexon activation was the most frequent consequence of deep intronic variants (194 out of 242 in our dataset). We also identified 37 variants leading to partial intron retention due to the usage of an alternative splice site. Exon skipping was observed in only 6 cases, consistent with previous observations that functional deep intronic variants are less commonly linked to this mechanism [84]. We next compared the tools’ ability to detect pseudoexon activation and partial intron retention variants using the optimized thresholds. We hypothesized that the tools would perform better on the partial intron retention group since these variants are located closer to the splice sites than those that activate pseudoexons (Supplementary Fig. S2E). Nonetheless, we observed no statistically significant differences between the 2 groups, with SpliceAI-derived methods performing slightly better in the pseudoexon activation group (Fig. 2C).

### Performance varies considerably when predicting splicing-altering variants associated with different molecular mechanisms

**Figure 3:**
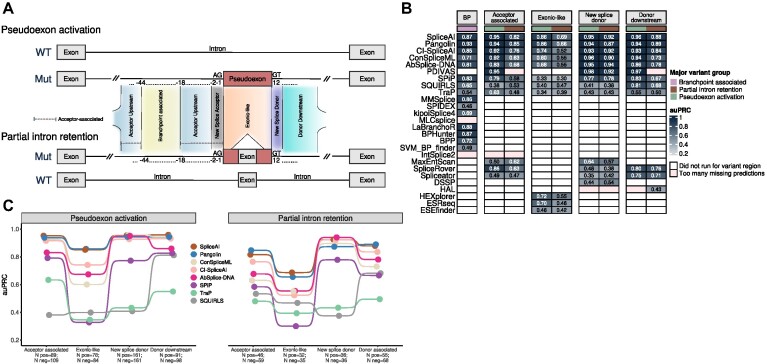
Tool performance evaluation on multiple regions associated with the regulation of splicing. (A) Schematic representation of the regions used to define each dataset. For each of the 2 major groups (pseudoexon activation and partial intron retention), we show the expected wild-type (WT) structure in the absence of the variant, as well as the abnormal structure caused by the variant (Mut). Red blocks represent regions of the mRNA that are incorrectly spliced in. Exceptionally, some branchpoint-associated variants result in exon skipping, which is not graphically represented in the figure. (B) Global overview of the performance (quantified with auPRC) for all the datasets analyzed. (C) auPRC scores for the tools that can predict entire introns.

To gain further insight into the molecular mechanisms driving the splicing alterations, we generated datasets of alternative splicing events triggered by intronic variants occurring at different regions that are important for splicing regulation (Table 2). We defined 6 categories (Fig. 3A, see Methods). Within each region, we separately evaluated variants that trigger partial intron retention (via alternative splice site usage at annotated exons) and variants that lead to pseudoexon activation. Importantly, contrary to the analyses performed above, we evaluate performance not based on the ability to distinguish between pathogenic and nonpathogenic variants but rather between variants that do (positive class) or do not (negative class) affect the mechanistic splicing outcome. This is relevant as a variant may, for example, create a pseudoexon, thus affecting the outcome of splicing, without necessarily leading to disease. This decision was made as information on variant pathogenicity was not always available. We also switch to reporting performance using the area under the precision–recall (PR) curve (auPRC), which is a metric to summarize precision–recall curve analysis. This decision was motivated by 2 factors. First, some categories have unbalanced data, with fewer positive instances compared to negatives. The auPRC score provides a more nuanced evaluation of tool performance by focusing on the accurate identification of positive instances. Furthermore, it eliminates the need for a single universal cutoff, accommodating the fact that different categories may have distinct optimal thresholds.

**Table 2:** Sources of data used to build region-specific splicing datasets

Study	Variants*	Per category**	Description
*Splicing-altering*			
Vaz-Drago et al. [20]	81	AU = 3; NSA = 12; EL = 10; NSD = 39; DD = 17	Manual curation of disease-causing deep intronic variants with experimental validations.
Our curation	140	BP = 7; AU = 11; NSA = 26; EL = 19; NSD = 43; DD = 34	Manual curation of disease-causing deep intronic variants with experimental validations.
Keegan et al. [21]	143	BP = 1; AU = 12; NSA = 21; EL = 13; NSD = 71; DD = 25	Characterization of hundreds of mutation events driving cryptic splicing via pseudoexon activation.
Petersen et al. [85]	10	BP = 1; AU = 1; EL = 7; DD = 1	Characterization of pseudoexons activated by deep intronic mutations that do not create or strengthen cryptic splice sites.
Tubeuf et al. [34]	3	EL = 3	Benchmark of user-friendly tools for predicting variants affecting splicing regulatory elements.
Jung et al. [84]	231	BP = 25; AU = 42; NSA = 3; EL = 56; NSD = 37; DD = 68	Identification of intronic missplicing mutations from RNA-seq using a read ratios approach.
Moles-Fernández et al. [35]	15	BP = 1; AU = 2; NSA = 2; EL = 2; NSD = 7; DD = 1	Benchmark of using both SpliceAI and user-friendly tools to identify deep intronic variants that disrupt splicing.
Leman et al. [33]	31	BP = 31	Benchmark of bioinformatics tools to predict BP as well as the impact of splicing variants occurring in the BP. area
Zhang et al. [77]	16	BP = 16	Genome-wide analysis of human branchpoints and development of a tool to score BP-associated variants.
*Splicing-neutral*			
Moles-Fernández et al. [35]	98	BP = 2; AU = 35; DD = 61	Benchmark of using both SpliceAI and user-friendly tools to identify deep intronic variants that disrupt splicing.
Vex-seq [86]	277***	BP = 59; AU = 52; EL = 119; DD = 47	Vex-seq, a MPRA to test the impact of 2,059 variants in splicing across 110 alternative exons.
MFASS [87]	109	BP = 34; AU = 17; DD = 58	Multiplexed functional assay (MFASS) that assayed the splicing effect of 27,733 ExAC variants.
gnomAD [2]	261	NSA = 64; NSD = 197	Common (and hypothetically benign) variants that create true splice site motifs.

* Total number of variants used from original study. Since several variants were duplicated across studies, we kept unique occurrences given the order they appear in the table (top to bottom).

** Number of variants contributing to each category. BP = branchpoint associated; NSA = new splice acceptor; NSD = new splice donor; AU = acceptor upstream; DD = donor downstream; EL = exonic-like. Note: we could not assign a category to all variants of our curation, hence the lower number as compared to the original dataset (*N* = 161).

*** Exceptionally, 119 variants from this study are exonic.

#### Branchpoint-associated variants

*Branchpoint-associated* variants were defined as located 18 to 44 bp upstream of the splice acceptor of a cryptic or canonical splice site, either leading to pseudoexon activation, partial/full intron retention, or exon skipping (Supplementary Table S6). In addition, we confirmed that the variant either disrupted or created any of the 4 (increasingly relaxed) BP motifs described in [77]: YTNAY, YTNA, TNA, YNA. Particularly, we excluded any splicing-altering variants located 1 bp upstream of the branchpoint adenine. The final positive branchpoint-associated dataset (*N* = 82) spans 7 different sources, with Leman et al. [33] contributing the most (*N* = 31, Table 2). The negative variants are located 18 to 44 bp upstream of an annotated splice site and had been shown not to affect splicing using the minigene-based reporter assays Vex-seq [86] and MFASS [87] (Table 2, Supplementary Table S6). Because BP variants activating pseudoexons were scarce (*N* = 4) and the molecular consequences of BP-associated variants are not clear-cut (e.g., the same BP variant may lead to intron retention and to exon skipping), we analyzed all the variants affecting the BP motif together. For this analysis, we additionally included 4 branchpoint prediction tools: SVM-BPFinder, BPP, LaBranchoR [76], and BPHunter [77]. Moreover, we included IntSplice2 [73] since it predicts splicing-associated single-nucleotide variants (SNVs) at intronic positions overlapping the branchpoint region.

Pangolin was the best-performing method for BP-associated variant prediction with an impressive auPRC score of 0.93 (Fig. 3B, Supplementary Fig. S3A). This result suggests that the training of Pangolin on multispecies data potentially contributed to increased robustness in capturing the complexity of the branchpoint code. Among the tools specifically designed to predict BPs, LabRanchoR, and BPHunter were very competent, ranking second and fourth, respectively, with auPRC scores of 0.877 and 0.87 (Supplementary Table S7). Conversely, BPP and SVM-BPFinder displayed more modest results.

#### Acceptor upstream and new splice acceptor variants

The *acceptor upstream* category refers to splicing-altering variants that mostly locate upstream (up to 18 bp) of an existing cryptic splice acceptor and activate it. On the other hand, the *new splice acceptor* category contains variants that form new splice sites themselves (Fig. 3A). We collected negative variants differently for each of the 2 categories. For *acceptor upstream* variants, we extracted variants located upstream of annotated splicing acceptors that did not interfere with the splicing outcome, as demonstrated through MFASS, Vex-seq, or Moles-Fernández et al. [35]. The BP region from 18 to 44 bp was excluded. Conversely, we assigned common (>5% allele frequency) deep intronic gnomAD variants that create new splice acceptor motifs as negative *new splice acceptor* variants (Table 2, see also Methods). Despite creating a splice acceptor motif, these variants are considered nonfunctional due to their high prevalence in the general population. While it is theoretically possible that these variants do affect splicing (e.g, if they occur in nonessential genes where splicing alterations have little fitness effect), we confirmed that their genomic locations were not used as splice junctions in individuals from the GTEx [65] cohort.

The sets of splicing-altering variants we collected for each category were similar in size (71 and 64 variants for acceptor-upstream and new splice acceptor, respectively). However, when we split the variants according to the major molecular group (pseudoexon inclusion vs. partial intron retention), we obtained a very small number of new splice acceptor variants in the partial intron retention group (*N* = 13), hence rendering their computational evaluation statistically limited. Therefore, for this particular analysis, we merged *acceptor upstream* and *new splice acceptor* variants into a new *acceptor-associated* class so that we could have a reasonably large dataset to evaluate (Supplementary Table S6). As for the *branchpoint-associated* variants, we added IntSplice2 to the list of tools to evaluate. In addition, we included 2 splice site prediction methods that we customized to predict variant effects in VCF format: SpliceRover and Spliceator.

SpliceAI, PDIVAS, Pangolin, ConSpliceML, and CI-SpliceAI achieved good performance on pseudoexon-activating variants, with auPRC above 0.9 (Fig. 3B). However, when it comes to variants causing partial intron retention, performance drops considerably, with no tool achieving an auPRC score higher than 0.85 (Fig. 3B). Except for PDIVAS, which had a substantial amount of missing data for this analysis, the top tools remained unchanged, with Pangolin, SpliceAI, and CI-SpliceAI displaying AP scores of 0.847, 0.816, and 0.765, respectively (Supplementary Fig. S3C, Supplementary Table S7). Among the tools specifically added for this analysis, SpliceRover was the most competitive, ranking sixth in the pseudoexon group and fifth for partial intron retention variants (Supplementary Fig. S3B, C).

#### Exonic-like variants

We consider here intronic variants that lie within either an activated pseudoexon or an annotated exon that undergoes alternative splice site usage (Fig. 3A). We identified 110 splicing-altering variants to compare against 119 splicing-neutral exonic variants from Vex-seq (Supplementary Table S6). After grouping the variants according to the major group, we obtained 78 pseudoexon-activating variants vs. 32 variants triggering partial intron retention. Accordingly, we randomly split the negative the variants between the 2 groups so that the final datasets were fairly balanced (84 and 35 variants for each group, respectively). For this comparison, we also included 3 approaches that quantify splicing regulatory elements that enhance or repress flanking splice sites: ESREseq scores, HEXplorer, and ESEfinder.

Once again, we observed better overall performance for the pseudoexon group compared to the partial intron retention group (Fig. 3B, Supplementary Fig. S3D, E). Pangolin and SpliceAI were among the best tools in both major groups. Interestingly, HEXplorer and ESREseq performed better for the pseudoexon group than models that incorporate deep learning–based predictions such as AbSplice-DNA or ConSpliceML (Supplementary Fig. S3D, Supplementary Table S7).

Although SpliceAI performed best compared to other methods, its precomputed scores were configured to only report variant effects in a 50-bp window from the variant site. While this window is fine for most variant types (the affected splice sites are usually close to the variant site), that may not be the case for pseudoexon-activating variants that could be located deep inside the pseudoexon (assuming a pseudoexon of the size of an annotated exon). Therefore, we selected the splicing-altering variants missed by SpliceAI using the optimized threshold of 0.05 (*N* = 25) and used the SpliceAI Lookup API [88] (last accessed 25 May 2023), to run the model using a larger maximum distance (500 bp). We observed that 9 out of 25 were correctly reclassified as splicing-altering (Supplementary Table S8), suggesting that SpliceAI performance may be underestimated when ignoring longer-range variant effects.

#### New splice donor variants

We identified 197 positive variants falling into this category (Table 2). For the negative set, we used variants that created a GT dinucleotide, resulting in a splice donor consensus (GGTAAG), but that were unlikely to act as a cryptic splice site as they appeared in gnomAD with a population frequency >5% and were not observed to be used as a splice junction in GTEx individuals. We added SpliceRover, DSSP, and Spliceator tools to the evaluation.

PDIVAS demonstrated the best performance in the pseudoexon activation group, achieving an auPRC score of 0.981. On the other hand, AbSplice-DNA outperformed other tools for partial intron retention variants with a performance metric of 0.94 (Supplementary Fig. S3F, G). Similarly, SpliceAI, ConSpliceML, Pangolin, and CI-SpliceAI exhibited excellent results (Fig. 3B), indicating that these models are very well suited for predicting this category of variants. Importantly, we noticed a large performance gap between SpliceAI-related tools (plus SPiP) and the rest, which performed rather poorly (almost all tools with AP scores below 0.6, Fig. 3B, Supplementary Fig. S3F, G). Considering that splicing-negative variants in this dataset create hypothetical splice donor decoys, we wondered whether tools that incorporate cryptic splice site scoring features using short sequence windows surrounding the variant site (position-specific scoring matrix [PSSM] based for TraP, information content based for SQUIRLS) would predict negative variants as splicing-altering. Indeed, we observed a large proportion of false positives for these tools in the pseudoexon-activation group when using a single reference threshold for evaluation (1.0 for TraP and 0.98 for SQUIRLS, Supplementary Table S7). Conversely, deep learning–based methods such as SpliceRover, DSSP, and Spliceator may rely too much on the near-splice site features (despite using larger sequence contexts), hence the poor performance observed.

#### Donor downstream variants

This category refers to all splicing-altering intronic variants located downstream of the cryptic splice donor event (*N* = 146). Negative variants (*N* = 166) were located downstream of annotated exons and were shown experimentally to have no impact on splicing outcomes (Table 2, Supplementary Table S6). As before, we included SpliceRover and Spliceator in the analysis. DSSP was excluded since it predicts splice sites at fixed positions in the input, but in this category, variant positions with respect to the cryptic splice donor are variable.

PDIVAS and SpliceAI excelled on the subset of variants triggering pseudoexon activation with auPRC scores of 0.969 and 0.959, followed by ConSpliceML, Pangolin, and CI-SpliceAI, all with performance values above 0.9 (Fig. 3B, Supplementary Fig. S3H, Supplementary Table S7). Regarding the partial intron retention subset, Pangolin and SpliceAI performed the best (auPRC scores of 0.89 and 0.879), with a larger difference for the tool ranked third, CI-SpliceAI (AP = 0.836, Supplementary Fig. S3I). Again, these results demonstrate the superiority of SpliceAI-derived approaches versus standard methods that engineer domain-specific features to score intronic splicing variation.

#### All regions combined

Next, we combined all the datasets to inspect the global performance of each major variant group. Eight methods were able to score all types of splicing variants in any intronic region. These tools were SpliceAI, Pangolin, ConSpliceML, CI-SpliceAI, AbSplice-DNA, SPiP, TraP, and SQUIRLS (Fig. 3C). Except for AbSplice-DNA, which scores intronic variants located up to 100 bp away from splice junctions used in any GTEx tissue, all the methods were designed to score any given position in introns.

SpliceAI and Pangolin consistently ranked highly for all datasets (Fig. 3C). CI-SpliceAI, AbSplice-DNA, and ConSpliceML were fair alternatives, especially for variants that create new splice donors. SPiP was particularly inadequate for exonic-like variants but was the best non–deep learning–based method for the remaining categories (Fig. 3C).

Overall, we observed a trend of pseudoexon-activating variants being predicted more accurately than partial intron retention variants (Fig. 3C, Supplementary Fig. S4A). However, when evaluating each tool individually, this trend did not reach statistical significance for the majority of them (Supplementary Fig. S4B).

### Assessing interpretability

We were interested in the extent to which these state-of-the-art tools give additional information to the user, besides the prediction. Among the tools that predict across whole introns, SQUIRLS and SPiP are the only ones intentionally designed to provide some interpretation of the outcome. SQUIRLS can generate HTML reports with short descriptions of why the model predicts pathogenicity and displays the contribution of each feature to the outcome. In addition, it draws figures to show the variant effect in the sequence context surrounding the variant. SPiP provides short interpretation tags describing the molecular consequences of the variants along with confidence intervals for the probability that the variant impacts splicing. Recently, a novel strategy was introduced to aid in the interpretation of splicing-associated variants, leveraging RNA-seq data from more than 300,000 individuals [89]. This approach, SpliceVault, focuses on quantifying the relative prevalence of stochastic and unannotated splicing events in population-based RNA-seq data, enabling the prediction of the nature of missplicing induced by a variant. Given its innovative approach and the ability to provide interpretations for variant consequences, we included SpliceVault in our assessment.

**Figure 4:**
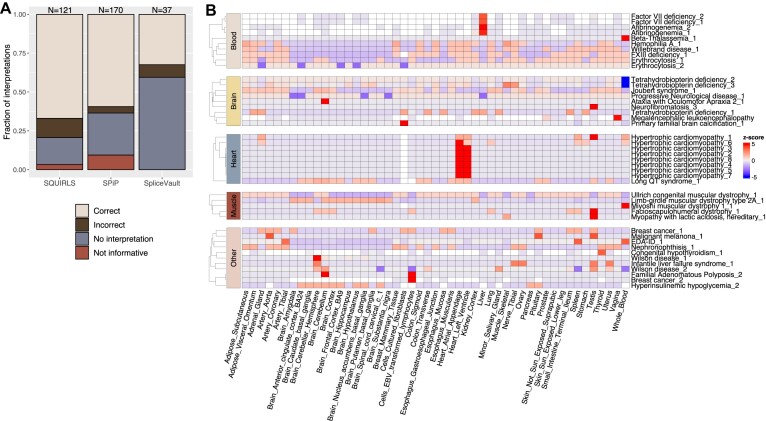
Information provided by the tools beyond the prediction score. (A) Assessing the quality of the interpretations for SQUIRLS, SPiP, and SpliceVault. Within each bar, the height of each category represents the fraction of variants assigned to the given interpretation quality tag. The numbers above the bars indicate how many pathogenic variants were used. (B) Tissue-specific predictions made by AbSplice-DNA for a set of disease-causing variants associated with a single tissue, according to Human Phenotype Ontology (HPO). Phenotype names are displayed in rows, GTEx tissues predicted by AbSplice-DNA are in columns. High *z* scores represent tissues for which the variant effect is stronger compared to other tissues.

We devised a procedure to evaluate how accurate the interpretations are against the biological ground truth (see Methods). We used the splicing-associated deep intronic pathogenic dataset analyzed before (Fig. 2) and specifically selected variants with complete annotations, including molecular effect and functional consequence (*N* = 221) for assessing interpretation quality. SPiP and SQUIRLS correctly predicted 170 and 121 variants, respectively, and those were selected for downstream analysis. In contrast, SpliceVault does not predict variant effects directly. Instead, it checks the missplicing occurring in the surroundings of an annotated exon of interest. As a result, we did not include pseudoexon-activating variants because SpliceVault cannot provide information about such an outcome (despite potentially identifying 1 of the 2 splice junctions of the pseudoexon). This left us with 37 variants for analysis (Supplementary Table S9). Our evaluation revealed that SQUIRLS, SPiP, and SpliceVault were able to provide correct interpretations (within the limitations of each approach) for a considerable fraction of the variants. However, for many others, no interpretation could be found. Specifically, SPiP lacked interpretations for 46 variants, SpliceVault for 22 variants, and SQUIRLS for 21 variants (Fig. 4A). In the case of SpliceVault, this accounted for more than half of the analyzed variants (22 out of 37). Further inspection of these results showed that most of these variants create a core splice site dinucleotide (Supplementary Fig. S5A), and this type of missplicing event is not appropriate to be captured by SpliceVault. Regarding SQUIRLS and SPiP, and looking at the prediction score distribution for each category, we observed that the variants with no interpretation have the lowest scores (Supplementary Fig. S5B). On the other hand, correct explanations are spread across the full score range. Interestingly, SPiP explanations that are not informative (events with no association with any splicing mechanism) have the highest median score range, showing that strong effects are not necessarily easier to explain.

### Predicting splicing changes across tissues

Of the tools evaluated in this study, Pangolin and AbSplice-DNA can both predict splicing outcomes in a tissue-specific fashion. We decided to use AbSplice-DNA alone for this analysis. Pangolin was trained on sequences and splice site usage levels from 4 tissues across 4 species (human, rhesus macaque, mouse, and rat). However, the default settings of Pangolin are tissue agnostic, and it requires additional customizations to get tissue-specific variant effect predictions. On the other hand, AbSplice-DNA provides precomputed tissue-specific predictions. Moreover, it combines tissue-specific splicing annotations created from GTEx data with DNA-based prediction models, enabling it to predict variant effects in more tissues (49). We aimed to evaluate whether AbSplice-DNA predictions of disease-causing variants are enriched for the tissues that are most strongly affected by the disease.

Using the splicing-associated variant dataset described above (*N* = 242, Fig. 2), we determined, when possible, the GTEx tissue most closely associated with the given disease based on the HPO [90] (see Methods). We selected the 155 variants that AbSplice-DNA predicted correctly, which excluded all the variants causing 2 of the most common diseases in our dataset: Becker muscular dystrophy and Duchenne muscular dystrophy (Supplementary Table S10). In addition, 35 variants were not evaluated since they were not assigned to any particular tissue (e.g., systemic diseases, or diseases affecting tissues not represented in GTEx, such as the retina), leaving 120 variants to analyze. Considering disease variants associated with only 1 GTEx tissue, we observed enrichment of the expected tissues to a limited extent (Fig. 4B, Supplementary Fig. S6A). For example, hypertrophic cardiomyopathy variants were highly enriched in the heart tissue, an ataxia with oculimotor apraxia variant was predicted to affect the cerebellum, and a congenital hypothyroidism variant was enriched for thyroid. Interestingly, variants associated with blood disorders (factor VII deficiency and afibrinogenemia) have the highest prediction scores in the liver, which is not unexpected, since the liver plays a crucial role in the production of clotting factors, including factor VII and fibrinogen (Fig. 4B). However, other tissue-specific predictions had unclear interpretations, such as the enrichment of testis for several diseases, the brain cerebellum in adenomatous polyposis (associated with colon and rectum, Fig. 4B), or the skeletal muscle in Fabry disease (primarily linked to other tissues such as heart and kidneys, Supplementary Fig. S6A). In addition, 40 variants displayed the same score across all tissues, which does not reflect the expected biology, especially for some diseases associated with a single tissue (Supplementary Fig. S6B).

## Discussion

We have performed a comprehensive benchmark study of intronic variant prediction, focusing on disease-causing deep intronic variants affecting splicing via pseudoexon inclusion or partial intron retention. Furthermore, we collected and examined variant sets based on their location relative to the splice sites affected by the altered splicing. Finally, we assessed tool interpretability and provide some considerations on the use of computational models beyond the prediction score.

We used 2 different datasets to study intronic variants causing human disease. ClinVar is a database that has been widely used for this purpose. Nevertheless, to the best of our knowledge, it has not been used to evaluate performance as a function of distance to the splice sites. Averaging performance across all bins, we found that splicing-associated tools performed the best overall on ClinVar data. Importantly, we observed a decrease in performance immediately after the 2 splice site positions, with a particularly noticeable decline at a distance of 11 base pairs from the closest splice site. These results demonstrate the extent to which these methods are biased to predict splice site variants, whereas smaller effect size variants deeper inside the intron go mostly unnoticed. For many of the tools, such as S-CAP or MLCsplice, this is not unexpected, as they were not designed to predict variants in deep intronic regions. In addition, we observed that some of the variants that appear deep-intronic in the clinically relevant transcript are exonic or located close to the splice sites in other isoforms of the associated gene. Therefore, and according to the American College of Medical Genetics and Genomics and the Association for Molecular Pathology guidelines [91], we recommend considering multiple isoforms when interpreting deep intronic variants, especially when the canonical isoform is not highly expressed in the tissue of interest [92].

Additionally, we curated a diverse set of pathogenic deep intronic mutations that exclusively affect splicing. Tools that predict across all intronic regions, notably SpliceAI-derived models, showed satisfactory performance. Many variants in this dataset generate new splice sites deep within introns, activating pseudoexons. We speculate that sequence-based models that predict splice sites are particularly well suited to predicting this class of variants, likely because the pseudoexons resemble the sequence context of authentic exons [85] that were presented during their training.

To better understand performance differences between classes of variants, we collected a diverse set of experimentally tested splicing-associated variants and evaluated the tools’ ability to distinguish them from similar non-splice-altering variants. Region-specific analysis revealed substantial differences in performance. In agreement with previous studies [35, 93], we found that variants affecting putative exonic splicing regulatory elements were among the hardest to predict. The binding motifs of many splicing factors are highly degenerate or even unknown, and their impact on splicing largely depends on the cell type [94, 95]. Nevertheless, such complexity appears to be better captured by SpliceAI and Pangolin than by tools with built-in domain knowledge.

The recent progress achieved through deep learning models that work as black boxes has raised concerns about their deployment in sensitive domains such as health care [96]. Because practitioners are interested in understanding how these AI systems make decisions, we assessed the capacity of these models to provide interpretable outputs when predicting disease-causing variation associated with splicing defects. Although most sequence-based models, such as SpliceAI, provide some information beyond the prediction score—namely, the distance of the variant to the affected mRNA position—it is only possible to obtain insight into the inner workings of the model by applying external explainability techniques [97]. On the other hand, SQUIRLS and SPiP are intrinsically more interpretable by design. The models were frequently able to correctly identify the type of splicing alteration. However, these models suffer from an accuracy–interpretability trade-off since the performance across evaluations was lower than that of black box models. The recently published SpiceVault portal also provides an accurate interpretation of the nature of missplicing defects, but it does have limitations that are particularly pronounced when dealing with variants deep in the introns. Particularly, it cannot properly analyze pseudoexon activation events or cryptic splicing caused by variants that create new splice sites at the core dinucleotide motif. Note that to our knowledge, no tool exists that can provide higher-order mechanistic interpretations, such as identifying the particular splicing factors or regulatory motifs involved.

Another promising research avenue is the prediction of splicing abnormalities in a tissue of interest, which AbSplice-DNA offers. The model could accurately detect some tissue-specific differences relevant to human disease, yet it was unreliable for the majority of variants. Nonetheless, we acknowledge that the introduction of SpliceMaps [27], which provides information on splice site usage across GTEx tissues, combined with RNA sequencing of clinically accessible tissues (CATs), is expected to enhance the prediction of functional intronic variants [27], particularly in diseases where the splicing landscape of the relevant nonaccessible tissue is appropriately represented by one of the CATs [98].

### Practical recommendations

We advocate using deep learning–based solutions to obtain maximally accurate predictions. SpliceAI and Pangolin consistently ranked high for intronic variants associated with splicing, for the prediction of both pathogenicity and altered splicing. We determined optimal thresholds for deep intronic regions (SpliceAI = 0.05, Pangolin = 0.053) for clinical purposes. However, it is important to note that despite the diversity of genes, phenotypes, and molecular mechanisms covered in our dataset, users should be mindful that optimal thresholds can vary depending on the variant class or affected exon [99].

SpliceAI and Pangolin are usually run programmatically on the command line. However, if usability is a primary concern and users have a limited number of predictions to make, the Broad Institute offers a convenient web application. The web application [88] incorporates both SpliceAI and Pangolin. For SpliceAI, it not only provides the conventional delta score (mutated – reference) but also presents the raw splice site probability predicted by the model. This can be particularly useful for certain situations. For instance, when a splice site is already predicted with a high score in the reference sequence (e.g., 0.85), the delta score for a splice-promoting mutation can only be low (no more than 0.15, in this example). This is because SpliceAI scores are capped at 1. This context is important for the correct interpretation of the delta scores. With this in mind, it is also worth considering SpliceAI-visual [100], available at [101]. SpliceAI-visual handles complex variant types and employs raw SpliceAI scores to generate graphical outputs that are easier to interpret. If the number of variants makes it unfeasible to use these web applications, but the user does not have the computational know-how to work on the command line, CI-SpliceAI is a good alternative, since its online service [102] allows the input of multiple variants in a VCF-like format. Practitioners, however, may suspect that splicing is not the mechanism disrupted by a particular mutation. In this scenario, we recommend using CAPICE since it was the best whole-genome predictor on ClinVar data, although with very limited performance.

Region-specific splicing benchmarks revealed additional insights for tool usage. We recommend using Pangolin to prioritize variants in branchpoint regions (−18 to −44 bp upstream of splice acceptor). LabRanchoR and BPHunter were the best branchpoint-specific tools in our evaluation and can also be considered. SpliceAI and Pangolin were the most effective at scoring acceptor-associated variants (splice acceptor creating or polypyrimidine tract variants upstream of cryptic splice acceptors). Including other sequence-based deep learning models that use smaller sequence contexts did not provide additional value. Intronic variants affecting splicing regulatory elements within cryptic exons are hard to predict. We endorse using SpliceAI with larger windows surrounding the variant site (setting the distance parameter to the maximum). In addition, classical approaches such as HEXplorer might come in handy for specific cases, such as assessing the potential impact of a variant on exon-defining regulatory motifs. Finally, SpliceAI-inspired models (Pangolin, CI-SpliceAI) and models that incorporate SpliceAI predictions as features (ConSpliceML, AbSplice-DNA, PDIVAS) can effectively predict new splice donor and donor-downstream variants. However, to keep the number of different tools to use to a minimum, we suggest using the original SpliceAI model.

Nonetheless, it is noteworthy to mention the impacts of using precomputed scores as the strategy for variant prioritization. The current version of SpliceAI precomputed scores (v1.3.1) does not include predictions for insertions and deletions larger than 1 and 4 nucleotides, respectively. In addition, the limit of 50 bp as the distance around the variant site to extract variant effects prevents SpliceAI from identifying other variant classes, such as exon skipping, when the variant exerts its effects at more than 50 base pairs from the affected exon.

Finally, when interpretable outcomes are important, the choice of the strategy may depend on the use case. There is currently no option covering all possible missplicing scenarios, and each method assesses interpretability differently. SpliceVault is a recently published web application that is effective when interpreting intronic variants leading to exon (or multiexon) skipping or partial intron retention through activation of preexisting cryptic splice sites. Alternatively, SQUIRLS can be applied, as the software is well designed, is thoroughly documented and generates HTML reports that practitioners can intuitively inspect. Nonetheless, it does not handle pseudoexon activation consequences properly (for that, SPiP is recommended). In addition, it should not be solely relied upon as a prediction tool, as it is not as performant as other models.

### Final remarks

We comprehensively assessed functional intronic variation occurring far from annotated splice sites. As a result, we make available to the community region-specific datasets that can be used to evaluate new models on variants whose molecular consequence is known. These datasets will assist developers in identifying potential limitations of the model and highlighting variant types that it is more prone to fail on. Additionally, we encourage developers to make their models publicly available by sharing them on open-source platforms to facilitate their reuse [64, 103].

Sequence-based models based on convolutional neural networks architectures are still the state-of-the-art approach for splicing variant prediction. However, the artificial intelligence field is rapidly evolving, and we have seen the emergence of Transformer-based architectures being applied to other variant effect prediction tasks, for example, effects on gene expression [104] or on protein function using large protein language models [105]. As a result, increasingly complex models are expected to effectively tackle open questions in splicing regulation, such as better capturing the synergistic effects of splicing regulatory elements. However, the community must be aware of the possible implications these models bring, such as a lack of transparency and decreased ability to generate mechanistic hypotheses.

## Methods

### Data collection and variant annotation

We employed the same variant annotation procedure for all the variants collected for this article (datasets described below). We used Ensembl VEP v109 [106] for the task, and transcript annotations were added accordingly (with “–per_gene –pick_order ccds,canonical,biotype,rank –no_intergenic –gencode_basic” set). We used variants in the GRCh37 genome build simply because several of the tools we include in the article do not support the GRCh38 genome build. Nonetheless, we provide all the datasets and predictions in both GRCh37 and GRCh38 (via liftOver) versions.

#### ClinVar

We downloaded ClinVar v202204 and selected all the SNVs for downstream analysis. We kept variants with Pathogenic and Benign assignments (“CLNSIG”== Pathogenic or Likely_pathogenic or Benign or Likely_benign). We identified intronic variants based on Ensembl VEP annotations: only variants with at least 1 intronic consequence (“INTRON” == 1) in a protein-coding transcript (“BIOTYPE” = protein_coding) were retained. Additionally, we excluded variants with exonic annotations in any other gene (“EXON” ≠ 1). To avoid being overly conservative, we added variants that Ensembl VEP annotated as being outside the gene body for the picked consequence (“Consequence” == TF_binding_site_variant or downstream_gene_variant or upstream_gene_variant or regulatory_region_variant) but that are annotated with intronic ontology terms in the “MC” field in the original VCF. To minimize labeling errors, we excluded variants with less than 1 confidence star. To ensure that the number of benign variants did not exceed 50,000 (and therefore avoid the dataset being excessively unbalanced), we selected all higher-confidence benign variants (with 2 or more stars, *N* = 13,093) along with 36,907 randomly chosen 1-star variants. Finally, we retrieved the RefSeq transcript ID associated with each variant and selected only those that were intronic in such reference transcript. The dataset size for raw evaluations amounted to 18,446 pathogenic and 49,343 benign variants.

#### Disease-causing intronic variants affecting RNA splicing

This dataset refers to a high-quality variant set that we carefully curated to comply with the following criteria:

- Variant must locate at more than 10 bp from the nearest splice site.
- Variant was experimentally proven to affect normal RNA splicing.
- Variant does not necessarily lead to pseudoexon activation.

The previous curation effort from our lab [20] was updated for this article to include a comprehensive set of intronic variants identified after 2017. Therefore, the positive (disease-causing) set of variants used in this benchmark totals 242 (81 from Vaz-Drago et al. [20] and 161 from the new curation effort).

We used gnomAD v2.1 to generate a matched control set. First, we extracted all gnomAD variants occurring in a window of 500 bp surrounding the variants in the positive set and selected common records with a frequency higher 0.01 (1%) in the population, resulting in 1,128 variants. Then, we ran Ensembl VEP as previously described and retained the intronic variants annotated as occurring in one of the 148 unique genes of the positive set (*N* = 1,091). Moreover, we kept variants absent in ClinVar having the VCF filter field as “PASS” (*N* = 546). Finally, we randomly sampled 242 from this set.

#### Variants that affect RNA splicing

The third main dataset refers to variants that affect different mechanisms of splicing regulation, which may or may not lead to disease. We defined different molecular categories based on the location of the variant relative to the abnormal splicing event. We focused on deep intronic variants that lead to partial intron retention or pseudoexon activation. In cases where a variant leads to both pseudoexon activation and partial intron retention, we have assigned it to the pseudoexon activation group. Exceptionally, we included variants that affect the branchpoint motif (thus, closer to annotated splice acceptors) that include other types of splicing alterations such as exon skipping. We defined each category as follows:

- *Branchpoint associated*, for those variants occurring between −18 and 44 bp upstream (as used in [33]) of an annotated or cryptic splicing acceptor site and that create or disrupt any of the following adenine-branchpoint consensus motifs: YTNAY, YTNA, TNA, YNA [77].
- *Acceptor upstream*, referring to any variant that locates between −2 and −18 bp upstream of the cryptic splice acceptor (including the polypyrimidine tract).
- *New splice acceptor*, denoting the variants that occur at the cryptic splice acceptor positions, including the first nucleotide of the cryptic exon.
- *Exonic-like*, for any variant occurring within the cryptic exon (pseudoexon or partially retained intron).
- *New splice donor*, composed of variants located at the cryptic splice donor positions, including the last position of the cryptic exon.
- *Donor downstream*, referring to any deep intronic variant that locates at a distance of more than 2 bp from the activated cryptic splice donor.

We used data produced or gathered from multiple studies to assign variants to each category (Table 2). While splicing-altering variants were straightforward to assign (based on source data, the functional consequence, and distances to the splicing element considered), we distributed the nonaltering variants such that they resembled as best as possible the spatial distribution of the positive sets. Hence, we assigned the negative variants taking into account 2 levels of information: the primary group (partial intron retention, pseudoexon activation) and the region category.

To keep in line with the expected biology, we assigned the variants that were within a defined distance to a splice site to the partial intron retention group and deeper intronic variants to the pseudoexon group. We used different distance thresholds for splice acceptors and donors (100 bp and 20 bp, respectively) so that the datasets were reasonably balanced. As for the region category, we defined negative variants occurring between 18 and 44 bp upstream of an annotated splicing acceptor as branchpoint-associated variants. We assigned as acceptor-upstream or donor-downstream the remaining intronic variants according to whether they were located upstream or downstream of the nearest annotated splice site. Because pseudoexons tend to resemble authentic exons [85], we exceptionally assigned exonic variants that did not change inclusion levels of tested exons [86] as controls for the exonic-like category.

Lastly, we generated control datasets for the new splice site categories. Splice site variants (located at one of the dinucleotide positions) that were experimentally tested to not affect splicing are not easily accessible. Therefore, to mimic the positive set, we looked for common deep intronic SNVs (>5% in gnomAD v2.1) in protein-coding transcripts that generate the most common 5-mer acceptor motif CAGGT in the human genome [14] through a mutation in the core splice site dinucleotide. We randomly selected 64 variants to match the number of positive new splice acceptor variants exactly. We employed the same procedure for the new splice donor variants, where we kept the variants that generate the most common 6-mer donor motif GGTAAG in the human genome [14] at the GT position. Finally, we selected 197 variants at random to match the number of positive new splice donor variants. We confirmed using Snaptron [107] that in GTEx data, there is no evidence that a splice junction is used at the variant intervals.

### Prediction tools

We selected an extensive list of prediction tools for evaluation. The single criterion for the inclusion of a tool was that it had to be designed to predict (at least partially) intronic variation. When available, we used precomputed scores to annotate our variant sets (from dbNSFP v4.0b1 [108], UCSC genome browser [109], Zenodo, or tool website). Otherwise, we ran the models directly following the developer’s instructions. For a subset of splicing-related tools (MMSplice, HAL, kipoiSplice4), we employed kipoi v0.8.6 [64] to get predictions. We additionally included splicing-related tools that predict specific splicing signals (e.g., BP) and are not necessarily targeted to predict pathogenicity. Because most of these tools do not score variants by design, and some require using a web-based portal, we developed a simple utility to prepare their input given a VCF file. Moreover, we created a script for each tool to process the raw output into a final prediction score to be included in a VCF file. The package is available at [82]. We annotated the final VCF files with all the predictions using vcfanno v0.3.3 [110]. We describe all the tools, their reference thresholds and how we ran them in Table 1.

### Performance evaluation

We used VETA v0.7.8 [111] to perform all the performance evaluations. We extended VETA’s feature set by implementing a new mode (“–do_intronic_analysis”) targeted to intronic variants. This option assigns intronic variants into distance bins based on their distance to the closest splice site. Accordingly, VETA seamlessly integrates per-bin analyses, allowing automatic inspection of how tool performance varies as one moves deeper into the intronic space. Moreover, VETA includes an *interrogate* mode that ranks candidate variants according to tool predictions, facilitating the downstream variant interpretation task in whole-genome and exome studies.

In this article, we employed different metrics according to the nature of the dataset and the goal of the analysis. Despite this, VETA generated confusion matrices for all tools. True positives (TP) indicates the number of pathogenic/splicing-altering variants that a tool correctly predicts as pathogenic (or splicing-altering). True negatives (TN) is the number of benign (or non-splicing-altering) variants that a tool scores as such. False negatives (FN) refers to the number of true pathogenic/splicing-altering variants that a tool predicts to be benign/non-splicing-altering. Finally, false positives (FP) stands for the number of benign/non-splicing-altering variants that a tool scores as pathogenic (or splicing-altering). For ClinVar data, we ranked variants based on the F1-score, given the unbalanced nature of the data (much more deep intronic benign variants than pathogenic). Because some tools do not score deep in the introns (missing data), we weighted the F1-score with the prediction coverage: $Coverage \cdot \left(2 \cdot \frac{(Precision \cdot Recall)}{(Precision + Recall)}\right)$ where $Coverage = \frac{Scored\_variants}{Total\_variants}$, $Precision = \frac{TP}{TP + FP}$, and $Recall = \frac{TP}{TP + FN}$. For balanced datasets, we ranked tools using a slight variation of the MCC ($MCC = \frac{TP \cdot TN - FP \cdot FN}{\sqrt{(TP+FP)(TP+FN)(TN+FP)(TN+FN)}}$) that normalizes the metric range between 0 and 1 ($normalizedMCC = \frac{MCC +1}{2}$). We weighted the normalized MCC values with the prediction coverage ($weighted\_normalized\_MCC = Coverage \cdot normalizedMCC$). Additionally, we employed ROC and PR curves for the comparisons that measure performance at multiple threshold values. To summarize such analyses, we used the auROC and the auPRC metrics, respectively.

### Further inspection of deep intronic variants in ClinVar

We selected ClinVar variants assigned to the “501–1000” and “+1000” intronic bins and used VEP to perform reannotation. We ran VEP using RefSeq annotations without picking any consequence (“–per_gene” and “–pick_order” were not set), meaning that all transcript consequences associated with each variant were retained. We employed a filter to only keep annotations of protein-coding transcripts. Then, we assigned each variant to 1 of 4 categories, according to the overlap configuration of transcripts belonging to the gene associated with the variant: if a variant is exonic in another overlapping transcript, we termed it as “exonic”; if a variant is located at a shorter distance from the splice site in any other transcript, we assigned the category “>1 transcript (smaller offset)”; if the distance to the closest splice site remains the same for all transcripts overlapping the variant, we assigned the variant to the “>1 transcript (smaller offset)” category; lastly, if no other transcript overlapped with the variant (besides the one used in the analysis), we set it to the “No other transcript” category.

### Threshold analysis for deep intronic variants

To derive clinically applicable prediction thresholds for deep intronic variants, we employed the same strategy we recently described [111]. Briefly, for each tool, we applied the F-Beta formula (at 3 different Beta values) over 100 threshold values uniformly distributed between the range of scores. The threshold that maximized the F-Beta function was selected. To evaluate the reliability of the adjusted thresholds, we used a bootstrapping procedure, where we kept the same ratio of pathogenic and benign variants as in the original dataset in each bootstrap. This analysis was conducted using VETA, with the options “–do_threshold_analysis” and “–bootstrapping” enabled.

### Assessing quality of interpretations for SPiP, SQUIRLS, and SpliceVault

For this task, we employed the dataset of pathogenic splicing variants used throughout the study. It includes variants from our curation plus variants from Vaz-Drago et al. [20] because the molecular mechanism for the splicing defect is known for almost all records (Supplementary Table S3). For SPiP and SQUIRLS, we ran VETA in the *interrogate* mode (with “*–labels Pathogenic*” set) to list the variants correctly predicted by each tool using the threshold calibrated for noncanonical intronic variation (SPiP >0.009 and SQUIRLS >0.016). We removed variants for which the ground-truth information was not available (e.g., pseudoexon-activating variants that lack details of the location of the variant concerning the cryptic event).

For SPiP, we parsed the output so that the interpretation tag, confidence interval, and original score were retrieved (third, fourth, and fifth fields after splitting predictions by “|”). We assigned variants with an “NTR” tag (low probability of affecting splicing, yet correctly predicted as pathogenic according to the calibrated threshold) to the “No interpretation” category. Variants not associated with any particular splicing mechanism (according to SPiP, the “Alter by complex event” tag) were given the “Not informative” interpretation category. Then, for each of the remaining SPiP tags, we classified the interpretation as correct if they matched the ground-truth information:

- “Alter BP” for variants associated with the branchpoint signal, else incorrect.
- “Alter by create new Exon” for variants that trigger pseudoexon activation, else incorrect.
- “Alter by create New splice site” for variants that create a new splice site or activate a nearby existing cryptic splice site, regardless of the variant leading to pseudoexon activation or partial intron retention, else incorrect.
- “Alter ESR” for intronic variants occurring within the boundaries of a new pseudoexon, else incorrect.
- “Alter by MES (Poly TC)” for polypyrimidine tract variants, else incorrect.

As for SQUIRLS, we ran the model for the subset of pathogenic variants correctly predicted by the tool using “–output-format html” and “–n-variants-to-report 121.” Afterward, we manually inspected the HTML report generated to derive structured interpretations for each variant: “Not informative” if the short description of the variant effect was not generated, “No interpretation” if SQUIRLS did not produce any description or figure for the variant, “New cryptic acceptor” and “New cryptic donor” if SQUIRLS described the creation of a new splice site and the variant was located at one of the splice site positions (based on the Sequence trekker figure) defined in this article, and “Activate cryptic acceptor” and “Activate cryptic donor” if SQUIRLS described the creation of a cryptic splice site and the variant was located outside of the splice site positions (based on the Sequence trekker figure). Because SQUIRLS does not predict the exact molecular effect of a splicing variant, we ignored the predicted number of bases affecting the coding sequence as this was not applicable for pseudoexon-activating variants. After manually inspecting the HTML report and generating structured interpretations, we classified the interpretation as correct if it matched the ground-truth information:

- “New splice acceptor” for variants that create a new splice donor, else incorrect.
- “New splice donor” for variants that create a new splice acceptor, else incorrect.
- “Activate cryptic acceptor” for variants located upstream of an existing cryptic splice acceptor and not associated with the branchpoint signal, else incorrect.
- “Activate cryptic donor” for variants located downstream of an existing cryptic splice donor, else incorrect.

Finally, for SpliceVault we did not run a model to get correctly predicted variants to further inspect. Rather, SpliceVault is a web portal [112] (last accessed 21 May 2023) to query noncanonical splicing patterns in large-scale population-based RNA sequencing data. Because it relies on querying rare missplicing events with respect to annotated exons, we excluded all variants that trigger pseudoexon activation, as SpliceVault cannot identify this class of events. As a result, 37 variants were left for evaluation. For each variant, we used the associated gene, intron number, and molecular effect to select the correct exon and splice site to look for. We used the hg38 version (300k-RNA) and changed the default SpliceVault settings so that the top 10 events per query were shown. Moreover, we allowed for all cryptic events to be reported, regardless of their distance to the target exon. We assigned variants to the “No interpretation” tag if the cryptic splicing event was not observed in SpliceVault top 10 events. Then, we classified the interpretation as “Correct” if any of the cryptic splicing triggered by the variant was observed within the top 4 events. This threshold was recommended by the authors of SpliceVault for clinical purposes. Conversely, if the event appeared in lower ranks, we classified the interpretation as “Incorrect.”

### Tissue-specific predictions by AbSplice-DNA

Throughout the article, we selected the maximum AbSplice-DNA prediction for any tissue to evaluate model performance. In contrast, for this analysis, we used all predictions so that tissue specificity could be addressed. We used the same dataset as for the interpretability section. We ran VETA in the *interrogate* mode (with “*–labels Pathogenic*” set) to list the variants correctly predicted by AbSplice-DNA using the threshold adjusted for noncanonical intronic variation (>0.004, in at least 1 tissue). Then, for each variant, we gathered information about the tissues associated with the disease by searching the HPO [90] with the given OMIM disease identifier. We strove to assign tissue names that matched the GTEx tissues used by AbSplice-DNA. Disease-causing variants affecting tissues not represented in GTEx (e.g., retina) were discarded. Additionally, variants causing systemic diseases (e.g., Marfan syndrome) or diseases returning ambiguous HPO terms were excluded.

## Availability of Supporting Source Code and Requirements

- Project name: VETA
- Project description: Software used to perform most of the analysis in the article
- Project home age: https://github.com/PedroBarbosa/VETA
- Operating system(s): Platform independent
- Programming language: Python
- License: GPL-3.0.
- RRID: SCR_023314
- biotoolsID: veta_variantBenchmark

- Project name: PrepareSplicingPredictors
- Project description: Utilities to generate input/process output of several sequence-based splicing predictors
- Project homepage: https://github.com/PedroBarbosa/Prepare_SplicingPredictors
- Operating system(s): Platform independent
- Programming language: Python
- License: GPL-3.0.
- RRID: SCR_023316

## Supplementary Material

giad085_GIGA-D-23-00047_Original_Submission

giad085_GIGA-D-23-00047_Revision_1

giad085_GIGA-D-23-00047_Revision_2

giad085_GIGA-D-23-00047_Revision_3

giad085_GIGA-D-23-00047_Revision_4

giad085_GIGA-D-23-00047_Revision_5

giad085_Response_to_Reviewer_Comments_Original_Submission

giad085_Response_to_Reviewer_Comments_Revision_1

giad085_Response_to_Reviewer_Comments_Revision_2

giad085_Response_to_Reviewer_Comments_Revision_3

giad085_Response_to_Reviewer_Comments_Revision_4

giad085_Reviewer_1_Report_Original_Submission Jeanâ€′Madeleine de Sainte Agathe -- 3/20/2023 Reviewed

giad085_Reviewer_1_Report_Revision_1 Jeanâ€′Madeleine de Sainte Agathe -- 6/14/2023 Reviewed

giad085_Reviewer_2_Report_Original_Submission Raphael Leman -- 4/14/2023 Reviewed

giad085_Reviewer_2_Report_Revision_1 Raphael Leman -- 6/22/2023 Reviewed

giad085_Supplemental_Files

## Data Availability

The datasets, supplementary material, and steps to reproduce all the results of this article are available in GitHub [113]. Supporting data, including variant sets, figures, and tables, are also available via the *GigaScience* repository, GigaDB [114].

## Additional Files

**Supplementary Table S1**. A summary of the ClinVar variants used in the study, including the intronic bins they were assigned to.

**Supplementary Table S2**. Performance statistics at each intronic bin.

**Supplementary Table S3**. Pathogenic splicing-altering variants curated for this study (it includes Vaz-Drago et al. [20] variants).

**Supplementary Table S4**. Performance statistics for the manual curated dataset of splicing pathogenic mutations (this study plus Vaz-Drago et al. [20]).

**Supplementary Table S5**. Optimized thresholds that maximize performance for noncanonical intronic splicing variants at different levels of importance given to precision and recall.

**Supplementary Table S6**. Multiple splicing-associated datasets evaluated in this study.

**Supplementary Table S7**. Performance evaluation results for all variants associated with splicing alterations.

**Supplementary Table S8**. SpliceAI lookup output (with max distance = 500) for the exonic-like splicing-altering variants that were missed by SpliceAI using precomputed scores.

**Supplementary Table S9**. SpliceVault output for variants used in the analysis.

**Supplementary Table S10**. Data used to analyze tissue specificity of AbSplice-DNA.

**Supplementary Fig. S1**. Intronic variant prediction in ClinVar. (A) Performance of all tools considered for the study on the raw ClinVar intronic dataset. Mean values in the legend represent the average weighted F1-score across all intronic bins. (B) Inspection of intronic variants (after addressing circularity problems) assigned to the “501–1000” and “1000+” intronic bins. The bars reflect the number of variants assigned to each category. The term “No other transcript” refers to all variants that do not have any other RefSeq protein coding transcript of the same gene overlapping with them, besides the transcript originally used (*N* pathogenic = 25, *N* benign = 340). “>1 transcript (same offset)” refers to variants that overlap with more than 1 transcript of the same gene but do not have any other transcript where the variant is closer to the splice site than in the original transcript used in the analysis (*N* pathogenic = 31, *N* benign = 527). “>1 transcript (smaller offset)” refers to variants that overlap with more than 1 transcript of the same gene and have at least 1 other transcript in which the variant is closer to the splice site than in the original transcript used in the analysis (*N* pathogenic = 20, *N* benign = 254). “Exonic” refers to variants that overlap with more than 1 transcript of the same gene and have at least 1 other transcript where the variant is exonic (*N* pathogenic = 41, *N* benign = 263). (C) Distribution of the updated intronic distances to the closest splice site for variants assigned to the “>1 transcript (smaller offset)” category. (D) Tool performance (measured with weighted F1-score) for each individual category. Tools with performance higher than 0.6 are highlighted.

**Supplementary Fig. S2**. Manually curated dataset of pathogenic intronic variants disrupting RNA splicing. (A) Number of variants collected per phenotype. Diseases with fewer than 3 variants were assigned to the “Other” category. (B) Number of variants occurring in ClinVar and gnomAD v2.1. (C) Log-transformed allele frequencies of variants in gnomAD v2.1. For those that are absent in the database, a pseudocount of 0.00001 was added (highest histogram peak, at 5). (D) Tool performance using reference thresholds from Table 1 for variants curated in this article plus those curated by Vaz-Drago et al. [20]. MLCsplice and dbscSNV are not shown as they had more than 95% of missing predictions. (E) Distance (log_10_) of the variants to the closest splice junction. Pseudoexon activation group: 194 variants; partial intron retention group: 37 variants.

**Supplementary Fig. S3**. Precision–recall curves for all splicing-altering variants analyzed in a region-specific manner. Tools are ranked by the auPRC metric, and the number of predictions made by each tool is displayed in “*n*=.” The number of variants in each dataset is presented (“*N* pos” represents the number of positive splicing-altering variants; “*N* neg” is the number of negative splicing variants). Tools with more than 50% of missing predictions or with fewer than 15 variants in the minority class were excluded from these analyses. For the exonic-like category, PDIVAS was excluded because control variants in this comparison are exonic, which is outside of the scope of this model. (A) Branchpoint-associated variants. (B) Acceptor-associated variants that trigger pseudoexon inclusion. (C) Acceptor-associated variants that lead to partial intron retention. (D) Exonic-like variants that trigger pseudoexon inclusion. (E) Exonic-like variants that lead to partial intron retention. (F) Variants that create new splice donors and activate pseudoexons. (G) Variants that create new splice donors and lead to partial intron retention. (H) Variants that activate existing upstream cryptic splice donors and trigger pseudoexon activation. (I) Variants that activate existing upstream cryptic splice donors and lead to partial intron retention.

**Supplementary Fig. S4**. Comparing performance between all pseudoexon activation versus partial intron retention variants collected in this study. (A) Distribution of the auPRC scores of the tools for each variant region. (B) Per-tool auPRC distribution across the 4 variant regions considered. A Fisher’s exact test was conducted separately for each tool and variant region to determine statistical significance for the performance differences between the pseudoexon activation and partial intron retention groups. The true positives plus true negatives were considered successful outcomes, while false positives plus false negatives were considered failures. The *P* values displayed in the figure were corrected for multiple comparisons using the Holm method. For each tool, we excluded the variant regions that did not have performance measurements in both groups.

**Supplementary Fig. S5**. Interpretability assessment. (A) Distance of the variants to the activated cryptic splice site (relative to the first splice site position) in SpliceVault analysis for the variants assigned to the “No interpretation” tag. (B) Distribution of SPiP and SQUIRLS prediction values for each of the interpretation categories.

**Supplementary Fig. S6**. Tissue-specific predictions made by AbSplice-DNA for a set of disease-causing variants associated with aberrant splicing. (A) Disease variants associated with multiple GTEx tissues that displayed variable scores across tissues. (B) Disease variants with no tissue specificity. All tissues got the same AbSplice-DNA score. Disease variants associated with 1 or more GTEx tissues are displayed in a single heatmap annotation.

## Abbreviations

auPRC: area under the precision–recall curve; auRO: area under the receiving operating characteristic; BP: branchpoint; CAT: clinically accessible tissue; FN: false negatives; FP: false positives; GTEx: Genotype-Tissue Expression; HGMD: Human Gene Mutation Database; HPO: Human Phenotype Ontology; MCC: Matthews correlation coefficient; MPRA: massive parallel reporter assay; PR: precision–recall; PSSM: position-specific scoring matrix; RBP: RNA-binding protein; ROC: receiving operating characteristic; SNV: single-nucleotide variant; TN: true negatives; TP: true positives; VEP: Ensembl Variant Effect Predictor; VUS: variant of uncertain significance; WGS: whole-genome sequencing.

## References

[1] Cooper DN . Functional intronic polymorphisms: Buried treasure awaiting discovery within our genes. Hum Genom. 2010;4(5):284–8. 10.1186/1479-7364-4-5-284. PMC3500160 20650817

[2] Karczewski KJ, Francioli LC, Tiao G, et al. The mutational constraint spectrum quantified from variation in 141,456 humans. Nature. 2020;581(7809):434–43. 10.1038/s41586-020-2308-7. 32461654 PMC7334197

[3] Taliun D, Harris DN, Kessler MD et al. Sequencing of 53,831 diverse genomes from the NHLBI TOPMed Program. Nature. 2021;590(7845):290–9. 10.1038/s41586-021-03205-y. 33568819 PMC7875770

[4] Eilbeck K, Quinlan A, Yandell M. Settling the score: variant prioritization and Mendelian disease. Nat Rev Genet. 2017;18(10):599–612. 10.1038/nrg.2017.52. 28804138 PMC5935497

[5] Lord J, Baralle D. Splicing in the diagnosis of rare disease: advances and challenges. Front Genet. 2021;12:689892. 10.3389/fgene.2021.689892. 34276790 PMC8280750

[6] Wahl MC, Will CL, Lührmann R. The spliceosome: design principles of a dynamic RNP machine. Cell. 2009;136(4):701–18. 10.1016/j.cell.2009.02.009. 19239890

[7] Ward AJ, Cooper TA. The pathobiology of splicing. J Pathol. 2010;220(2):152–63. 10.1002/path.2649. 19918805 PMC2855871

[8] Wang GS, Cooper TA. Splicing in disease: disruption of the splicing code and the decoding machinery. Nat Rev Genet. 2007;8(10):749–61. 10.1038/nrg2164. 17726481

[9] Lim KH, Ferraris L, Filloux ME et al. Using positional distribution to identify splicing elements and predict pre-mRNA processing defects in human genes. Proc Natl Acad Sci. 2011;108(27):11093–8. 10.1073/pnas.1101135108. 21685335 PMC3131313

[10] Jaganathan K, Kyriazopoulou Panagiotopoulou S, McRae JF et al. Predicting splicing from primary sequence with deep learning. Cell. 2019;176(3):535–48. 10.1016/j.cell.2018.12.015. 30661751

[11] Desterro J, Bak-Gordon P, Carmo-Fonseca M. Targeting mRNA processing as an anticancer strategy. Nat Rev Drug Discov. 2020;19(2):112–29. 10.1038/s41573-019-0042-3. 31554928

[12] Anna A, Monika G. Splicing mutations in human genetic disorders: examples, detection, and confirmation. J Appl Genet. 2018;59(3):253–68. 10.1007/s13353-018-0444-7. 29680930 PMC6060985

[13] Ule J, Blencowe BJ. Alternative splicing regulatory networks: functions, mechanisms, and evolution. Mol Cell. 2019;76(2):329–45. 10.1016/j.molcel.2019.09.017. 31626751

[14] Sibley CR, Blazquez L, Ule J. Lessons from non-canonical splicing. Nat Rev Genet. 2016;17(7):407–21. 10.1038/nrg.2016.46. 27240813 PMC5154377

[15] Landrum MJ, Lee JM, Benson M, et al. ClinVar: improving access to variant interpretations and supporting evidence. Nucleic Acids Res. 2018;46(D1):D1062–7. 10.1093/nar/gkx1153. 29165669 PMC5753237

[16] Stenson PD, Mort M, Ball EV, et al. The human gene mutation database (HGMD®): optimizing its use in a clinical diagnostic or research setting. Hum Genet. 2020;139(10):1197–207. 10.1007/s00439-020-02199-3. 32596782 PMC7497289

[17] Lord J, Gallone G, Short PJ et al. Pathogenicity and selective constraint on variation near splice sites. Genome Res. 2019;29(2):159–70. 10.1101/gr.238444.118. 30587507 PMC6360807

[18] Blakes AJM, Wai HA, Davies I, et al. A systematic analysis of splicing variants identifies new diagnoses in the 100,000 Genomes Project. Genome Med. 2022;14(1):79. 10.1186/s13073-022-01087-x. 35883178 PMC9327385

[19] Ellingford JM, Ahn JW, Bagnall RD et al. Recommendations for clinical interpretation of variants found in non-coding regions of the genome. Genome Med. 2022;14(1):73. 10.1186/s13073-022-01073-3. 35850704 PMC9295495

[20] Vaz-Drago R, Custódio N, Carmo-Fonseca M. Deep intronic mutations and human disease. Hum Genet. 2017;136(9):1093–111. 10.1007/s00439-017-1809-4. 28497172

[21] Keegan NP, Wilton SD, Fletcher S. Analysis of pathogenic pseudoexons reveals novel mechanisms driving cryptic splicing. Front Genet. 2022;12:943044. 10.3389/fgene.2021.806946. PMC9218974 35754842

[22] Lek M, Karczewski KJ, Minikel EV, et al. Analysis of protein-coding genetic variation in 60,706 humans. Nature. 2016;536(7616):285–91. 10.1038/nature19057. 27535533 PMC5018207

[23] Dunham I, Kundaje A, Aldred SF et al. An integrated encyclopedia of DNA elements in the human genome. Nature. 2012;489(7414):57–74. 10.1038/nature11247. 22955616 PMC3439153

[24] Eraslan G, Avsec Ž, Gagneur J et al. Deep learning: new computational modelling techniques for genomics. Nat Rev Genet. 2019;20(7):389–403. 10.1038/s41576-019-0122-6. 30971806

[25] Cormier MJ, Pedersen BS, Bayrak-Toydemir P et al. Combining genetic constraint with predictions of alternative splicing to prioritize deleterious splicing in rare disease studies. BMC Bioinformatics. 2022;23(1):482. 10.1186/s12859-022-05041-x. 36376793 PMC9664736

[26] Kurosawa R, Iida K, Ajiro M, et al. PDIVAS: Pathogenicity predictor for deep-intronic variants causing aberrant splicing. medRxiv. 2023. https://www.medrxiv.org/content/10.1101/2023.03.20.23287464v2. 10.1186/s12864-023-09645-2 PMC10563346 37817060

[27] Wagner N, Çelik MH, Hölzlwimmer FR et al. Aberrant splicing prediction across human tissues. Nat Genet. 2023;55(5):861–70. 10.1038/s41588-023-01373-3. 37142848

[28] Zeng T, Li YI. Predicting RNA splicing from DNA sequence using Pangolin. Genome Biol. 2022;23(1):103. 10.1186/s13059-022-02664-4. 35449021 PMC9022248

[29] Strauch Y, Lord J, Niranjan M, et al. CI-SpliceAI—improving machine learning predictions of disease causing splicing variants using curated alternative splice sites. PLoS One. 2022;17(6):e0269159. 10.1371/journal.pone.0269159. 35657932 PMC9165884

[30] Frankish A, Diekhans M, Ferreira AM, et al. GENCODE reference annotation for the human and mouse genomes. Nucleic Acids Res. 2019;47(D1):D766–73. 10.1093/nar/gky955. 30357393 PMC6323946

[31] Weber LM, Saelens W, Cannoodt R, et al. Essential guidelines for computational method benchmarking. Genome Biol. 2019;20(1):125. 10.1186/s13059-019-1738-8. 31221194 PMC6584985

[32] Buchka S, Hapfelmeier A, Gardner PP, et al. On the optimistic performance evaluation of newly introduced bioinformatic methods. Genome Biol. 2021;22(1):152. 10.1186/s13059-021-02365-4. 33975646 PMC8111726

[33] Leman R, Tubeuf H, Raad S et al. Assessment of branch point prediction tools to predict physiological branch points and their alteration by variants. BMC Genomics. 2020;21(1):86. 10.1186/s12864-020-6484-5. 31992191 PMC6988378

[34] Tubeuf H, Charbonnier C, Soukarieh O, et al. Large-scale comparative evaluation of user-friendly tools for predicting variant-induced alterations of splicing regulatory elements. Hum Mutat. 2020;41(10):1811–29. 10.1002/humu.24091. 32741062

[35] Moles-Fernández A, Domènech-Vivó J, Tenés A, et al. Role of splicing regulatory elements and in silico tools usage in the identification of deep intronic splicing variants in hereditary breast/ovarian cancer genes. Cancers. 2021;13(13):3341. 10.3390/cancers13133341. 34283047 PMC8268271

[36] Riepe TV, Khan M, Roosing S, et al. Benchmarking deep learning splice prediction tools using functional splice assays. Hum Mutat. 2021;42(7):799–810. 10.1002/humu.24212. 33942434 PMC8360004

[37] Rowlands C, Thomas HB, Lord J, et al. Comparison of in silico strategies to prioritize rare genomic variants impacting RNA splicing for the diagnosis of genomic disorders. Sci Rep. 2021;11(1):20607. 10.1038/s41598-021-99747-2. 34663891 PMC8523691

[38] Ha C, Kim JW, Jang JH. Performance evaluation of spliceai for the prediction of splicing of NF1 variants. Genes. 2021;12(9):1308. 10.3390/genes12091308. 34573290 PMC8472818

[39] Li K, Luo T, Zhu Y, Huang Y et al. Performance evaluation of differential splicing analysis methods and splicing analytics platform construction. Nucleic Acids Res. 2022:50(16):9115–9126.. 10.1093/nar/gkac686. 35993808 PMC9458456

[40] Leman R, Parfait B, Vidaud D et al. SPiP: splicing prediction pipeline, a machine learning tool for massive detection of exonic and intronic variant effects on mRNA splicing. Hum Mutat. 2022;43(12):2308–23. 10.1002/humu.24491. 36273432 PMC10946553

[41] Li S, van der Velde KJ, de Ridder D, et al. CAPICE: a computational method for consequence-agnostic pathogenicity interpretation of clinical exome variations. Genome Med. 2020;12(1):75. 10.1186/s13073-020-00775-w. 32831124 PMC7446154

[42] Siepel A, Bejerano G, Pedersen JS et al. Evolutionarily conserved elements in vertebrate, insect, worm, and yeast genomes. Genome Res. 2005;15(8):1034–50. 10.1101/gr.3715005. 16024819 PMC1182216

[43] Li J, Zhao T, Zhang Y, et al. Performance evaluation of pathogenicity-computation methods for missense variants. Nucleic Acids Res. 2018;46(15):7793–804. 10.1093/nar/gky678. 30060008 PMC6125674

[44] Siepel A, Pollard KS, Haussler D. New methods for detecting lineage-specific selection. In: Apostolico A, Guerra C, Istrail S et al., eds. Research in Computational Molecular Biology. Lecture Notes in Computer Science. Berlin, Heidelberg: Springer; 2006:190–205.

[45] Dong C, Wei P, Jian X et al. Comparison and integration of deleteriousness prediction methods for nonsynonymous SNVs in whole exome sequencing studies. Hum Mol Genet. 2015;24(8):2125–37. 10.1093/hmg/ddu733. 25552646 PMC4375422

[46] Garber M, Guttman M, Clamp M et al. Identifying novel constrained elements by exploiting biased substitution patterns. Bioinformatics. 2009;25(12):i54–62. 10.1093/bioinformatics/btp190. 19478016 PMC2687944

[47] Davydov EV, Goode DL, Sirota M et al. Identifying a high fraction of the human genome to be under selective constraint using GERP++. PLoS Comput Biol. 2010;6(12):e1001025. 10.1371/journal.pcbi.1001025. 21152010 PMC2996323

[48] Shihab HA, Rogers MF, Gough J et al. An integrative approach to predicting the functional effects of non-coding and coding sequence variation. Bioinformatics. 2015;31(10): https:/doi.org/10.1093/bioinformatics/btv009. 10.1093/bioinformatics/btv009 PMC4426838 25583119

[49] Liu X, Wu C, Li C, Boerwinkle E. dbNSFP v3.0: a one-stop database of functional predictions and annotations for human non-synonymous and splice site SNVs. Hum Mutat. 2016;37(3):235–41. 10.1002/humu.22932. 26555599 PMC4752381

[50] 1000 Genomes Project Consortium, Auton A, Brooks LD, Durbin RM, et al. A global reference for human genetic variation. Nature. 2015;526(7571):68–74. 10.1038/nature15393. 26432245 PMC4750478

[51] Ionita-Laza I, McCallum K, Xu B, et al. A spectral approach integrating functional genomic annotations for coding and noncoding variants. Nat Genet. 2016;48(2):214–20. 10.1038/ng.3477. 26727659 PMC4731313

[52] Jagadeesh KA, Paggi JM, Ye JS et al. S-CAP extends pathogenicity prediction to genetic variants that affect RNA splicing. Nat Genet. 2019;51(4):755–63. 10.1038/s41588-019-0348-4. 30804562

[53] Smedley D, Schubach M, Jacobsen JOB et al. A whole-genome analysis framework for effective identification of pathogenic regulatory variants in Mendelian disease. Am J Hum Genet. 2016;99(3):595–606. 10.1016/j.ajhg.2016.07.005. 27569544 PMC5011059

[54] Huang YF, Gulko B, Siepel A. Fast, scalable prediction of deleterious noncoding variants from functional and population genomic data. Nat Genet. 2017;49(4):618–24. 10.1038/ng.3810. 28288115 PMC5395419

[55] Fokkema IFAC, van der Velde KJ, Slofstra MK, et al. Dutch genome diagnostic laboratories accelerated and improved variant interpretation and increased accuracy by sharing data. Hum Mutat. 2019;40(12):2230–8. 10.1002/humu.23896. 31433103 PMC6900155

[56] Rentzsch P, Schubach M, Shendure J et al. CADD-splice—improving genome-wide variant effect prediction using deep learning-derived splice scores. Genome Med. 2021;13(1):31. 10.1186/s13073-021-00835-9. 33618777 PMC7901104

[57] Yeo G, Burge CB. Maximum entropy modeling of short sequence motifs with applications to RNA splicing signals. J Comput Biol. 2004;11(2-3):377–94. 10.1089/1066527041410418. 15285897

[58] Shamsani J, Kazakoff SH, Armean IM et al. A plugin for the ensembl variant effect predictor that uses maxentscan to predict variant spliceogenicity. Bioinformatics. 2019;35(13):2315–17. 10.1093/bioinformatics/bty960. 30475984 PMC6596880

[59] Jian X, Boerwinkle E, Liu X. In silico prediction of splice-altering single nucleotide variants in the human genome. Nucleic Acids Res. 2014;42(22):13534–44. 10.1093/nar/gku1206. 25416802 PMC4267638

[60] Wang J, Zhang J, Li K, et al. SpliceDisease database: linking RNA splicing and disease. Nucleic Acids Res. 2012; ;40(Database issue):D1055–9. 10.1093/nar/gkr1171. 22139928 PMC3245055

[61] Xiong HY, Alipanahi B, Lee LJ, et al. The human splicing code reveals new insights into the genetic determinants of disease. Science. 2015;347(6218). 10.1126/science.1254806. PMC4362528 25525159

[62] Rosenberg AB, Patwardhan RP, Shendure J et al. Learning the sequence determinants of alternative splicing from millions of random sequences. Cell. 2015;163(3):698–711. 10.1016/j.cell.2015.09.054. 26496609

[63] Gelfman S, Wang Q, McSweeney KM, et al. Annotating pathogenic non-coding variants in genic regions. Nat Commun. 2017;8(1):236. 10.1038/s41467-017-00141-2. 28794409 PMC5550444

[64] Avsec Ž, Kreuzhuber R, Israeli J, et al. The Kipoi repository accelerates community exchange and reuse of predictive models for genomics. Nat Biotechnol. 2019;37(6):592–600. 10.1038/s41587-019-0140-0. 31138913 PMC6777348

[65] Lonsdale J, Thomas J, Salvatore M et al. The genotype-tissue expression (GTEx) Project. Nat Genet. 2013;45(6):580–5. 10.1038/ng.2653. 23715323 PMC4010069

[66] Cheng J, Nguyen TYD, Cygan KJ, et al. MMSplice: modular modeling improves the predictions of genetic variant effects on splicing. Genome Biol. 2019;20(1):48. 10.1186/s13059-019-1653-z. 30823901 PMC6396468

[67] Danis D, Jacobsen JOB, Carmody LC, et al. Interpretable prioritization of splice variants in diagnostic next-generation sequencing. Am J Hum Genet. 2021;108(9):1564–77. 10.1016/j.ajhg.2021.06.014. 34289339 PMC8456162

[68] Liu H, Dai J, Li K et al. Performance evaluation of computational methods for splice-disrupting variants and improving the performance using the machine learning-based framework. Brief Bioinform. 2022:23(5):bbac334. 10.1093/bib/bbac334. 35976049

[69] Sherry ST, Ward MH, Kholodov M, et al. dbSNP: the NCBI database of genetic variation. Nucleic Acids Res. 2001;29(1):308–11. 10.1093/nar/29.1.308. 11125122 PMC29783

[70] Cartegni L, Wang J, Zhu Z, et al. ESEfinder: a web resource to identify exonic splicing enhancers. Nucleic Acids Res. 2003;31(13):3568–71. 10.1093/nar/gkg616. 12824367 PMC169022

[71] Ke S, Shang S, Kalachikov SM et al. Quantitative evaluation of all hexamers as exonic splicing elements. Genome Res. 2011;21(8):1360–74. 10.1101/gr.119628.110. 21659425 PMC3149502

[72] Erkelenz S, Theiss S, Otte M et al. Genomic HEXploring allows landscaping of novel potential splicing regulatory elements. Nucleic Acids Res. 2014;42(16):10681–97. 10.1093/nar/gku736. 25147205 PMC4176321

[73] Takeda Ji, Fukami S, Tamura A, et al. IntSplice2: prediction of the splicing effects of intronic single-nucleotide variants using LightGBM modeling. Front Genet. 2021;12:701076. 10.3389/fgene.2021.701076. 34349788 PMC8326971

[74] Corvelo A, Hallegger M, Smith CWJ, et al. Genome-wide association between branch point properties and alternative splicing. PLoS Comput Biol. 2010;6(11):e1001016. 10.1371/journal.pcbi.1001016. 21124863 PMC2991248

[75] Zhang Q, Fan X, Wang Y, et al. BPP: a sequence-based algorithm for branch point prediction. Bioinformatics. 2017;33(20):3166–72. 10.1093/bioinformatics/btx401. 28633445

[76] Paggi JM, Bejerano G. A sequence-based, deep learning model accurately predicts RNA splicing branchpoints. RNA. 2018;24(12):1647–58. 10.1261/rna.066290.118. 30224349 PMC6239175

[77] Zhang P, Philippot Q, Ren W et al. Genome-wide detection of human variants that disrupt intronic branchpoints. Proc Natl Acad Sci. 2022;119(44):e2211194119. 10.1073/pnas.2211194119. 36306325 PMC9636908

[78] Zuallaert J, Godin F, Kim M et al. SpliceRover: interpretable convolutional neural networks for improved splice site prediction. Bioinformatics. 2018;34(24):4180–8.. 10.1093/bioinformatics/bty497. 29931149

[79] Naito T . Predicting the impact of single nucleotide variants on splicing via sequence-based deep neural networks and genomic features. Hum Mutat. 2019:40(9):1261–1269.. https:/doi.org/10.1002/humu.23794. 31090248 10.1002/humu.23794 PMC7265986

[80] Soemedi R, Cygan KJ, Rhine CL, et al. Pathogenic variants that alter protein code often disrupt splicing. Nat Genet. 2017;49(6):848–55. 10.1038/ng.3837. 28416821 PMC6679692

[81] Scalzitti N, Kress A, Orhand R et al. Spliceator: multi-species splice site prediction using convolutional neural networks. BMC Bioinformatics. 2021;22(1):561. 10.1186/s12859-021-04471-3. 34814826 PMC8609763

[82] Barbosa P .Preparing input for multiple splicing predictors. GitHub 2023. https://github.com/PedroBarbosa/Prepare_SplicingPredictors.

[83] Grimm DG, Azencott CA, Aicheler F et al. The evaluation of tools used to predict the impact of missense variants is hindered by two types of circularity. Hum Mutat. 2015;36(5):513–23. 10.1002/humu.22768. 25684150 PMC4409520

[84] Jung H, Lee KS, Choi JK. Comprehensive characterisation of intronic mis-splicing mutations in human cancers. Oncogene. 2021;40(7):1347–61. 10.1038/s41388-020-01614-3. 33420369 PMC7892346

[85] Petersen USS, Doktor TK, Andresen BS. Pseudoexon activation in disease by non-splice site deep intronic sequence variation—wild type pseudoexons constitute high-risk sites in the human genome. Hum Mutat. 2022;43(2):103–27. 10.1002/humu.24306. 34837434

[86] Adamson SI, Zhan L, Graveley BR. Vex-Seq: high-throughput identification of the impact of genetic variation on pre-mRNA splicing efficiency. Genome Biol. 2018;19(1):71. 10.1186/s13059-018-1437-x. 29859120 PMC5984807

[87] Cheung R, Insigne KD, Yao D, et al. A multiplexed assay for exon recognition reveals that an unappreciated fraction of rare genetic variants cause large-effect splicing disruptions. Mol cell. 2019;73(1):183–94. 10.1016/j.molcel.2018.10.037. 30503770 PMC6599603

[88] SpliceAI Lookup API . https://spliceailookup.broadinstitute.org/. Accessed 25 May 2023.

[89] Dawes R, Bournazos AM, Bryen SJ, et al. SpliceVault predicts the precise nature of variant-associated mis-splicing. Nat Genet. 2023;55(2):324–32. 10.1038/s41588-022-01293-8. 36747048 PMC9925382

[90] Köhler S, Gargano M, Matentzoglu N, et al. The human phenotype ontology in 2021. Nucleic Acids Res. 2021;49(D1):D1207–17.. https://doi.org/10.1093/nar/gkaa1043. 33264411 10.1093/nar/gkaa1043 PMC7778952

[91] Richards S, Aziz N, Bale S, et al. Standards and guidelines for the interpretation of sequence variants: a joint consensus recommendation of the American College of Medical Genetics and Genomics and the Association for Molecular Pathology. Genet Med. 2015;17(5):405–24. 10.1038/gim.2015.30. 25741868 PMC4544753

[92] Schoch K, Tan QKG, Stong N et al. Alternative transcripts in variant interpretation: the potential for missed diagnoses and misdiagnoses. Genet Med. 2020;22(7):1269–75. 10.1038/s41436-020-0781-x. 32366967 PMC7335342

[93] Canson D, Glubb D, Spurdle AB. Variant effect on splicing regulatory elements, branchpoint usage, and pseudoexonization: strategies to enhance bioinformatic prediction using hereditary cancer genes as exemplars. Hum Mutat. 2020;41(10):1705–21. 10.1002/humu.24074. 32623769

[94] Grodecká L, Buratti E, Freiberger T. Mutations of pre-mRNA splicing regulatory elements: are predictions moving forward to clinical diagnostics?. Int J Mol Sci. 2017;18(8):1668. 10.3390/ijms18081668. 28758972 PMC5578058

[95] Gebauer F, Schwarzl T, Valcárcel J, et al. RNA-binding proteins in human genetic disease. Nat Rev Genet. 2021;22(3):185–98. 10.1038/s41576-020-00302-y. 33235359

[96] Ching T, Himmelstein DS, Beaulieu-Jones BK, et al. Opportunities and obstacles for deep learning in biology and medicine. J R Soc Int. 2018;15(141):20170387. 10.1098/rsif.2017.0387. PMC5938574 29618526

[97] Novakovsky G, Dexter N, Libbrecht MW, et al. Obtaining genetics insights from deep learning via explainable artificial intelligence. Nat Rev Genet. 2023:24:125–137.. https://doi.org/10.1038/s41576-022-00532-2. 36192604 10.1038/s41576-022-00532-2

[98] Aicher JK, Jewell P, Vaquero-Garcia J, et al. Mapping RNA splicing variations in clinically-accessible and non-accessible tissues to facilitate Mendelian disease diagnosis using RNA-seq. Genet Med. 2020;22(7):1181. 10.1038/s41436-020-0780-y. 32225167 PMC7335339

[99] Smith C, Kitzman JO. Benchmarking splice variant prediction algorithms using massively parallel splicing assays. bioRxiv 2023. https:/doi.org/10.1101/2023.05.04.539398. 10.1186/s13059-023-03144-z PMC10734170 38129864

[100] de Sainte Agathe JM, Filser M, Isidor B et al. SpliceAI-visual: a free online tool to improve SpliceAI splicing variant interpretation. Hum Genom. 2023;17:7. 10.1186/s40246-023-00451-1. PMC9912651 36765386

[101] MobiDetails . https://mobidetails.iurc.montp.inserm.fr/MD. Accessed 27 May 2023.

[102] CI-SpliceAI Online Service . https://ci-spliceai.com/. Accessed 27 May 2023.

[103] Wolf T, Debut L, Sanh V, et al. HuggingFace’s transformers: state-of-the-art natural language processing. arXiv 2020 v5. https://github.com/huggingface/transformers. Accessed 28 May 2023.

[104] Avsec Ž, Agarwal V, Visentin D et al. Effective gene expression prediction from sequence by integrating long-range interactions. Nat Methods. 2021;18(10):1196–203. 10.1038/s41592-021-01252-x. 34608324 PMC8490152

[105] Meier J, Rao R, Verkuil R, et al. Language models enable zero-shot prediction of the effects of mutations on protein function. Advances in Neural Information Processing Systems 2021.34:29287–29303.. https://openreview.net/forum?id=uXc42E9ZPFs.

[106] McLaren W, Gil L, Hunt SE, et al. The ensembl variant effect predictor. Genome Biol. 2016;17(1):122. 10.1186/s13059-016-0974-4. 27268795 PMC4893825

[107] Wilks C, Gaddipati P, Nellore A, et al. Snaptron: querying splicing patterns across tens of thousands of RNA-seq samples. Bioinformatics. 2018;34(1):114–16. 10.1093/bioinformatics/btx547. 28968689 PMC5870547

[108] Liu X, Li C, Mou C et al. dbNSFP v4: a comprehensive database of transcript-specific functional predictions and annotations for human nonsynonymous and splice-site SNVs. Genome Med. 2020;12(1):103. 10.1186/s13073-020-00803-9. 33261662 PMC7709417

[109] Kent WJ, Sugnet CW, Furey TS et al. The human genome browser at UCSC. Genome Res. 2002;12(6):996–1006. 10.1101/gr.229102. 12045153 PMC186604

[110] Pedersen BS, Layer RM, Quinlan AR. Vcfanno: fast, flexible annotation of genetic variants. Genome Biol. 2016;17(1):118. 10.1186/s13059-016-0973-5. 27250555 PMC4888505

[111] Barbosa P, Ribeiro M, Carmo-Fonseca M, et al. Clinical significance of genetic variation in hypertrophic cardiomyopathy: comparison of computational tools to prioritize missense variants. Front Cardiovasc Med. 2022;9. 10.3389/fcvm.2022.975478. PMC9433717 36061567

[112] SpliceVault Portal . https://kidsneuro.shinyapps.io/splicevault/. Accessed 21 May 2023.

[113] Barbosa P . GitHub Repository for “Computational Prediction of Human Deep Intronic Variation.”. 2023. https://github.com/PedroBarbosa/DeepIntronic_Benchmark. 10.1093/gigascience/giad085 PMC10599398 37878682

[114] Barbosa P, Savisaar R, Carmo-Fonseca M, et al. Supporting data for “Computational Prediction of Human Deep Intronic Variation.”. GigaScience Database. 2023. 10.5524/102423. PMC10599398 37878682

