# Supplementary material for: Computational prediction of human deep intronic variation
Source: Gigascience. 2023 Oct 25;12:giad085. doi: 10.1093/gigascience/giad085 (PMC10599398; doi:10.1093/gigascience/giad085)
Supplement: giad085_GIGA-D-23-00047_Revision_2 [file giad085_giga-d-23-00047_revision_2.pdf]

GigaScience

Computational prediction of human deep intronic variation

--Manuscript Draft--

**Manuscript Number:**	GIGA-D-23-00047R2
**Full Title:**	Computational prediction of human deep intronic variation
**Article Type:**	Research
**Funding Information:**	Fundação para a Ciência e a Tecnologia (SFRH/BD/137062/2018) — Mr Pedro Barbosa<br>Fundação para a Ciência e a Tecnologia (EXPL/CCI-COM/1306/2021) — Dr Alcides Fonseca<br>Fundação para a Ciência e a Tecnologia (UIDB/00408/2020) — Not applicable<br>FEDER (045300) — Not applicable<br>Fundação para a Ciência e a Tecnologia (LISBOA-01-0247-FEDER- 1207 045915) — Dr Alcides Fonseca<br>La Caixa (LCF/PR/HR20/52400021) — Dr Maria Carmo-Fonseca<br>Fundação para a Ciência e a Tecnologia (UIDP/00408/2020) — Not applicable

Abstract:

The adoption of whole genome sequencing in genetic screens has facilitated the detection of genetic variation in the intronic regions of genes, far from annotated splice sites. However, selecting an appropriate computational tool to discriminate functionally relevant genetic variants from those with no effect is challenging, particularly for deep intronic regions where independent benchmarks are scarce.
In this study, we have provided an overview of the computational methods available and the extent to which they can be used to analyze deep intronic variation. We leveraged diverse datasets to extensively evaluate tool performance across different intronic regions, distinguishing between variants that are expected to disrupt splicing through different molecular mechanisms. Notably, we compared the performance of SpliceAI, a widely used sequence-based deep learning model, with that of more recent methods that extend its original implementation. We observed considerable differences in tool performance depending on the region considered, with variants generating cryptic splice sites being better predicted than those that potentially affect splicing regulatory elements. Finally, we devised a novel quantitative assessment of tool interpretability and found that tools providing mechanistic explanations of their predictions are often correct with respect to the ground truth information, but the use of these tools results in decreased predictive power when compared to black box methods.
Our findings translate into practical recommendations for tool usage and provide a reference framework for applying prediction tools in deep intronic regions, enabling more informed decision-making by practitioners.

**Corresponding Author:**	Alcides Fonseca, Ph.D.<br>Universidade de Lisboa Faculdade de Ciencias<br>Lisboa, PORTUGAL
**Corresponding Author Secondary Information:**	
**Corresponding Author's Institution:**	Universidade de Lisboa Faculdade de Ciencias
**Corresponding Author's Secondary Institution:**	
**First Author:**	Pedro Barbosa
**First Author Secondary Information:**	
**Order of Authors:**	Pedro Barbosa
	Rosina Savisaar

Powered by Editorial Manager® and ProduXion Manager® from Aries Systems Corporation

	Maria Carmo-Fonseca
	Alcides Fonseca
**Order of Authors Secondary Information:**	
**Response to Reviewers:**	Dear Editor and Reviewers,<br><br>Thank you for your previous reviews. We addressed all your feedback, and we detailed each change in the document submission_reviews_giga_science_2nd_round. Hope we met your expectations.
**Additional Information:**	
**Question**	**Response**
Are you submitting this manuscript to a special series or article collection?	No
**Experimental design and statistics**<br><br>Full details of the experimental design and statistical methods used should be given in the Methods section, as detailed in our Minimum Standards Reporting Checklist. Information essential to interpreting the data presented should be made available in the figure legends.<br><br>Have you included all the information requested in your manuscript?	Yes
**Resources**<br><br>A description of all resources used, including antibodies, cell lines, animals and software tools, with enough information to allow them to be uniquely identified, should be included in the Methods section. Authors are strongly encouraged to cite Research Resource Identifiers (RRIDs) for antibodies, model organisms and tools, where possible.<br><br>Have you included the information requested as detailed in our Minimum Standards Reporting Checklist?	Yes
**Availability of data and materials**<br><br>All datasets and code on which the conclusions of the paper rely must be	Yes

either included in your submission or deposited in publicly available repositories (where available and ethically appropriate), referencing such data using a unique identifier in the references and in the "Availability of Data and Materials" section of your manuscript.<br><br>Have you have met the above requirement as detailed in our Minimum Standards Reporting Checklist?	

Powered by Editorial Manager® and ProduXion Manager® from Aries Systems Corporation

Manuscript
Click here to access/download;Manuscript;main.pdf

GigaScience, 2017, 1–24

doi: xx.xxxx/xxxx
Manuscript in Preparation
Paper

PAPER

Computational prediction of human deep intronic variation

Pedro Barbosa [1,2,*], Rosina Savisaar [3], Maria Carmo-Fonseca [2] and Alcides Fonseca [1,†]

[1]LASIGE, Faculdade de Ciências da Universidade de Lisboa, Lisboa, Portugal and [2]Instituto de Medicina Molecular João Lobo Antunes, Faculdade de Medicina da Universidade de Lisboa, Lisboa, Portugal and [3]Mondego Science, Coimbra, Portugal

[*]pbarbosa@lasige.di.fc.ul.pt
[†]amfonseca@ciencias.ulisboa.pt

Abstract

The adoption of whole genome sequencing in genetic screens has facilitated the detection of genetic variation in the intronic regions of genes, far from annotated splice sites. However, selecting an appropriate computational tool to discriminate functionally relevant genetic variants from those with no effect is challenging, particularly for deep intronic regions where independent benchmarks are scarce.

In this study, we have provided an overview of the computational methods available and the extent to which they can be used to analyze deep intronic variation. We leveraged diverse datasets to extensively evaluate tool performance across different intronic regions, distinguishing between variants that are expected to disrupt splicing through different molecular mechanisms. Notably, we compared the performance of SpliceAI, a widely used sequence-based deep learning model, with that of more recent methods that extend its original implementation. We observed considerable differences in tool performance depending on the region considered, with variants generating cryptic splice sites being better predicted than those that potentially affect splicing regulatory elements. Finally, we devised a novel quantitative assessment of tool interpretability and found that tools providing mechanistic explanations of their predictions are often correct with respect to the ground truth information, but the use of these tools results in decreased predictive power when compared to black box methods.

Our findings translate into practical recommendations for tool usage and provide a reference framework for applying prediction tools in deep intronic regions, enabling more informed decision-making by practitioners.

Key words: Variant prediction; Introns; Abnormal splicing; Machine learning; Model interpretability

Background

Genetic variation plays a crucial role in understanding human disease and trait inheritance. Yet, for a long time, studies paid scant attention to variants in intronic gene regions [1], which were thought to harbor little functional variation. With the advent of whole genome sequencing (WGS), and the possibility to apply it at the population scale [2, 3], rare intronic variation can be identified at unprecedented levels. However, the sheer amount of candidate variants detected in the genome of an individual poses challenges for functional interpretation [4], particularly for variants affecting RNA splicing [5].

Splicing consists of removing introns from the primary transcript and is mediated by the spliceosome complex with the help of many RNA-binding proteins (RBPs) that recognize regulatory signals in exons and introns [6]. Splicing is tightly regulated across cell types and is sensitive to genetic variants occurring in *cis* (within the exons and introns of the splicing substrate) and in *trans* (within the genes encoding for splicing factors) [7]. It is estimated that 10 to 50% of all monogenic disease-causing variants affect pre-

Compiled on: July 7, 2023.
Draft manuscript prepared by the author.

mRNA splicing [8–10]. In addition, cancer driver mutations are often associated with splicing alterations, notably in the case of trans variants that occur in genes encoding core components of the splicing machinery [11].

Of the cis variants that affect splicing, those that disrupt the splice sites (typically AG for the 3'ss and GT for the 5' ss) or the consensus region around the splice sites (nucleotides -12/+2 around the 3'ss and -3/+6 around the 5'ss) have been studied the most thoroughly. Variants in these regions, especially if they affect the splice sites themselves, are fairly easy to recognize because the sequences are short and adhere to a highly conserved motif [12]. In contrast, other splicing variants can impact the binding of regulatory factors to splicing enhancers or silencers. These enhancers and silencers consist of short, poorly defined sequence motifs, which can occur at varying distances to the splice sites and can overlap either exons or introns [13]. It is thus difficult to identify them – and even more difficult to know when a mutation has disrupted them. Disruption of splicing information can lead to aberrant splice events such as exon skipping, full intron retention, or exon shortening or lengthening. Splicing variants can also create entirely new exons ("pseudoexons"). This can happen when a mutation creates a novel splice site, as well as when an existing but inactive ("cryptic") splice site is activated by the creation of an enhancer motif or the disruption of a silencer motif [14].

There have been continuous efforts to systematically catalog disease-causing variation in databases such as ClinVar [15] or the Human Gene Mutation Database (HGMD) [16]. These resources show the enrichment of splicing-related variants in the vicinity of splice site regions. Partly, this reflects a biological reality, where the sequence around the splice sites is particularly dense in splicing-relevant information. However, this enrichment may also stem from the easier detection of splice site mutations, as well as biases in clinical guidelines for variant interpretation that may contribute to underestimating the significance of non-canonical splicing mutations, as there is a lack of standardized criteria for their interpretation [17, 18].

Therefore, it is expected that many splicing variants in other gene regions remain to be discovered. Our dearth of knowledge is greatest deep inside the introns, where the detection problem is the hardest given the large search space and the fact that the rare splice-affecting variants are greatly outnumbered by mutations with no effect. As a result, deep intronic variants often end up labeled as Variant of Uncertain Significance (VUS) [19], although a subset may have great clinical importance. Indeed, recent evidence has shown that deep intronic mutations triggering pseudoexon activation are an overlooked cause of human disease [20, 21].

Given the additional challenges of interpreting deep intronic mutations, computational tools are often used to prioritize variants based on their likelihood of being deleterious. The first wave of methods used large genomics datasets to engineer features (e.g., allele frequencies from ExAC [22] or histone modification levels across cell lines from ENCODE [23]) and to build classifiers that work on tabular data. More recently, end-to-end deep-learning methods predict the impact of genetic variants from sequence alone, with the features automatically extracted within the network [24]. SpliceAI [10] is widely recognized as the most successful method of this kind, although its performance has been shown to vary across studies and datasets considered [5]. Recently, new models have been developed based on SpliceAI, either combining its predictions with other sources of information (such as genetic constraint for ConSpliceML [25] and PDIVAS [26] or tissue-specific splice site usage for AbSplice-DNA [27]) or creating an entirely new model based on SpliceAI architecture. For example, Pangolin [28] uses splicing quantifications from multiple species and tissues to not only predict whether a position is a splice site (as SpliceAI does) but also to predict splice site usage (e.g., how much a splice site is being used in a given tissue). In contrast, CI-SpliceAI [29] uses different training labels for true and false splice site positions based on a collapsed transcript structure derived from GENCODE [30] annotations.

Most intronic variant prediction benchmarking studies are performed by the authors of the tools to present a comparative analysis with existing methods. Even subconsciously, biases might be favoring the proposed model, be it because of the dataset selected or the methodology employed for the comparison [31, 32]. Multiple independent benchmark studies do exist [33–39], however, their scope is often somewhat limited. Firstly, some studies only focus on variants overlapping particular types of splicing information, e.g. splicing regulatory elements [33, 35]. Secondly, only using variants from a small number of genes can render the genome-wide extrapolation of conclusions difficult [34, 36, 38]. Lastly, to our knowledge, no study compares the performance of promising and recently developed methods such as Pangolin, CI-SpliceAI, ConSpliceML, AbSplice-DNA, PDIVAS and SPiP [40].

To help researchers and clinical practitioners understand prediction tools and how they can be applied to interpret genetic variants in introns, we conducted a comprehensive evaluation of a series of tools for the task of predicting functional variation in the intronic space far from canonical splice sites. To this end, we carefully selected intronic variants from multiple sources and curated a new set of disease-causing deep intronic variants affecting RNA splicing. Besides evaluating the capacity of tools to predict functional variants deep within the introns, we report, for the first time, an assessment of the interpretability of the output of these tools. We finally provide clear recommendations for tool usage depending on the variant's location within the intron and its molecular effect.

Results

The prediction tools studied are diverse in methodology and objectives

In this study, we have provided a snapshot of the state-of-the-art of methods that predict, in any way, functional variation in introns (Table 1). We divided the methods into four different categories: conservation scores that measure the degree of evolutionary conservation at a given position or region of the genome; genome-wide predictors that integrate multiple feature types to predict variant effects regardless of the variant type; methods that focus on splice-disrupting variants and allow for automated batch predictions; and splicing-specific methods that solely target specific types of splicing information (e.g., Branchpoint (BP)), or require the use of a web application to retrieve results. For many of the tools, there are two fundamentally different ways to obtain predictions: making *de novo* model inferences given an input variant set or using pre-computed predictions, which is faster computationally. We decided to use pre-computed predictions when available because it considerably simplifies the variant annotation pipeline and is thus accessible to a more diverse set of end users. However, it should be noted that this approach may miss some indels that are not represented in the pre-computed databases. Out of the 38 tools used to score at least one dataset in this paper, 19 had pre-computed databases available (Table 1). Because some of them only provide predictions for the GRCh37 genome build, we ran all experiments using this genome version. Of note, pre-computed predictions are a permanent representation of a model version, which may not be updated along with developments to the tool. However, we observed that only one tool, CAPICE [41], had outdated pre-computed scores.

Table 1. Summary of the computational methods used in this study

	Tool *	RRID	Threshold †	Description	Method	Training data	Predictions from ‡	Used in analysis **
Conservation	phastCons 100way [42]	-	> 0.99 [43]	Probability that each nucleotide belongs to a conserved element	Hidden Markov Model	Genomes of 100 vertebrates	Pre-computed (UCSC)	ClinVar
	phyloP 100way [44]	-	> 1.6 [45]	P-value that indicates how aligned sequences deviate from the null hypothesis of neutral evolution	Hidden Markov Model	Genomes of 100 vertebrates	Pre-computed (UCSC)	ClinVar
	SiPhy 29way [46]	SCR_000564	> 12.7 [45]	Identification of constrained sites as those with a nucleotide substitution pattern significantly deviating from the neutral pattern	Maximum Likelihood and Hidden Markov Model	Genomes of 29 mammals	Pre-computed (dbNSFP)	ClinVar
	GERP [47]	SCR_000563	> 4.4 [45]	Identification of evolutionarily constrained elements	Maximum Likelihood to estimate the evolutionary rate and dynamic programming	Genomes of 34 mammals	Pre-computed (UCSC)	ClinVar
Genome-wide predictors	FATHMM-MKL [48]	-	> 0.5 [49]	Prediction of functional consequences of coding and non-coding SNVs using genomic annotations from ENCODE and conservation scores	Support Vector Machine based on Multiple Kernel Learning	3,063 disease-implicated SNVs from HGMD; 5,252 negative instances from 1000G project [50]	Pre-computed (dbNSFP)	ClinVar
	Eigen v1.1 [51]	-	> 4.87 [52]	Unsupervised learning approach to leverage the functional importance of genetic variants across the whole genome	Linear combination of the components of the leading eigenvector determined from a rank-one matrix estimated from 3 genome-wide annotation blocks.	418,997 variants from 1000G project	Pre-computed (dbNSFP)	ClinVar
	ReMM v0.3.1 [53]	SCR_023095	> 0.984	Classifier to predict the potential of an arbitrary position in the genome to cause a Mendelian disease	Random Forest	453 disease-implicated variants by manual curation	Pre-computed (tool webpage)	ClinVar
	LINSIGHT [54]	-	> 0.056 [52]	Prediction of non-coding nucleotide sites at which mutations are likely to have deleterious fitness consequences	INSIGHT and Online stochastic gradient descent	Genomes of 54 unrelated human individuals	Pre-computed (dbNSFP)	ClinVar
	CAPICE v1.0 [41]	-	> 0.02	A consequence-agnostic method for pathogenicity prediction	XGBoost	Data from ClinVar, VKGL [55] and specific publication	Pre-computed (Zenodo)	ClinVar
	CADD-Splice v1.6 [56]	-	> 15 [45]	Prediction of the deleterious effect a variant has on an individual's fitness	Logistic Regression	16,627,775 of both proxy-neutral and proxy-deleterious variants	Pre-computed (tool webpage)	ClinVar; Splicing Pathogenic
Splicing	MaxEntScan [57]	SCR_016707	$\lvert \Delta Entropy \rvert > 3$	Prediction of RNA splice site signal based on the maximum entropy principle	Maximum Entropy distribution	8,500 real 5'SS and 3'SS; 180,000 decoy 5'SS and 3'SS	VEP plugin [58]	ClinVar; AU; NSD
	dbscSNV v1.1 [59]	-	> 0.6	*In silico* prediction of splice-altering variants based on an ensemble of individual methods	AdaBoost and Random Forest	Splice-altering variants from HGMD, SpliceDisease [60] and DBASS [60] databases. Negative variants from 1000G Project	Pre-computed (dbNSFP)	ClinVar; Splicing Pathogenic
	SPANR/SPIDEX v1.0 [61]	-	$\lvert \Delta PSI\_zscore \rvert > 2$	Prediction of how much SNVs cause splicing misregulation by measuring differential exon inclusion events	Bayesian Deep neural network	RNA-Seq data in 10,700 exons across 16 tissues	Pre-computed (tool webpage)	ClinVar; Splicing-Pathogenic; BP
	HAL [62]	SCR_022581	$\lvert \Delta PSI \rvert > 0.05$ §	Variant effect prediction on different isoform usage from alternative splicing events (alternative 5'ss and exon skipping)	Linear model using hexamer motif frequencies	Massive Parallel Reporter Assay (MPRA) containing 265,137 minigenes in a library of alternative 5' splice donors	Kipoi (only 5'ss model)	ClinVar; NSD; DD
	TrAP v3.0 [63]	-	> 0.174	Prediction of the damage caused by SNVs at the transcript level by incorporation of splicing-engineered features	Random Forest	75 pathogenic synonymous variants; 402 synonymous variants as benign	Pre-computed (tool webpage)	All
	S-CAP v1.0 [52]	-	Several thresholds ‖	Splicing-specific pathogenicity score derived from variant, exon and gene importance measurements	Gradient Boosting tree	17,059 splicing-related pathogenic variants from HGMD and Clinvar and 6,760,450 splicing region benign variants from gnomAD	Pre-computed (tool webpage)	ClinVar; Splicing-Pathogenic; BP
	KipoiSplice4 v0.1 [64]	-	> 0.5	Ensemble method that incorporates predictions from 4 splicing-related models (HAL, MaxEntScan5, MaxEntScan3 and LaBranchoR)	Logistic Regression	10,715 splice region variants from Clinvar and 2,959 variants from the dbscSNV paper [59]	Kipoi	ClinVar; Splicing-Pathogenic; BP
	SpliceAI v1.3 [10]	-	> 0.2	Splice site prediction from primary sequence	Deep residual neural network	Primary transcript of 13,384 genes, accounting for 130,796 donor-acceptor pairs, plus novel splice junctions observed in the Genotype-Tissue Expression (GTEx) data [65]	Pre-computed (tool webpage)	All
	MMSplice v1.03 [66]	-	$\lvert \Delta logitPSI \rvert > 1$	Modular approach to study functional effects of variants on splicing	Linear model that combines coefficient of 5 neural network modules	MPRA (Vex-Seq) designed to evaluate the effect of 2059 ExAC variants on exon skipping#	Kipoi	ClinVar; Splicing-Pathogenic; BP
	SQUIRLS v2.0.1 [67]	-	> 0.074 [29]	Prediction of the effect of variants on splicing providing interpretable outputs	Logistic regression model combining predictions from two Random Forests classifiers (donor and acceptor)	Cytoband-aware split of 73,203 benign variants from ClinVar and 8,314 deleterious variants from ClinVar and manual curation of variants from literature	Model inference	All
	Pangolin v1.02 [28]	-	> 0.2	Splice site prediction from primary sequence across multiple tissues	Deep residual neural network	Sequences and splice site quantifications from four species: human, rhesus macaque, rat and mouse	Model inference	All
	CI-SpliceAI v1.0 [29]	-	> 0.190	Same as SpliceAI	Deep residual neural network	Sequences of 18,580 genes with splice sites (428,275) collapsed from GENCODE isoforms	Model inference	All
	ConSpliceML v0.0.6 [25]	-	> 0.5	Combination of SpliceAI and SQUIRLS predictions along with a metric of genetic constraint against deleterious splicing variation	Random Forest	18,317 splicing-altering HGMD variants plus benign de novo variants collected from whole genome sequencing studies and GTEx	Pre-computed (tool webpage)	All
	AbSplice-DNA v0.0.1 [27]	-	> 0.01	Aberrant splicing prediction using MMSplice, SpliceAI and tissue-specific annotations derived from GTEx	Generalized additive model	Splicing outliers detected from 946 GTEx individuals with paired RNA-Seq and WGS data	Pre-computed (Zenodo)	All
	MLCsplice [68]	-	> 0.5	Meta-predictor incorporating multiple splicing-related scores to predict region-specific variants	Hybrid model based on XGBoost, CGBoost and LightGBM	Positive variants obtained from DBASS and HGMD database. Negative variants retrieved from gnomAD, ExAC and dbSNP [69] with MAF > 10%	Pre-computed (tool webpage)	ClinVar; Splicing-Pathogenic; BP

	SPiP v2.1 [40]	-	> 0.452	Prioritization of splicing variants by running complementary bioinformatic tools that model different splicing elements	Random Forest	Random 50% split of 4,416 curated splicing-altering variants and 95,000 control variants	Model inference	All
	PDIVAS v1.0.0 [26]	-	> 0.151	Pathogenic prediction of deep intronic variation combining SpliceAI (including raw scores), MaxEntScan and ConSplice features	Random Forest	374 pathogenic variants from HGMD and Keegan et al. [21]; 153,794 benign variants from the 1000G project	Model inference [‡‡]	SplicingPathogenic; AU; EL; NSD; DD
Splicing (Region-specific or web-based)	ESEfinder v3.0 [70]	SCR_007088	$\|\Delta score\| > 0.5$ [***]	Identification of exonic splicing enhancers from weight matrices of four SR proteins derived from SELEX experiments	Scoring motifs of each SR protein against a predefined threshold (inferred from high-scoring randomly chosen sequences from the initial SELEX library)	-	Webpage & Own code	EL
	ESRseq [71]	SCR_022270	$\|\Delta score\| > 0.5$ [34]	QUEPASA, a minigene assay that measured the impact of 6-mer motifs in RNA splicing	Statistical comparison of observed splicing strengths in sequences where the motif is present vs absent	-	Own code	EL
	HEXplorer [72]	SCR_022269	$\|\Delta score\| > 14$ [34]	RESCUE-based approach to score elements that enhance or repress splice site usage	Average Z-score HZei (based on hexamer frequencies in exonic vs intronic sequences) of all six hexamers overlapping with any given nucleotide	-	Webpage & Own code	EL
	IntSplice2 v2.0 [73]	-	> 0.5	Prediction of pathogenic intronic SNVs upstream of splicing acceptors	LightGBM	1,787 of each class located at -50 to -3bp of splicing acceptors. Pathogenic variants from HGMD and ClinVar. Neutral variants from dbSNP.	Pre-computed (tool webpage)	SplicingPathogenic; BP; AU
	SVM-BPFinder [74]	-	$\|score\| > 0.136$ [33]	Branchpoint prediction using sequence signals and additional polypyrimidine tract features	Support Vector Machine	Positive sequences: intronic 9-mers conserved across multiple species. Negative sequences: random intronic 9-mers. Both sets had T and A at positions 4 and 6.	Model inference & Own code (as in [33])	BP
	BPP [75]	-	$\|score\| > 0.0006$ [33]	Branchpoint prediction using sequence features extracted from conserved intronic regions of the human genome	Mixture model to predict branchpoint motif combined with octanucleotide frequencies in PPT region	223,606 human introns longer than 300bp	Model inference & Own code (as in [33])	BP
	LaBranchoR [76]	-	$\|\Delta score\| > 0.1$	Prediction of splicing branchpoint signals from raw sequence	Bi-LSTM neural network	Highly confident branchpoints that matched GENCODE-annotated 3'ss	Kipoi	BP
	BPHunter v2[77]	-	> 1 [††]	Detection of intronic variants that disrupt the branchpoint sequence	Integration of Gradient Boosting tree, Random Forest and Logistic Regression with the majority voting for the final prediction	198,256 branchpoint positions with flanking 13-bp and 1 million 13-bp random intronic and exonic positions	Webpage & Own code	BP
	SpliceRover [78]	-	> 0.5 [***]	Splice site prediction from primary sequence	Convolutional neural network	Sequences of arabidopsis and human surrounding canonical splice donors and acceptors	Webpage & Own code	AU; NSD; DD
	DSSP [79]	-	> 0.5 [***]	Prediction of the impact of SNVs on splicing using a combination of deep learning and standard machine learning with handcrafted features	Stack generalization to combine a convolutional neural network with a Random Forest, XGBoost and Linear Regression models	170-bp sequences representing 4,964 variants (with corresponding wildtype sequences) from the MaPSy experiment [80]	Model inference & Own code	NSD
	Spliceator v1.0 [81]	-	> 0.5 [***]	Splice site prediction for multi-species data	Convolutional neural network	Sequences from multiple species, from protists to human	Model inference & Own code	AU; NSD; DD

[*] We refer to the specific tool version used, although for several tools we did not find a reference pointing to any version.
[†] Cutoff used to discriminate pathogenic/functional variants. If the original paper did not provide a reference threshold, it was extracted from elsewhere, with another reference assigned.
[‡] 'Own code'refers to our package [82].
[**] Analysis where tool was used. Where acronyms are seen, it refers to the region-specific splicing analysis: BP = Branchpoint-associated; NSA = New splice acceptor; NSD = New splice donor; AU = Acceptor upstream; DD = Donor downstream; EL = Exonic-like.
[§] HAL scores PSI for the sequence containing alternative 5'ss variants. Therefore, for this work, a change in PSI > 0.05 was defined as the relevant threshold.
[‖] S-CAP authors provide different reference thresholds depending on the location and context of the variant. 3intronic: 0.006, exonic: 0.009, 5intronic: 0.006, 5core_dominant: 0.034, 5core_recessive: 0.367, 5extended: 0.005, 3core_dominant: 0.033, 3core_recessive: 0.264.
[#] Several models were fitted in the MMSplice paper. In the table, the details of a single model are provided, the one that predicts *DeltalogitPSI* changes, as it was the primary goal defined by the authors.
[***] When no reported threshold was found, we set 0.5 as the default value.
[††] BPHunter threshold adjusted to 1 after discussing with the tool's author. Annotated variants with 0 score are shifted to 1, and all unannotated variants are assigned a score of 0.
[‡‡] PDIVAS does provide pre-computed scores, but those only include pathogenic predictions. To get scores for variants that are not predicted as pathogenic, we need to perform raw model inferences.

Importantly, not every tool considered was built with deep intronic regions in mind. For example, some tools were explicitly trained only to score consensus splice site variants (e.g. MaxEntScan [57], dbscSNV [59]), while others only output predictions up to an approximately defined distance between the variant and the nearest splice site (e.g., 300 bp for SPIDEX [61] or 50 bp for MLCSplice [68]). In addition, we ran certain models (KipoiSplice4 [64], HAL [62], MMSplice [66]) using the Kipoi framework [64], which further restricts predictions to a tool-specific distance between the splice site and the variant. Therefore, we expected these methods to perform poorly on some comparisons simply because the fraction of missing predictions should increase when moving further into the intron. Still, we decided to include these tools in the study because many of the variants evaluated locate within the distance that we expected these tools to cover.

It should also be noted that the tools were built for different tasks. While some models were designed to distinguish between pathogenic and benign variants (e.g., S-CAP [52], KipoiSplice4), others predict variant effects on splicing outcome, which does not necessarily translate into disease (e.g., SPiP, MMSplice). The latter category comprises sequence-based deep learning models such as SpliceAI or Pangolin. While these packages accept genetic variants in VCF format as input, it is important to note that the models primarily operate on sequences. They predict the probability of a given sequence position functioning as a splice site. If the model is run twice, once with the reference and once with the mutated sequence, it is possible to assess splice site alterations caused by genetic variants with the so-called delta score (mutated - reference allele). This has been the major practical use of the tool so far. Using the same approach, we also included several sequence-based methods that predict splicing-related elements. These include SpliceRover [78], DSSP [79], and Spliceator [81] for splice site-associated variants, ESEfinder [70], ESRseq [71] and HEXplorer [72] for variants affecting splicing regulatory elements, and SVM-BPFinder [74] and BPP [75] for variants impacting the BP signal. Of note, we only employed these methods for the datasets deemed to be relevant given their original task.

Intronic pathogenic variants located beyond 10 bp from the splice sites are poorly predicted

We employed a bin-based analysis to evaluate ClinVar data (Supplementary Table S1). Because ClinVar contains disease-causing variants that act through different molecular mechanisms, we included not only splicing-related tools but also conservation scores and whole genome predictors in the evaluations. Some of the models were trained using ClinVar data (Table 1), potentially leading to a circularity type I problem [83]. Fully correcting for this issue would have signified removing all ClinVar variants that were used in the training of any of the tools. This would have been problematic, as we would have lost many valuable deep intronic variants, which are typically scarce. However, most of the tools that were trained with ClinVar variants performed poorly. CAPICE was the only one to achieve a weighted F1 score above 0.6 across all bins (Supplementary Figure S1A). We therefore only removed ClinVar variants that were used for training CAPICE (N=14,189: 5,205 pathogenic and 8,984 benign). This is a trade-off, allowing for over-estimated performance for some of the more underperforming tools while ensuring a sufficiently large dataset for the evaluation of all tools. After this filtering step, 53,600 variants remained for evaluation. As expected, the distribution of the two variant classes (pathogenic and benign) across bins is highly unbalanced (Figure 1A). More than 90% of the intronic pathogenic variants occur at splice site positions, and more than 95% occur within 10 nucleotides from an exon-intron boundary.

Due to the spatial limitations discussed previously, we expected that some splicing tools would only output predictions for ClinVar variants located close to splice sites. Our results confirmed that several methods make predictions for less than 50% of the variants located at a distance of more than 40 bp from the nearest splice junction (Figure 1B). The fraction of predicted variants decreases according to the expected regions that each model covers: 50bp for S-CAP and MLCsplice, and 300bp for SPIDEX. MLCsplice was designed to predict non-canonical splicing variants (i.e. excluding splice site variants), thus, it is the only tool that displays no predictions at 1-2 positions (Figure 1B). In addition, we observed that the tools run using the Kipoi framework (KipoiSplice4, HAL, MMSplice) displayed a notable drop 41-200 bp from the splice site. On the other hand, SQUIRLS [67], Pangolin, CI-SpliceAI, SPiP, TraP [63], SpliceAI, ConSpliceML and AbSplice-DNA predicted across entire introns (Figure 1B). Regarding the remaining tool categories, we observed that both whole genome predictors and conservation scores (except phastCons [42]) output predictions for most ClinVar variants (Figure 1B).

Next, we evaluated how the tools that score across full introns perform with ClinVar data. Performance dropped considerably for variants located deeper in intronic regions, especially once a distance of 10 nucleotides from the splice site had been reached (Figure 1C). The splicing tools with the smallest and largest performance decrease between the splice site bin ("1-2") and the "11-40" bin were Pangolin and TraP, with weighted F1 scores decreasing by 0.303 and 0.757, respectively (Supplementary Table S2, Figure 1C). Conservation scores and whole-genome predictors performed poorly as well. Except for CAPICE and CADD-Splice, most methods displayed weighted F1 scores below 0.15 at the 11-40 bin (Supplementary Table S2). Overall, the most performant tools were CI-SpliceAI, Pangolin and SpliceAI with average weighted F1 scores across all intronic bins of 0.672, 0.661 and 0.627, respectively (Figure 1C).

Strikingly, we noticed an increase in performance in the deepest intronic bins when compared to intermediate distances (Figure 1C). We hypothesized that variability in transcript structures could be the reason: despite these variants being assigned as occurring very deep within introns (> 500bp from the splice site) according to the associated RefSeq transcript, they may be exonic or near-splice site variants in other isoforms of the associated gene. To tackle this question, we looked at the raw transcript annotations (without picking Ensembl Variant Effect Predictor (VEP) consequences) of the variants assigned to the > 500bp bin (N=1501) and decomposed them into several sub-categories based on their localization in different transcript isoforms (see Methods). Our analysis revealed that 304 variants are located in exons in other transcripts and 274 variants mapped to introns but closer to splice sites than in the transcript isoform originally considered (Supplementary Figure S1B). In particular, some of the intronic variants are located at splice sites in other transcripts (Supplementary Figure S1C). We found that the performance of the tools was generally better for these categories than for categories where the variant distance to the splice site remained unchanged (Supplementary Figure S1D), which is consistent with the hypothesis that deep intronic pathogenic variants are hard to predict. After excluding variants from exonic and closer-to-splice sites categories, we repeated the per-bin analysis to see whether the performance increase in the deepest bins remained. We observed that most conservation-based methods and whole genome predictors displayed a decline in performance compared to the original analysis (Figure 1D). On the other hand, a subset of splicing tools such as ConSplicemL, SpliceAI, Pangolin or CI-SpliceAI showed better performance than before, suggesting that unequivocal deep intronic variants in ClinVar are associated with splicing and that SpliceAI-based methods can identify them reasonably well.

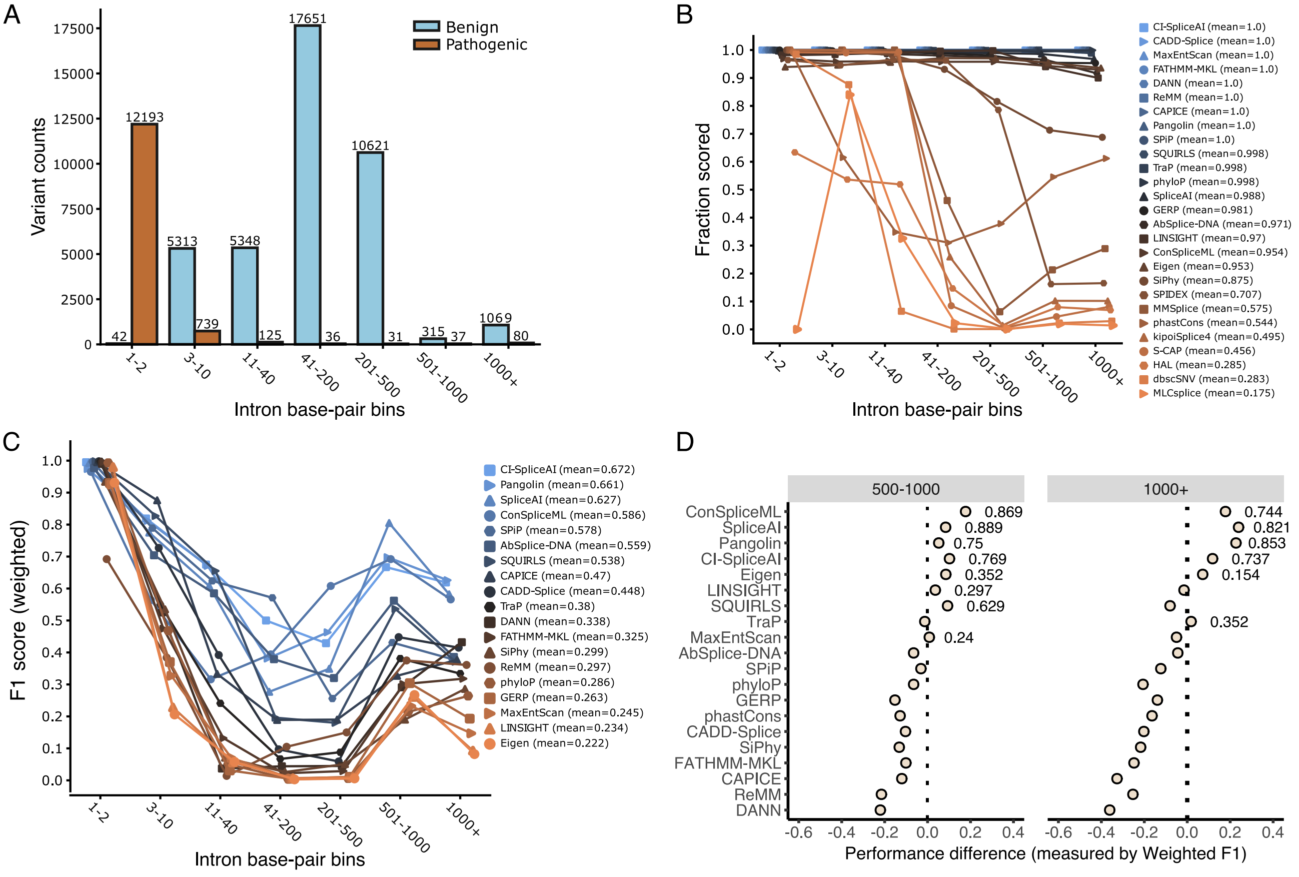

Figure 1. Intronic variant prediction in ClinVar. **A** - Distribution of variants across each intronic bin considering the RefSeq transcript associated with each ClinVar variant. **B** - Fraction of variants scored (with predictions) at each intronic bin. Mean values in the legend represent the average fraction of variants scored across all bins. **C** - Performance of tools that predict entire introns (defined as > 90% scored variants) at each intronic bin. Mean values in the legend represent the average weighted F1 score across all bins. **D** - Differences in performance per deep intronic bins ("501-1000" and "1000+") after removing variants that are exonic or closer to splice sites in other transcripts of the associated gene. Points refer to the weighted F1 difference between this new analysis minus the values obtained originally (displayed in C)). Annotations next to points refer to the weighted F1 scores in the new analysis for the tools whose performance difference is positive.

Pathogenic splicing-affecting variants are captured well by deep learning based methods

Not all ClinVar intronic variants are associated with splicing defects. However, splicing-related tools were the most successful at predicting the pathogenicity of deep intronic mutations. Therefore, we decided to narrow our focus to variants that specifically affected splicing. We previously published a dataset of deep intronic variants causing human disease via disruption of splicing (N=81) [20]. In the current study, we augment the dataset by performing a comprehensive literature search for case reports published after 2017, where the association between a variant and a splicing defect was supported by experimental evidence, such as from RT-PCR, sequencing of cDNA products, RNA-Seq or minigene/midigene assays (Supplementary Table S3). This new curation effort is composed of 161 variants covering a diverse range of disease phenotypes, with most diseases represented by fewer than 3 variants (Supplementary Figure S2A). A great number of these variants are not yet reported in ClinVar (N=90), and of those that are reported, a few (N=11) are incorrectly classified as VUS, with review status ranging from 0-1 stars (Supplementary Figure S2B). As further evidence of their pathogenicity, the variants are very rare in the general population, as most of them are absent from gnomAD [2], a widely used catalog of genetic variation across human populations (Supplementary Figure S2B, C).

The results showed that SpliceAI-derived methods outperformed the remaining tools. PDIVAS displayed the highest area under the ROC (auROC), followed by Pangolin, ConSpliceML and SpliceAI (Figure 2A). However, evaluation using single thresholds revealed lower performance than using auROC, which is based on multiple thresholds (Supplementary Figure S2D). As practical clinical applications usually require a binary decision, this prompted us to optimize reference thresholds for detecting splice-affecting pathogenic intronic variation outside of the canonical splice site regions (see Methods). After threshold recalibration, we reveal SpliceAI and Pangolin as the best tools (weighted normalized MCC > 0.92) to identify pathogenic variants using a single cutoff value (Figure 2B, Supplementary Table S4). As a practical outcome of this analysis, we provide recalibrated thresholds for different trade-offs between precision and recall (Supplementary Table S5).

When available, we recorded information on the molecular consequences of each variant on splicing. Pseudoexon activation was the most frequent consequence of deep intronic variants (194 out of 242 in our dataset). We also identified 37 variants leading to partial intron retention due to the usage of an alternative splice site. Exon skipping was observed in only 6 cases, consistent with previous observations that functional deep intronic variants are less commonly linked to this mechanism [84]. We next compared the tools' ability to detect pseudoexon activation and partial intron retention variants using the optimized thresholds. We hypothesized that the tools would perform better on the partial intron retention group since these variants are located closer to the splice sites than those that activate pseudoexons (Supplementary Figure S2E). Nonetheless, we observed no statistically significant differences between the two groups, with SpliceAI-derived methods performing slightly better in the pseudoexon activation group (Figure 2C).

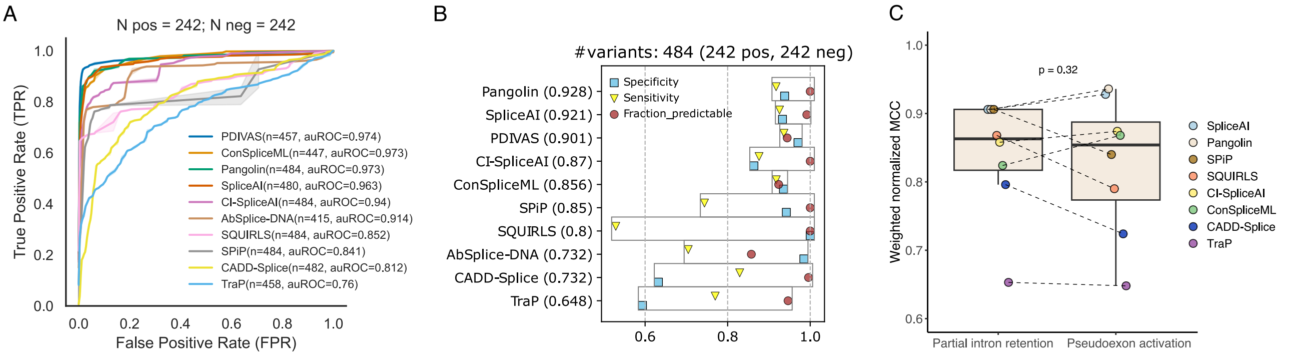

Figure 2. Pathogenic variant prediction of deep intronic variants affecting RNA splicing (81 variants from Vaz-Drago et al. [20] and 161 curated for this manuscript). **A** - Receiving Operating Characteristic (ROC) analysis for all splicing-associated methods. **B** - Performance using optimized thresholds for intronic variation outside of canonical splice regions. The weighted normalized MCC was used to rank the tools. **C** - Performance using optimized thresholds on two different subsets of variants: variants leading to partial intron retention and variants leading to pseudoexon activation. PDIVAS and AbSplice-DNA were excluded as they do not score the two groups equally (PDIVAS only predicts variants located 50bp beyond the nearest splice site, while AbSplice-DNA scores variants within 100bp of any splice junction observed in GTEx data). Wilcoxon Signed Rank test, performed as a one-sided test, was used to compare the values between the two groups of variants.

Performance varies considerably when predicting splicing-altering variants associated with different molecular mechanisms

To gain further insight into the molecular mechanisms driving the splicing alterations, we generated datasets of alternative splicing events triggered by intronic variants occurring at different regions that are important for splicing regulation (Table 2). We defined six categories (Figure 3A, see Methods). Within each region, we separately evaluated variants that trigger partial intron retention (via alternative splice site usage at annotated exons) and variants that lead to pseudoexon activation. Importantly, contrary to the analyses performed above, we evaluate performance not based on the ability to distinguish between pathogenic and non-pathogenic variants but rather between variants that do (positive class) or do not (negative class) affect the mechanistic splicing outcome. This is relevant as a variant may, e.g., create a pseudoexon, thus affecting the outcome of splicing, without necessarily leading to disease. This decision was made as information on variant pathogenicity was not always available. We also switch to reporting performance using the area under the PR curve (auPRC), which is a metric to summarize precision-recall curve analysis. This decision was motivated by two factors. Firstly, some categories have unbalanced data, with fewer positive instances compared to negatives. The auPRC score provides a more nuanced evaluation of tool performance by focusing on the accurate identification of positive instances. Furthermore, it eliminates the need for a single universal cutoff, accommodating the fact that different categories may have distinct optimal thresholds.

Branchpoint associated variants

Branchpoint associated variants were defined as located 18 to 44 bp upstream of the splice acceptor of a cryptic or canonical splice site, either leading to pseudoexon activation, partial/full intron retention, or exon skipping (Supplementary Table S6). In addition, we confirmed that the variant either disrupted or created any of the four (increasingly relaxed) BP motifs described in [77]: YTNAY, YTNA, TNA, YNA. Particularly, we excluded any splicing-altering variants located 1 bp upstream of the branchpoint adenine. The final positive branchpoint-associated dataset (N=82) spans 7 different sources, with Leman et al. [33] contributing the most (N=31, Table 2). The negative variants are located 18 to 44 bp upstream of an annotated splice site and had been shown not to affect splicing using the minigene-based reporter assays Vex-seq [86] and MFASS [87](Table 2, Supplementary Table S6). Because BP variants activating pseudoexons were scarce (N=4) and the molecular consequences of BP-associated variants are not clear-cut (e.g., the same BP variant may lead to intron retention and to exon skipping), we analyzed all the variants affecting the BP motif together. For this analysis, we additionally included four branchpoint prediction tools: SVM-BPFinder, BPP, LaBranchoR [76] and BPHunter [77]. Moreover, we included IntSplice2 [73] since it predicts splicing-associated Single Nucleotide Variants (SNVs) at intronic positions overlapping the branchpoint region.

Pangolin was the best-performing method for BP-associated variant prediction with an impressive auPRC score of 0.93 (Figure 3B, Supplementary Figure S3A). This result suggests that the training of Pangolin on multi-species data potentially contributed to increased robustness in capturing the complexity of the branchpoint code. Among the tools specifically designed to predict BPs, LabRanchoR and BPHunter were very competent, ranking 2nd and 4th, respectively, with auPRC scores of 0.877 and 0.87 (Supplementary Table S7). Conversely, BPP and SVM-BPFinder displayed more modest results.

Acceptor Upstream and New Splice Acceptor variants

The *Acceptor Upstream* category refers to splicing-altering variants that mostly locate upstream (up to 18bp) of an existing cryptic splice acceptor and activate it. On the other hand, the *New Splice Acceptor* category contains variants that form new splice sites themselves (Figure 3A). We collected negative variants differently for each of the two categories. For *Acceptor Upstream* variants, we extracted variants located upstream of annotated splicing acceptors that did not interfere with the splicing outcome, as demonstrated through MFASS, Vex-seq or Moles-Fernández et al. [35]). The BP region from 18 to 44 bp was excluded. Conversely, we assigned common (>5% allele frequency) deep intronic gnomAD variants that create new splice acceptor motifs as negative *New Splice Acceptor* variants (Table 2, see also Methods). Despite creating a splice acceptor motif, these variants are considered non-functional due to their high prevalence in the general population. While it is theoretically possible that these variants do affect splicing (e.g, if they occur in non-essential genes where splicing alterations have little fitness effect), we confirmed that their genomic locations were not used as splice junctions in individuals from the GTEx [65] cohort.

The sets of splicing-altering variants we collected for each category were similar in size (71 and 64 variants for acceptor-upstream and new splice acceptor, respectively). However, when we split the variants according to the major molecular group (pseudoexon inclusion vs. partial intron retention), we obtained a very small number of new splice acceptor variants in the partial intron retention group (N=13), hence rendering their computational evaluation statistically limited. Therefore, for this particular analysis, we merged *Acceptor upstream* and *New Splice Acceptor* variants into a new *Acceptor associated* class so that we could have a reasonably large dataset to

Table 2. Sources of data used to build region-specific splicing datasets.

Study	Variants*	Per category**	Description
Splicing-altering			
Vaz-Drago et al. [20]	81	AU=3;NSA=12;EL=10; NSD=39;DD=17	Manual curation of disease-causing deep intronic variants with experimental validations
Our curation	140	BP=7;AU=11;NSA=26; EL=19;NSD=43;DD=34	Manual curation of disease-causing deep intronic variants with experimental validations
Keegan et al. [21]	143	BP=1;AU=12;NSA=21; EL=13;NSD=71;DD=25	Characterization of hundreds of mutation events driving cryptic splicing via pseudoexon activation
Petersen et al. [85]	10	BP=1;AU=1;EL=7 ;DD=1	Characterization of pseudoexons activated by deep intronic mutations that do not create or strengthen cryptic splice sites
Tubeuf et al. [34]	3	EL=3	Benchmark of user-friendly tools for predicting variants affecting splicing regulatory elements
Jung et al. [84]	231	BP=25;AU=42;NSA=3; EL=56;NSD=37;DD=68	Identification of intronic mis-splicing mutations from RNA-Seq using a read ratios approach
Moles-Fernández et al. [35]	15	BP=1;AU=2;NSA=2; EL=2;NSD=7;DD=1	Benchmark of using both SpliceAI and user-friendly tools to identify deep intronic variants that disrupt splicing
Leman et al. [33]	31	BP=31	Benchmark of bioinformatics tools to predict BP as well as the impact of splicing variants occurring in the BP area
Zhang et al. [77]	16	BP=16	Genome-wide analysis of human branchpoints and development of a tool to score BP-associated variants
Splicing-neutral			
Moles-Fernández et al. [35]	98	BP=2;AU=35;DD=61	Benchmark of using both SpliceAI and user-friendly tools to identify deep intronic variants that disrupt splicing
Vex-seq [86]	277***	BP=59;AU=52, EL=119;DD=47	Vex-seq, a MPRA to test the impact of 2059 variants in splicing across 110 alternative exons
MFASS [87]	109	BP=34; AU=17; DD=58	Multiplexed functional assay (MFASS) that assayed the splicing effect of 27,733 ExAC variants
gnomAD [2]	261	NSA=64; NSD=197	Common (and hypothetically benign) variants that create true splice site motifs

* Total number of variants used from original study. Since several variants were duplicated across studies, we kept unique occurrences given the order they appear in the table (top to bottom).
** Number of variants contributing to each category. BP = Branchpoint-associated; NSA = New splice acceptor; NSD = New splice donor; AU = Acceptor upstream; DD = Donor downstream; EL = Exonic-like. Note: we could not assign a category to all variants of our curation, hence the lower number as compared to the original dataset (N=161).
*** Exceptionally, 119 variants from this study are exonic.

evaluate (Supplementary Table S6). As for the *Branchpoint associated* variants, we added IntSplice2 to the list of tools to evaluate. In addition, we included two splice site prediction methods that we customized to predict variant effects in VCF format: SpliceRover and Spliceator.

SpliceAI, PDIVAS, Pangolin, ConSpliceML and CI-SpliceAI achieved good performance on pseudoexon-activating variants, with auPRC above 0.9 (Figure 3B). However, when it comes to variants causing partial intron retention, performance drops considerably, with no tool achieving an auPRC score higher than 0.85 (Figure 3B). Except for PDIVAS, which had a substantial amount of missing data for this analysis, the top tools remained unchanged, with Pangolin, SpliceAI and CI-SpliceAI displaying AP scores of 0.847, 0.816 and 0.765, respectively (Supplementary Figure S3C, Supplementary Table S7). Among the tools specifically added for this analysis, SpliceRover was the most competitive, ranking 6th in the pseudoexon group and 5th for partial intron retention variants (Supplementary Figure S3B, C).

Exonic-like variants

We consider here intronic variants that lie within either an activated pseudoexon or within an annotated exon that undergoes alternative splice site usage (Figure 3A). We identified 110 splicing-altering variants to compare against 119 splicing-neutral exonic variants from Vex-seq (Supplementary Table S6). After grouping the variants according to the major group, we obtained 78 pseudoexon-activating variants vs. 32 variants triggering partial intron retention. Accordingly, we randomly split the negative the variants between the two groups so that the final datasets were fairly balanced (84 and 35 variants for each group, respectively). For this comparison, we also included three approaches that quantify splicing regulatory elements that enhance or repress flanking splice sites: ESREseq scores, HEXplorer and ESEfinder.

Once again, we observed better overall performance for the pseudoexon group compared to the partial intron retention group (Figure 3B, Supplementary Figure S3D, E). Pangolin and SpliceAI were among the best tools in both major groups. Interestingly, HEXplorer and ESREseq performed better for the pseudoexon group than models that incorporate deep learning based predictions such as AbSplice-DNA or ConSpliceML (Supplementary Figure S3D, Supplementary Table S7).

Although SpliceAI performed best comparing to other methods, its pre-computed scores were configured to only report variant effects in a 50-bp window from the variant site. While this window is fine for most variant types (the affected splice sites are usually close to the variant site), that may not be the case for pseudoexon-activating variants that could be located deep inside the pseudoexon (assuming a pseudoexon of the size of an annotated exon). Therefore, we selected the splicing-altering variants missed by SpliceAI using the optimized threshold of 0.05 (N=25) and used the SpliceAI Lookup API [88] (last accessed May 25th, 2023), to run the model using a larger maximum distance (500 bp). We observed that 9 out of 25 were correctly reclassified as splicing-altering (Supplementary Table S8), suggesting that SpliceAI performance may be underestimated when ignoring longer-range variant effects.

New Splice Donor variants

We identified 197 positive variants falling into this category (Table 2). For the negative set, we used variants that created a GT dinucleotide resulting in a splice donor consensus (GGTAAG), but that were unlikely to act as a cryptic splice site as they appeared in gnomAD with a population frequency >5% and were not observed to be used as a splice junction in GTEx individuals. We added SpliceRover, DSSP and Spliceator tools to the evaluation.

PDIVAS demonstrated the best performance in the pseudoexon activation group, achieving an auPRC score of 0.981. On the other hand, AbSplice-DNA outperformed other tools for partial intron retention variants with a performance metric of 0.94 (Supplementary Figure S3F, G). Similarly, SpliceAI, ConSpliceML, Pangolin and CI-SpliceAI exhibited excellent results (Figure 3B), indicating that

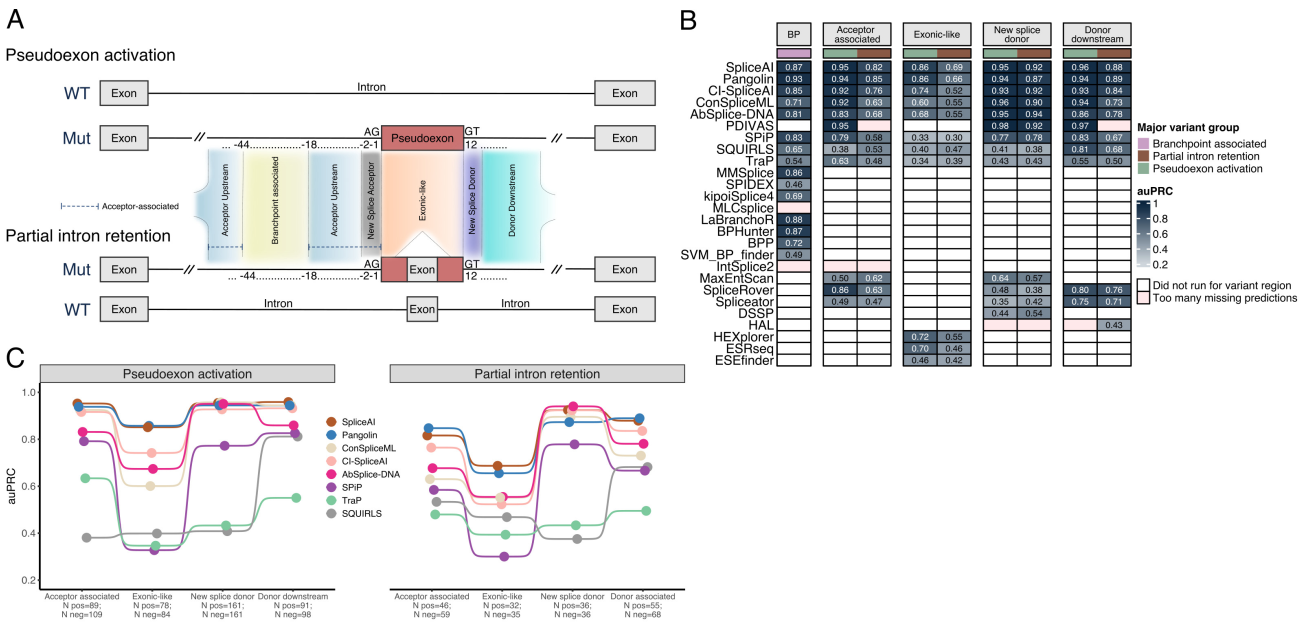

Figure 3. Tool performance evaluation on multiple regions associated with the regulation of splicing. **A** - Schematic representation of the regions used to define each dataset. For each of the two major groups (pseudoexon activation and partial intron retention), we show the expected wildtype (WT) structure in the absence of the variant, as well as the abnormal structure caused by the variant (Mut). Red blocks represent regions of the mRNA that are incorrectly spliced in. Exceptionally, some branchpoint-associated variants result in exon skipping, which is not graphically represented in the figure. **B** - Global overview of the performance (quantified with auPRC) for all the datasets analyzed. **C** - auPRC scores for the tools that can predict entire introns.

these models are very well-suited for predicting this category of variants. Importantly, we noticed a large performance gap between SpliceAI-related tools (plus SPiP) and the rest, which performed rather poorly (almost all tools with AP scores below 0.6, Figure 3B, Supplementary Figure S3F, G). Considering that splicing-negative variants in this dataset create hypothetical splice donor decoys, we wondered whether tools that incorporate cryptic splice site scoring features using short sequence windows surrounding the variant site (Position Specific Scoring Matrix (PSSM)-based for TraP, information content-based for SQUIRLS) would predict negative variants as splicing-altering. Indeed, we observed a large proportion of false positives for these tools in the pseudoexon-activation group when using a single reference threshold for evaluation (1.0 for TraP and 0.98 for SQUIRLS, Supplementary Table S7). Conversely, deep learning based methods such as SpliceRover, DSSP and Spliceator may rely too much on the near-splice site features (despite using larger sequence contexts), hence the poor performance observed.

Donor Downstream variants

This category refers to all splicing-altering intronic variants located downstream of the cryptic splice donor event (N=146). Negative variants (N=166) are located downstream of annotated exons and were shown experimentally to have no impact on splicing outcomes (Table 2, Supplementary Table S6). As before, we included SpliceRover and Spliceator in the analysis. DSSP was excluded since it predicts splice sites at fixed positions in the input, but in this category, variant positions with respect to the cryptic splice donor are variable.

PDIVAS and SpliceAI excelled on the subset of variants triggering pseudoexon activation with auPRC scores of 0.969 and 0.959, followed by ConSpliceML, Pangolin and CI-SpliceAI, all with performance values above 0.9 (Figure 3B, Supplementary Figure S3H, Supplementary Table S7). Regarding the partial intron retention subset, Pangolin and SpliceAI performed the best (auPRC scores of 0.89 and 0.879), with a larger difference for the tool ranked third, CI-SpliceAI (AP=0.836, Supplementary Figure S3I). Again, these results demonstrate the superiority of SpliceAI-derived approaches versus standard methods that engineer domain-specific features to score intronic splicing variation.

All regions combined

Next, we combined all the datasets to inspect the global performance of each major variant group. Eight methods were able to score all types of splicing variants in any intronic region. These tools were SpliceAI, Pangolin, ConSpliceML, CI- SpliceAI, AbSplice-DNA, SPiP, TraP, and SQUIRLS (Figure 3C). Except for AbSplice-DNA, which scores intronic variants located up to 100bp away from splice junctions used in any GTEx tissue, all the methods were designed to score any given position in introns.

SpliceAI and Pangolin consistently ranked highly for all datasets (Figure 3C). CI-SpliceAI, AbSplice-DNA and ConSpliceML were fair alternatives, especially for variants that create new splice donors. SPiP was particularly inadequate for exonic-like variants, but was the best non-deep learning-based method for the remaining categories (Figure 3C).

Overall, we observed a trend of pseudoexon-activating variants being predicted more accurately than partial intron retention variants (Figure 3C, Supplementary Figure S4A). However, when evaluating each tool individually, this trend did not reach statistical significance for the majority of them (Supplementary Figure S4B).

Assessing interpretability

We were interested in the extent to which these state-of-the-art tools give additional information to the user, besides the prediction. Among the tools that predict across whole introns, SQUIRLS and SPiP are the only ones intentionally designed to provide some interpretation of the outcome. SQUIRLS can generate HTML reports with short descriptions of why the model predicts pathogenicity and displays the contribution of each feature to the outcome. In addition, it draws figures to show the variant effect in the sequence context surrounding the variant. SPiP provides short interpretation tags describing the molecular consequences of the variants along with confidence intervals for the probability that the variant impacts splicing. Recently, a novel strategy was introduced to aid in the interpretation of splicing-associated variants, leveraging RNA-Seq data from more than 300,000 individuals [89]. This approach, SpliceVault, focuses on quantifying the relative prevalence

of stochastic and unannotated splicing events in population-based RNA-seq data, enabling the prediction of the nature of mis-splicing induced by a variant. Given its innovative approach and the ability to provide interpretations for variant consequences, we included SpliceVault in our assessment.

We devised a procedure to evaluate how accurate the interpretations are against the biological ground truth (see Methods). We used the splicing-associated deep intronic pathogenic dataset analyzed before (Figure 2) and specifically selected variants with complete annotations, including molecular effect and functional consequence (N=221) for assessing interpretation quality. SPiP and SQUIRLS correctly predicted 170 and 121 variants, respectively, and those were selected for downstream analysis. In contrast, SpliceVault does not predict variant effects directly. Instead, it checks the mis-splicing occurring in the surroundings of an annotated exon of interest. As a result, we did not include pseudoexon-activating variants because SpliceVault cannot provide information about such an outcome (despite potentially identifying one of the two splice junctions of the pseudoexon). This left us with 37 variants for analysis (Supplementary Table S9). Our evaluation revealed that SQUIRLS, SPiP, and SpliceVault were able to provide correct interpretations (within the limitations of each approach) for a considerable fraction of the variants. However, for many others, no interpretation could be found. Specifically, SPiP lacked interpretations for 46 variants, SpliceVault for 22 variants, and SQUIRLS for 21 variants (Figure 4A). In the case of SpliceVault, this accounted for more than half of the analyzed variants (22 out of 37). Further inspection of these results showed that most of these variants create a core splice site dinucleotide (Supplementary Figure S5A), and this type of mis-splicing event is not appropriate to be captured by SpliceVault. Regarding SQUIRLS and SPiP, and looking at the prediction score distribution for each category, we observed that the variants with no interpretation have the lowest scores (Supplementary Figure S5B). On the other hand, correct explanations are spread across the full score range. Interestingly, SPiP explanations that are not informative (events with no association with any splicing mechanism) have the highest median score range, showing that strong effects are not necessarily easier to explain.

Predicting splicing changes across tissues

Of the tools evaluated in this study, Pangolin and AbSplice-DNA can both predict splicing outcomes in a tissue-specific fashion. We decided to use AbSplice-DNA alone for this analysis. Pangolin was trained on sequences and splice site usage levels from four tissues across four species (human, rhesus macaque, mouse and rat). However, the default settings of Pangolin are tissue-agnostic and it requires additional customizations to get tissue-specific variant effect predictions. On the other hand, AbSplice-DNA provides pre-computed tissue-specific predictions. Moreover, it combines tissue-specific splicing annotations created from GTEx data with DNA-based prediction models, enabling it to predict variant effects in more tissues (49). We aimed to evaluate whether AbSplice-DNA predictions of disease-causing variants are enriched for the tissues that are most strongly affected by the disease.

Using the splicing-associated variant dataset described above (N=242, Figure 2), we determined, when possible, the GTEx tissue most closely associated with the given disease based on the HPO [90] (see Methods). We selected the 155 variants that AbSplice-DNA predicted correctly, which excluded all the variants causing two of the most common diseases in our dataset: Becker muscular dystrophy and Duchenne muscular dystrophy (Supplementary Table S10). In addition, 35 variants were not evaluated since they were not assigned to any particular tissue (e.g. systemic diseases, or diseases affecting tissues not represented in GTEx, such as the retina), leaving 120 variants to analyze. Considering disease variants associated with only one GTEx tissue, we observed enrichment of the expected tissues to a limited extent (Figure 4B, Supplementary Figure S6A). For example, Hypertrophic Cardiomyopathy variants were highly enriched in the heart tissue, an Ataxia with Oculimotor Apraxia variant was predicted to affect the cerebellum and a Congenital hypothyroidism variant was enriched for thyroid. Interestingly, variants associated with blood disorders (Factor VII deficiency and Afibrinogenemia) have the highest prediction scores in the liver, which is not unexpected, since the liver plays a crucial role in the production of clotting factors, including factor VII and fibrinogen (Figure 4B). However, other tissue-specific predictions had unclear interpretations, such as the enrichment of testis for several diseases, the brain cerebellum in Adenomatous Polyposis (associated with colon and rectum, Figure 4B), or the skeletal muscle in Fabry disease (primarily linked to other tissues such as heart and kidneys, Supplementary Figure S6A). In addition, 40 variants displayed the same score across all tissues, which does not reflect the expected biology, especially for some diseases associated with a single tissue (Supplementary Figure S6B).

Discussion

We have performed a comprehensive benchmark study of intronic variant prediction, focusing on disease-causing deep intronic variants affecting splicing via pseudoexon inclusion or partial intron retention. Furthermore, we collected and examined variant sets based on their location relative to the splice sites affected by the altered splicing. Finally, we assessed tool interpretability and provide some considerations on the use of computational models beyond the prediction score.

We used two different datasets to study intronic variants causing human disease. ClinVar is a database that has been widely used for this purpose. Nevertheless, to the best of our knowledge, it has not been used to evaluate performance as a function of distance to the splice sites. Averaging performance across all bins, we found that splicing-associated tools performed the best overall on ClinVar data. Importantly, we observed a decrease in performance immediately after the two splice site positions, with a particularly noticeable decline at a distance of 11 base pairs from the closest splice site. These results demonstrate the extent to which these methods are biased to predict splice site variants, whereas smaller effect-size variants deeper inside the intron go mostly unnoticed. For many of the tools, such as S-CAP or MLCsplice, this is not unexpected, as they were not designed to predict variants in deep intronic regions. In addition, we observed that some of the variants that appear deep-intronic in the clinically-relevant transcript are exonic or located close to the splice sites in other isoforms of the associated gene. Therefore, and according to the American College of Medical Genetics and Genomics and the Association for Molecular Pathology (ACMG-AMP) guidelines [91], we recommend considering multiple isoforms when interpreting deep intronic variants, especially when the canonical isoform is not highly expressed in the tissue of interest [92].

Additionally, we curated a diverse set of pathogenic deep intronic mutations that exclusively affect splicing. Tools that predict across all intronic regions, notably SpliceAI-derived models, showed satisfactory performance. Many variants in this dataset generate new splice sites deep within introns, activating pseudoexons. We speculate that sequence-based models that predict splice sites are particularly well suited to predicting this class of variants, likely because the pseudoexons resemble the sequence context of authentic exons [85] that were presented during their training.

To better understand performance differences between classes of variants, we collected a diverse set of experimentally tested splicing-associated variants, and evaluated the tools' ability to distinguish them from similar non-splice-altering variants. Region-specific analysis revealed substantial differences in performance. In agreement with previous studies [35, 93], we found that variants

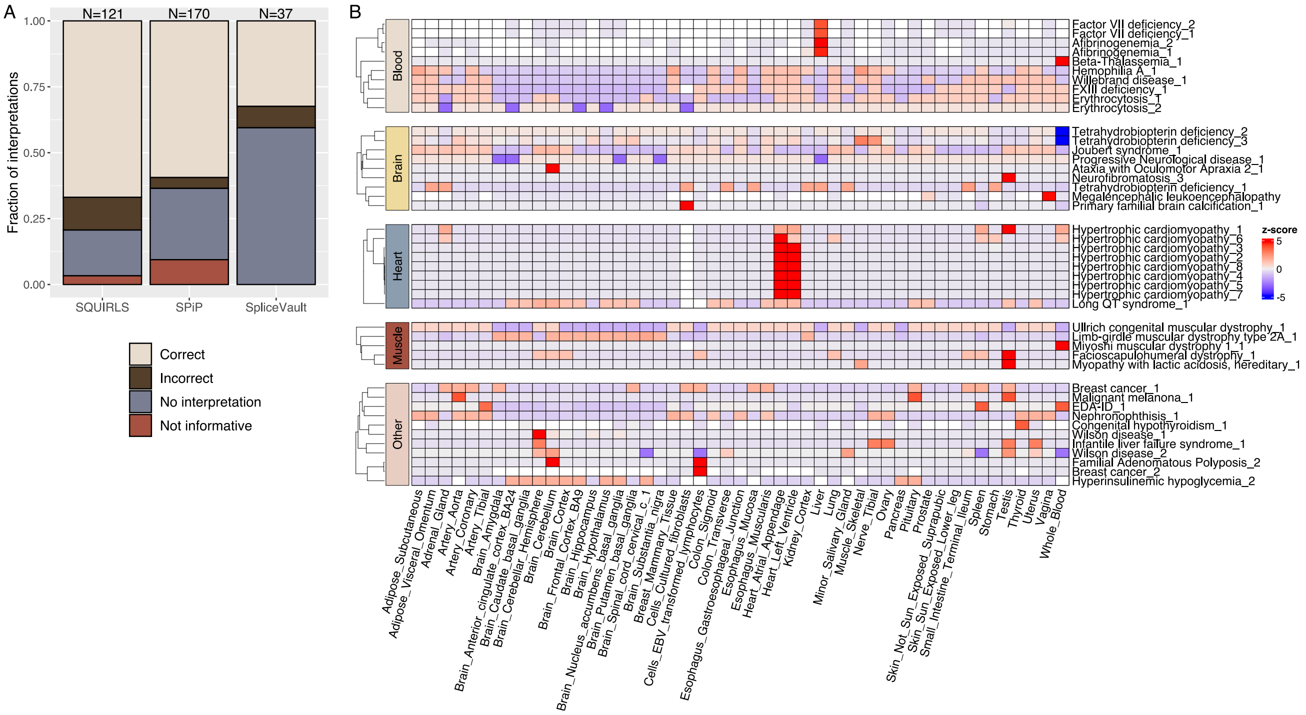

Figure 4. Information provided by the tools beyond the prediction score. **A** - Assessing the quality of the interpretations for SQUIRLS, SPiP and SpliceVault. Within each bar, the height of each category represents the fraction of variants assigned to the given interpretation quality tag. The numbers above the bars indicate how many pathogenic variants were used. **B** - Tissue-specific predictions made by AbSplice-DNA for a set of disease-causing variants associated with a single tissue, according to Human Phenotype Ontology (HPO). Phenotype names are displayed in rows, GTEx tissues predicted by AbSplice-DNA are in columns. High z-scores represent tissues for which the variant effect is stronger compared to other tissues.

affecting putative exonic splicing regulatory elements were among the hardest to predict. The binding motifs of many splicing factors are highly degenerate or even unknown, and their impact on splicing largely depends on the cell type [94, 95]. Nevertheless, such complexity appears to be better captured by SpliceAI and Pangolin than by tools with built-in domain knowledge.

The recent progress achieved through deep learning models that work as black boxes has raised concerns about their deployment in sensitive domains such as healthcare [96]. Because practitioners are interested in understanding how these AI systems make decisions, we assessed the capacity of these models to provide interpretable outputs when predicting disease-causing variation associated with splicing defects. Although most sequence-based models, such as SpliceAI, provide some information beyond the prediction score, namely the distance of the variant to the affected mRNA position, it is only possible to obtain insight into the inner workings of the model by applying external explainability techniques [97]. On the other hand, SQUIRLS and SPiP are intrinsically more interpretable by design. The models were frequently able to correctly identify the type of splicing alteration. However, these models suffer from an accuracy-interpretability trade-off since the performance across evaluations was lower than that of black box models. The recently published SpiceVault portal also provides an accurate interpretation of the nature of mis-splicing defects, however, it does have limitations that are particularly pronounced when dealing with variants deep in the introns. Particularly, it cannot properly analyze pseudoexon activation events or cryptic splicing caused by variants that create new splice sites at the core dinucleotide motif. Note that to our knowledge, no tool exists that can provide higher-order mechanistic interpretations, such as identifying the particular splicing factors or regulatory motifs involved.

Another promising research avenue is the prediction of splicing abnormalities in a tissue of interest, which AbSplice-DNA offers. The model could accurately detect some tissue-specific differences relevant to human disease, yet it was unreliable for the majority of variants. Nonetheless, we acknowledge that the introduction of SpliceMaps [27], which provides information on splice site usage across GTEx tissues, combined with RNA-Sequencing of clinically accessible tissues (CATs), is expected to enhance the prediction of functional intronic variants [27], particularly in diseases where the splicing landscape of the relevant non-accessible tissue is appropriately represented by one of the CATs [98].

Practical recommendations

We advocate using deep learning based solutions to obtain maximally accurate predictions. SpliceAI and Pangolin consistently ranked high for intronic variants associated with splicing, both for the prediction of pathogenicity and of altered splicing. We determined optimal thresholds for deep intronic regions (SpliceAI=0.05, Pangolin=0.053) for clinical purposes. However, it is important to note that despite the diversity of genes, phenotypes and molecular mechanisms covered in our dataset, users should be mindful that optimal thresholds can vary depending on the variant class or affected exon [99].

SpliceAI and Pangolin are usually run programmatically on the command line. However, if usability is a primary concern and users have a limited number of predictions to make, the Broad Institute offers a convenient web application. The web application [88] incorporates both SpliceAI and Pangolin. For SpliceAI, it not only provides the conventional delta score (mutated - reference) but also presents the raw splice site probability predicted by the model. This can be particularly useful for certain situations. For instance, when a splice site is already predicted with a high score in the reference sequence (e.g., 0.85), the delta score for a splice-promoting mutation can only be low (no more than 0.15, in this example). This is because SpliceAI scores are capped at 1. This context is important for the correct interpretation of the delta scores. With this in mind, it is also worth considering SpliceAI-visual [100], available at [101]. SpliceAI-visual handles complex variant types, and employs

raw SpliceAI scores to generate graphical outputs that are easier to interpret. If the number of variants makes it unfeasible to use these web applications, but the user does not have the computational know-how to work on the command line, CI-SpliceAI is a good alternative, since its online service [102] allows the input of multiple variants in a VCF-like format. Practitioners, however, may suspect that splicing is not the mechanism disrupted by a particular mutation. In this scenario, we recommend using CAPICE since it was the best whole genome predictor on ClinVar data, although with very limited performance.

Region-specific splicing benchmarks revealed additional insights for tool usage. We recommend using Pangolin to prioritize variants in branchpoint regions (-18 to -44 bp upstream of splice acceptor). LabRanchoR and BPHunter were the best branchpoint-specific tools in our evaluation and can also be considered. SpliceAI and Pangolin were the most effective at scoring acceptor-associated variants (splice acceptor creating or polypyrimidine tract variants upstream of cryptic splice acceptors). Including other sequence-based deep learning models that use smaller sequence contexts did not provide additional value. Intronic variants affecting splicing regulatory elements within cryptic exons are hard to predict. We endorse using SpliceAI with larger windows surrounding the variant site (setting the distance parameter to the maximum). In addition, classical approaches such as HEXplorer might come in handy for specific cases, such as assessing the potential impact of a variant on exon-defining regulatory motifs. Finally, SpliceAI-inspired models (Pangolin, CI-SpliceAI) and models that incorporate SpliceAI predictions as features (ConSpliceML, AbSplice-DNA, PDIVAS) can effectively predict new splice donor and donor-downstream variants. However, to keep the number of different tools to use to a minimum, we suggest using the original SpliceAI model.

Nonetheless, it is noteworthy to mention the impacts of using pre-computed scores as the strategy for variant prioritization. The current version of SpliceAI pre-computed scores (v1.3.1) does not include predictions for insertions and deletions larger than 1 and 4 nucleotides, respectively. In addition, the limit of 50 bp as the distance around the variant site to extract variant effects prevents SpliceAI from identifying other variant classes, such as exon skipping, when the variant exerts its effects at more than 50 base pairs from the affected exon.

Finally, when interpretable outcomes are important the choice of the strategy may depend on the use case. There is currently no option covering all possible mis-splicing scenarios, and each method assesses interpretability differently. SpliceVault is a recently published web application that is effective when interpreting intronic variants leading to exon (or multi-exon) skipping, or partial intron retention through activation of pre-existing cryptic splice sites. Alternatively, SQUIRLS can be applied, as the software is well-designed, thoroughly documented and generates HTML reports that practitioners can intuitively inspect. Nonetheless, it does not handle pseudoexon activation consequences properly (for that, SPiP is recommended). In addition, it should not be solely relied upon as a prediction tool, as it is not as performant as other models.

Final remarks

We comprehensively assessed functional intronic variation occurring far from annotated splice sites. As a result, we make available to the community region-specific datasets that can be used to evaluate new models on variants whose molecular consequence is known. These datasets will assist developers in identifying potential limitations of the model and highlighting variant types that it is more prone to fail on. Additionally, we encourage developers to make their models publicly available by sharing them on open-source platforms to facilitate their reuse [64, 103].

Sequence-based models based on Convolution Neural Networks architectures are still the state-of-the-art approach for splicing variant prediction. However, the artificial intelligence field is rapidly evolving, and we have seen the emergence of Transformer-based architectures being applied to other variant effect prediction tasks, e.g., effects on gene expression [104] or on protein function using large protein language models [105]. As a result, increasingly complex models are expected to effectively tackle open questions in splicing regulation, such as better capturing the synergistic effects of splicing regulatory elements. However, the community must be aware of the possible implications these models bring, such as a lack of transparency and decreased ability to generate mechanistic hypotheses.

Methods

Data collection and variant annotation

We employed the same variant annotation procedure for all the variants collected for this manuscript (datasets described below). We used Ensembl VEP v109 ([106]) for the task and transcript annotations were added accordingly (with '–per_gene –pick_order ccds,canonical,biotype,rank –no_intergenic –gencode_basic' set). We used variants in the GRCh37 genome build simply because several of the tools we include in the manuscript do not support the GRCh38 genome build. Nonetheless, we provide all the datasets and predictions in both GRCh37 and GRCh38 (via liftOver) versions.

ClinVar

We downloaded ClinVar v202204 and selected all the SNVss for downstream analysis. We kept variants with Pathogenic and Benign assignments ('CLNSIG'== Pathogenic or Likely_pathogenic or Benign or Likely_benign). We identified intronic variants based on Ensembl VEP annotations: only variants with at least one intronic consequence ('INTRON'== 1) in a protein-coding transcript ('BIOTYPE'= protein_coding) were retained. Additionally, we excluded variants with exonic annotations in any other gene ('EXON'≠ 1). To avoid being overly conservative, we added variants that Ensembl VEP annotated as being outside the gene body for the picked consequence ('Consequence'== TF_binding_site_variant or downstream_gene_variant or upstream_gene_variant or regulatory_region_variant), but that are annotated with intronic ontology terms in the 'MC'field in the original VCF. To minimize labeling errors, we excluded variants with less than one confidence star. To ensure that the number of benign variants did not exceed 50,000 (and therefore avoid the dataset being excessively unbalanced), we selected all higher-confidence benign variants (with two or more stars, N=13,093) along with 36,907 randomly chosen one-star variants. Finally, we retrieved the RefSeq transcript ID associated with each variant and selected only those that were intronic in such reference transcript. The dataset size for raw evaluations amounted to 18,446 pathogenic and 49,343 benign variants.

Disease-causing intronic variants affecting RNA splicing

This dataset refers to a high-quality variant set that we carefully curated to comply with the following criteria:

- Variant must locate at more than 10bp from the nearest splice site.
- Variant was experimentally proven to affect normal RNA splicing.
- Variant does not necessarily lead to pseudoexon activation.

The previous curation effort from our lab [20] was updated for this manuscript to include a comprehensive set of intronic variants identified after 2017. Therefore, the positive (disease-causing) set of variants used in this benchmark totals 242 (81 from Vaz Drago et al. (2017) and 161 from the new curation effort).

We used gnomAD v2.1 to generate a matched control set. First, we extracted all gnomAD variants occurring in a window of 500bp surrounding the variants in the positive set and selected common records with a frequency higher 0.01 (1%) in the population, resulting in 1128 variants. Then, we ran Ensembl VEP as previously described and retained the intronic variants annotated as occurring in one of the 148 unique genes of the positive set (N=1091). Moreover, we kept variants absent in ClinVar having the VCF filter field as 'PASS'(N=546). Finally, we randomly sampled 242 from this set.

Variants that affect RNA splicing

The third main dataset refers to variants that affect different mechanisms of splicing regulation, which may or may not lead to disease. We defined different molecular categories based on the location of the variant relative to the abnormal splicing event. We focused on deep intronic variants that lead to partial intron retention or pseudoexon activation. In cases where a variant leads to both pseudoexon activation and partial intron retention, we have assigned it to the pseudoexon activation group. Exceptionally, we included variants that affect the branchpoint motif (thus, closer to annotated splice acceptors) that include other types of splicing alterations such as exon skipping. We defined each category as follows:

- *Branchpoint associated*, for those variants occurring between -18 and 44bp upstream (as used in [33]) of an annotated or cryptic splicing acceptor site, and that create or disrupt any of the following adenine-branchpoint consensus motifs: YTNAY, YTNA, TNA, YNA [77].
- *Acceptor Upstream*, referring to any variant that locates between -2 and -18bp upstream of the cryptic splice acceptor (including the polypyrimidine tract).
- *New Splice Acceptor*, denoting the variants that occur at the cryptic splice acceptor positions, including the first nucleotide of the cryptic exon.
- *Exonic-like*, for any variant occurring within the cryptic exon (pseudoexon or partially retained intron).
- *New Splice Donor*, composed of variants located at the cryptic splice donor positions, including the last position of the cryptic exon.
- *Donor Downstream*, referring to any deep intronic variant that locates at a distance of more than 2bp from the activated cryptic splice donor.

We used data produced or gathered from multiple studies to assign variants to each category (Table 2). While splicing-altering variants were straightforward to assign (based on source data, the functional consequence, and distances to the splicing element considered), we distributed the non-altering variants such that they resembled as best as possible the spatial distribution of the positive sets. Hence, we assigned the negative variants taking into account two levels of information: the primary group (partial intron retention, pseudoexon activation) and the region category.

To keep in line with the expected biology, we assigned the variants that were within a defined distance to a splice site to the partial intron retention group and deeper intronic variants to the pseudoexon group. We used different distance thresholds for splice acceptors and donors (100bp and 20bp, respectively) so that the datasets were reasonably balanced. As for the region category, we defined negative variants occurring between 18 and 44bp upstream of an annotated splicing acceptor as branchpoint-associated variants. We assigned as acceptor-upstream or donor-downstream the remaining intronic variants according to whether they were located upstream or downstream of the nearest annotated splice site. Because pseudoexons tend to resemble authentic exons [85], we exceptionally assigned exonic variants that did not change inclusion levels of tested exons [86] as controls for the Exonic-like category.

Lastly, we generated control datasets for the new splice site categories. Splice site variants (located at one of the dinucleotide positions) that were experimentally tested to not affect splicing are not easily accessible. Therefore, to mimic the positive set, we looked for common deep intronic SNVs (> 5% in gnomAD v2.1) in protein-coding transcripts that generate the most common 5-mer acceptor motif CAGGT in the human genome [14] through a mutation in the core splice site dinucleotide. We randomly selected 64 variants to match the number of positive new splice acceptor variants exactly. We employed the same procedure for the new splice donor variants, where we kept the variants that generate the most common 6-mer donor motif GGTAAG in the human genome [14] at the GT position. Finally, we selected 197 variants at random to match the number of positive new splice donor variants. We confirmed using Snaptron [107] that in GTEx data, there is no evidence that a splice junction is used at the variant intervals.

Prediction tools

We selected an extensive list of prediction tools for evaluation. The single criterion for the inclusion of a tool was that it had to be designed to predict (at least partially) intronic variation. When available, we used pre-computed scores to annotate our variant sets (from dbNSFP v4.0b1 [108], UCSC genome browser [109], Zenodo or tool website). Otherwise, we ran the models directly following the developer's instructions. For a subset of splicing-related tools (MMSplice, HAL, kipoiSplice4), we employed kipoi v0.8.6 [64] to get predictions. We additionally included splicing-related tools that predict specific splicing signals (e.g., BP) and are not necessarily targeted to predict pathogenicity. Because most of these tools do not score variants by design, and some require using a web-based portal, we developed a simple utility to prepare their input given a VCF file. Moreover, we created a script for each tool to process the raw output into a final prediction score to be included in a VCF file. The package is available at [82]. We annotated the final VCF files with all the predictions using vcfanno v0.3.3 [110]. We describe all the tools, their reference thresholds and how we ran them in Table 1.

Performance evaluation

We used VETA v0.7.8 [111] to perform all the performance evaluations. We extended VETA's feature set by implementing a new mode ("–do_intronic_analysis") targeted to intronic variants. This option assigns intronic variants into distance bins based on their distance to the closest splice site. Accordingly, VETA seamlessly integrates per-bin analyses, allowing automatic inspection of how tool performance varies as one moves deeper into the intronic space. Moreover, VETA includes an *interrogate* mode that ranks candidate variants according to tool predictions, facilitating the downstream variant interpretation task in whole genome and exome studies.

In this manuscript, we employed different metrics according to the nature of the dataset and the goal of the analysis. Despite this, VETA generated confusion matrices for all tools. True Positives (TP) indicates the number of pathogenic/splicing-altering variants that a tool correctly predicts as pathogenic (or splicing-altering). True Negatives (TN) is the number of benign (or non-splicing-altering) variants that a tool scores as such. False Negatives (FN) refers to the number of true pathogenic/splicing-altering variants that a tool predicts to be benign/non-splicing-altering. Finally, False Positives (FP) stands for the number of benign/non-splicing-altering variants that a tool scores as pathogenic (or splicing-altering). For ClinVar data, we ranked variants based on the F1-score, given the unbalanced nature of the data (much more deep intronic benign variants than pathogenic). Because some tools do not score deep in the introns (missing data), we weighted the F1-score with the prediction coverage: $Coverage \cdot$

$\left(2 \cdot \frac{(Precision \cdot Recall)}{(Precision+Recall)}\right)$ where $Coverage = \frac{Scored\_variants}{Total\_variants}$, $Precision = \frac{TP}{TP+FP}$ and $Recall = \frac{TP}{TP+FN}$. For balanced datasets, we ranked tools using a slight variation of the Matthews Correlation Coefficient (MCC) ($MCC = \frac{TP \cdot TN - FP \cdot FN}{\sqrt{(TP+FP)(TP+FN)(TN+FP)(TN+FN)}}$) that normalizes the metric range between 0 and 1 ($normalizedMCC = \frac{MCC+1}{2}$). We weighted the normalized MCC values with the prediction coverage ($weighted\_normalized\_MCC = Coverage \cdot normalizedMCC$). Additionally, we employed ROC and Precision-Recall Curves (PR Curves) for the comparisons that measure performance at multiple threshold values. To summarize such analyses, we used the auROC and the auPRC metrics, respectively.

Further inspection of deep intronic variants in ClinVar

We selected ClinVar variants assigned to the "501-1000" and "+1000" intronic bins and used VEP to perform reannotation. We ran VEP using RefSeq annotations without picking any consequence ("–per_gene" and "–pick_order" were not set), meaning that all transcript consequences associated with each variant were retained. We employed a filter to only keep annotations of protein-coding transcripts. Then, we assigned each variant to one of four categories, according to the overlap configuration of transcripts belonging to the gene associated with the variant: if a variant is exonic in another overlapping transcript, we termed it as "Exonic"; if a variant is located at a shorter distance from the splice site in any other transcript, we assigned the category "> 1 transcript (smaller offset)"; if the distance to the closest splice site remains the same for all transcripts overlapping the variant, we assigned the variant to the "> 1 transcript (smaller offset)" category; lastly, if no other transcript overlapped with the variant (besides the one used in the analysis), we set it to the "No other transcript" category.

Threshold analysis for deep intronic variants

To derive clinically applicable prediction thresholds for deep intronic variants, we employed the same strategy we recently described [111]. Briefly, for each tool, we applied the F-Beta formula (at three different Beta values) over 100 threshold values uniformly distributed between the range of scores. The threshold that maximized the F-Beta function was selected. To evaluate the reliability of the adjusted thresholds, we used a bootstrapping procedure, where we kept the same ratio of pathogenic and benign variants as in the original dataset in each bootstrap. This analysis was conducted using VETA, with the options "–do_threshold_analysis" and "–bootstrapping" enabled.

Assessing quality of interpretations for SPiP, SQURLS and SpliceVault

For this task, we employed the dataset of pathogenic splicing variants used throughout the study. It includes variants from our curation plus variants from Vaz-Drago et al. [20] because the molecular mechanism for the splicing defect is known for almost all records (Supplementary Table S3). For SPiP and SQUIRLS, we ran VETA in the *interrogate* mode (with "*–labels Pathogenic*" set) to list the variants correctly predicted by each tool using the threshold calibrated for non-canonical intronic variation (SPiP > 0.009 and SQUIRLS > 0.016). We removed variants for which the ground truth information was not available (e.g.,pseudoexon-activating variants that lack details of the location of the variant concerning the cryptic event).

For SPiP, we parsed the output so that the interpretation tag, confidence interval and original score were retrieved (3rd, 4th and 5th fields after splitting predictions by "|"). We assigned variants with an "NTR" tag (low probability of affecting splicing, yet correctly predicted as pathogenic according to the calibrated threshold) to the "No interpretation" category. Variants not associated with any particular splicing mechanism (according to SPiP, the "Alter by complex event" tag) were given the "Not informative" interpretation category. Then, for each of the remaining SPiP tags we classified the interpretation as correct if they matched the ground truth information:

- "Alter BP" for variants associated with the branchpoint signal, else incorrect.
- "Alter by create new Exon" for variants that trigger pseudoexon activation, else incorrect.
- "Alter by create New splice site" for variants that create a new splice site or activate a nearby existing cryptic splice site, regardless of the variant leading to pseudoexon activation or partial intron retention, else incorrect.
- "Alter ESR" for intronic variants occurring within the boundaries of a new pseudoexon, else incorrect.
- "Alter by MES (Poly TC)" for polypyrimidine tract variants, else incorrect.

As for SQUIRLS, we ran the model for the subset of pathogenic variants correctly predicted by the tool using "–output-format html" and "–n-variants-to-report 121". Afterwards, we manually inspected the HTML report generated to derive structured interpretations for each variant: "Not informative" if the short description of the variant effect was not generated; "No interpretation" if SQUIRLS did not produce any description or figure for the variant; "New cryptic acceptor" and "New cryptic donor" if SQUIRLS described the creation of a new splice site and the variant was located at one of the splice site positions (based on the Sequence trekker figure) defined in this manuscript; "Activate cryptic acceptor" and "Activate cryptic donor" if SQUIRLS described the creation of a cryptic splice site and the variant was located outside of the splice site positions (based on the Sequence trekker figure). Because SQUIRLS does not predict the exact molecular effect of a splicing variant, we ignored the predicted number of bases affecting the coding sequence as this was not applicable for pseudoexon-activating variants. After manually inspecting the HTML report and generating structured interpretations, we classified the interpretation as correct if it matched the ground truth information:

- "New splice acceptor" for variants that create a new splice donor, else incorrect.
- "New splice donor" for variants that create a new splice acceptor, else incorrect.
- "Activate cryptic acceptor" for variants located upstream of an existing cryptic splice acceptor and not associated with the branchpoint signal, else incorrect.
- "Activate cryptic donor" for variants located downstream of an existing cryptic splice donor, else incorrect.

Finally, for SpliceVault we did not run a model to get correctly predicted variants to further inspect. Rather, SpliceVault is a web portal [112] (last accessed May 21st, 2023) to query non-canonical splicing patterns in large-scale population-based RNA-sequencing data. Because it relies on querying rare mis-splicing events with respect to annotated exons, we excluded all variants that trigger pseudoexon activation, as SpliceVault can't identify this class of events. As a result, 37 variants were left for evaluation. For each variant, we used the associated gene, intron number and molecular effect to select the correct exon and splice site to look for. We used the hg38 version (300k-RNA) and changed the default SpliceVault settings so that the Top-10 events per query were shown. Moreover, we allowed for all cryptic events to be reported, regardless of their distance to the target exon. We assigned variants to the "No interpretation" tag if the cryptic splicing event was not observed in SpliceVault Top-10 events. Then, we classified the interpretation

as “Correct” if any of the cryptic splicing triggered by the variant was observed within the Top-4 events. This threshold was recommended by the authors of SpliceVault for clinical purposes. Conversely, if the event appeared in lower ranks, we classified the interpretation as “Incorrect”.

Tissue-specific predictions by AbSplice-DNA

Throughout the manuscript, we selected the maximum AbSplice-DNA prediction for any tissue to evaluate model performance. In contrast, for this analysis, we used all predictions so that tissue specificity could be addressed. We used the same dataset as for the interpretability section. We ran VETA in the *interrogate* mode (with “’−*labels Pathogenic*” set) to list the variants correctly predicted by AbSplice-DNA using the threshold adjusted for non-canonical intronic variation (>0.004, in at least one tissue). Then, for each variant, we gathered information about the tissues associated with the disease by searching the HPO [90] with the given OMIM disease identifier. We strived to assign tissue names that matched the GTEx tissues used by AbSplice-DNA. Disease-causing variants affecting tissues not represented in GTEx (e.g. retina) were discarded. Additionally, variants causing systemic diseases (e.g. Marfan syndrome), or diseases returning ambiguous HPO terms were excluded.

Data and Source Code Availability

- Project name: DeepIntronic_Benchmark
- Project description: All data and steps to reproduce all the results of this manuscript
- Project home page: https://github.com/PedroBarbosa/DeepIntronic_Benchmark
- Operating system(s): Platform independent
- Programming language: Python
- License: GPL-3.0.
- RRID: SCR_023315

- Project name: VETA
- Project description: Software used to perform most of the analysis in the paper
- Project home page: https://github.com/PedroBarbosa/VETA
- Operating system(s): Platform independent
- Programming language: Python
- License: GPL-3.0.
- RRID: SCR_023314
- biotoolsID: veta_variantBenchmark

- Project name: PrepareSplicingPredictors
- Project description: Utilities to generate input/ process output of several sequence-based splicing predictors
- Project home page: https://github.com/PedroBarbosa/Prepare_SplicingPredictors
- Operating system(s): Platform independent
- Programming language: Python
- License: GPL-3.0.
- RRID: SCR_023316

Abbreviations

Competing Interests

The author(s) declare that they have no competing interests.

Funding

This work was supported by Fundação para a Ciência e a Tecnologia (FCT), Portugal (Fellowship to P.B. SFRH/BD/137062/2018; Exploratory Project RAP, EXPL/CCI-COM/1306/2021; and research support to LASIGE, UIDB/00408/2020 and UIDP/00408/2020), by Genomed, SA (Infogene, 045300), by FEDER/POR Lisboa 2020-Programa Operacional Regional de Lisboa, PORTUGAL 2020 (Infogene, 045300; CAMELOT, LISBOA-01-0247-FEDER-045915), and “la Caixa” Foundation under the agreement LCF/PR/HR20/52400021.

Author’s Contributions

Conceptualization: P.B., R.S., M.C-F., A.F; Funding acquisition: P.B., M.C-F, A.F.; Data curation: P.B.; Investigation: P.B.; Methodology: P.B; Resources: P.B., M.C-F; Software: P.B, A.F.; Supervision: R.S., M.C-F., A.F.; Visualization: P.B., Writing - original draft: P.B.; Writing - review & editing: P.B., R.S., M.C-F., A.F.;

Acknowledgements

Not applicable

References

1. Cooper DN. Functional Intronic Polymorphisms: Buried Treasure Awaiting Discovery within Our Genes. Human Genomics 2010 Jun;4(5):284–288.
2. Karczewski KJ, Francioli LC, Tiao G, Cummings BB, Alföldi J, Wang Q, et al. The Mutational Constraint Spectrum Quantified from Variation in 141,456 Humans. Nature 2020 May;581(7809):434–443.
3. Taliun D, Harris DN, Kessler MD, Carlson J, Szpiech ZA, Torres R, et al. Sequencing of 53,831 Diverse Genomes from the NHLBI TOPMed Program. Nature 2021 Feb;590(7845):290–299.
4. Eilbeck K, Quinlan A, Yandell M. Settling the Score: Variant Prioritization and Mendelian Disease. Nature Reviews Genetics 2017 Oct;18(10):599–612.
5. Lord J, Baralle D. Splicing in the Diagnosis of Rare Disease: Advances and Challenges. Frontiers in Genetics 2021;12.
6. Wahl MC, Will CL, Lührmann R. The Spliceosome: Design Principles of a Dynamic RNP Machine. Cell 2009 Feb;136(4):701–718.
7. Ward AJ, Cooper TA. The Pathobiology of Splicing. The Journal of pathology 2010 Jan;220(2):152–163.
8. Wang GS, Cooper TA. Splicing in Disease: Disruption of the Splicing Code and the Decoding Machinery. Nature Reviews Genetics 2007 Oct;8(10):749–761.
9. Lim KH, Ferraris L, Filloux ME, Raphael BJ, Fairbrother WG. Using Positional Distribution to Identify Splicing Elements and Predict Pre-mRNA Processing Defects in Human Genes. Proceedings of the National Academy of Sciences 2011 Jul;108(27):11093–11098.
10. Jaganathan K, Kyriazopoulou Panagiotopoulou S, McRae JF, Darbandi SF, Knowles D, Li YI, et al. Predicting Splicing from Primary Sequence with Deep Learning. Cell 2019 Jan;176(3):535–548.e24.
11. Desterro J, Bak-Gordon P, Carmo-Fonseca M. Targeting mRNA Processing as an Anticancer Strategy. Nature Reviews Drug Discovery 2020 Feb;19(2):112–129.
12. Anna A, Monika G. Splicing Mutations in Human Genetic Disorders: Examples, Detection, and Confirmation. Journal of Applied Genetics 2018;59(3):253–268.
13. Ule J, Blencowe BJ. Alternative Splicing Regulatory Networks:

Functions, Mechanisms, and Evolution. Molecular Cell 2019 Oct;76(2):329–345.
14. Sibley CR, Blazquez L, Ule J. Lessons from Non-Canonical Splicing. Nature Reviews Genetics 2016 Jul;17(7):407–421.
15. Landrum MJ, Lee JM, Benson M, Brown GR, Chao C, Chitipiralla S, et al. ClinVar: Improving Access to Variant Interpretations and Supporting Evidence. Nucleic Acids Research 2018 Jan;46(D1):D1062–D1067.
16. Stenson PD, Mort M, Ball EV, Chapman M, Evans K, Azevedo L, et al. The Human Gene Mutation Database (HGMD®): Optimizing Its Use in a Clinical Diagnostic or Research Setting. Human Genetics 2020;139(10):1197–1207.
17. Lord J, Gallone G, Short PJ, McRae JF, Ironfield H, Wynn EH, et al. Pathogenicity and Selective Constraint on Variation near Splice Sites. Genome Research 2019 Feb;29(2):159–170.
18. Blakes AJM, Wai HA, Davies I, Moledina HE, Ruiz A, Thomas T, et al. A Systematic Analysis of Splicing Variants Identifies New Diagnoses in the 100,000 Genomes Project. Genome Medicine 2022 Jul;14(1):79.
19. Ellingford JM, Ahn JW, Bagnall RD, Baralle D, Barton S, Campbell C, et al. Recommendations for Clinical Interpretation of Variants Found in Non-Coding Regions of the Genome. Genome Medicine 2022 Jul;14(1):73.
20. Vaz-Drago R, Custódio N, Carmo-Fonseca M. Deep Intronic Mutations and Human Disease. Human Genetics 2017 Sep;136(9):1093–1111.
21. Keegan NP, Wilton SD, Fletcher S. Analysis of Pathogenic Pseudoexons Reveals Novel Mechanisms Driving Cryptic Splicing. Frontiers in Genetics 2022;12.
22. Lek M, Karczewski KJ, Minikel EV, Samocha KE, Banks E, Fennell T, et al. Analysis of Protein-Coding Genetic Variation in 60,706 Humans. Nature 2016 Aug;536(7616):285–291.
23. Dunham I, Kundaje A, Aldred SF, Collins PJ, Davis CA, Doyle F, et al. An Integrated Encyclopedia of DNA Elements in the Human Genome. Nature 2012 Sep;489(7414):57–74.
24. Eraslan G, Avsec Ž, Gagneur J, Theis FJ. Deep Learning: New Computational Modelling Techniques for Genomics. Nature Reviews Genetics 2019 Jul;20(7):389–403.
25. Cormier MJ, Pedersen BS, Bayrak-Toydemir P, Quinlan AR. Combining Genetic Constraint with Predictions of Alternative Splicing to Prioritize Deleterious Splicing in Rare Disease Studies. BMC Bioinformatics 2022 Nov;23(1):482.
26. Kurosawa R, Iida K, Ajiro M, Awaya T, Yamada M, Kosaki K, et al., PDIVAS: Pathogenicity Predictor for Deep-Intronic Variants Causing Aberrant Splicing. medRxiv; 2023.
27. Wagner N, Çelik MH, Hölzlwimmer FR, Mertes C, Prokisch H, Yépez VA, et al. Aberrant Splicing Prediction across Human Tissues. Nature Genetics 2023 May;55(5):861–870.
28. Zeng T, Li YI. Predicting RNA Splicing from DNA Sequence Using Pangolin. Genome Biology 2022 Apr;23(1):103.
29. Strauch Y, Lord J, Niranjan M, Baralle D. CI-SpliceAI—Improving Machine Learning Predictions of Disease Causing Splicing Variants Using Curated Alternative Splice Sites. PLOS ONE 2022 Jun;17(6):e0269159.
30. Frankish A, Diekhans M, Ferreira AM, Johnson R, Jungreis I, Loveland J, et al. GENCODE Reference Annotation for the Human and Mouse Genomes. Nucleic Acids Research 2019 Jan;47(D1):D766–D773.
31. Weber LM, Saelens W, Cannoodt R, Soneson C, Hapfelmeier A, Gardner PP, et al. Essential Guidelines for Computational Method Benchmarking. Genome Biology 2019 Jun;20(1):125.
32. Buchka S, Hapfelmeier A, Gardner PP, Wilson R, Boulesteix AL. On the Optimistic Performance Evaluation of Newly Introduced Bioinformatic Methods. Genome Biology 2021 May;22(1):152.
33. Leman R, Tubeuf H, Raad S, Tournier I, Derambure C, Lanos R, et al. Assessment of Branch Point Prediction Tools to Predict Physiological Branch Points and Their Alteration by Variants. BMC Genomics 2020 Jan;21(1):86.
34. Tubeuf H, Charbonnier C, Soukarieh O, Blavier A, Lefebvre A, Dauchel H, et al. Large-Scale Comparative Evaluation of User-Friendly Tools for Predicting Variant-Induced Alterations of Splicing Regulatory Elements. Human Mutation 2020;41(10):1811–1829.
35. Moles-Fernández A, Domènech-Vivó J, Tenés A, Balmaña J, Diez O, Gutiérrez-Enríquez S. Role of Splicing Regulatory Elements and In Silico Tools Usage in the Identification of Deep Intronic Splicing Variants in Hereditary Breast/Ovarian Cancer Genes. Cancers 2021 Jul;13(13):3341.
36. Riepe TV, Khan M, Roosing S, Cremers FPM, ’t Hoen PAC. Benchmarking Deep Learning Splice Prediction Tools Using Functional Splice Assays. Human Mutation 2021;42(7):799–810.
37. Rowlands C, Thomas HB, Lord J, Wai HA, Arno G, Beaman G, et al. Comparison of in Silico Strategies to Prioritize Rare Genomic Variants Impacting RNA Splicing for the Diagnosis of Genomic Disorders. Scientific Reports 2021 Oct;11(1):20607.
38. Ha C, Kim JW, Jang JH. Performance Evaluation of SpliceAI for the Prediction of Splicing of NF1 Variants. Genes 2021 Sep;12(9):1308.
39. Li K, Luo T, Zhu Y, Huang Y, Wang A, Zhang D, et al. Performance Evaluation of Differential Splicing Analysis Methods and Splicing Analytics Platform Construction. Nucleic Acids Research 2022 Aug;p. gkac686.
40. Leman R, Parfait B, Vidaud D, Girodon E, Pacot L, Le Gac G, et al. SPiP: Splicing Prediction Pipeline, a Machine Learning Tool for Massive Detection of Exonic and Intronic Variant Effect on mRNA Splicing. Human Mutation;n/a(n/a).
41. Li S, van der Velde KJ, de Ridder D, van Dijk ADJ, Soudis D, Zwerwer LR, et al. CAPICE: A Computational Method for Consequence-Agnostic Pathogenicity Interpretation of Clinical Exome Variations. Genome Medicine 2020 Aug;12(1):75.
42. Siepel A, Bejerano G, Pedersen JS, Hinrichs AS, Hou M, Rosenbloom K, et al. Evolutionarily Conserved Elements in Vertebrate, Insect, Worm, and Yeast Genomes. Genome Research 2005 Aug;15(8):1034–1050.
43. Li J, Zhao T, Zhang Y, Zhang K, Shi L, Chen Y, et al. Performance Evaluation of Pathogenicity-Computation Methods for Missense Variants. Nucleic Acids Research 2018 Sep;46(15):7793–7804.
44. Siepel A, Pollard KS, Haussler D. New Methods for Detecting Lineage-Specific Selection. In: Apostolico A, Guerra C, Istrail S, Pevzner PA, Waterman M, editors. Research in Computational Molecular Biology Lecture Notes in Computer Science, Berlin, Heidelberg: Springer; 2006. p. 190–205.
45. Dong C, Wei P, Jian X, Gibbs R, Boerwinkle E, Wang K, et al. Comparison and Integration of Deleteriousness Prediction Methods for Nonsynonymous SNVs in Whole Exome Sequencing Studies. Human Molecular Genetics 2015 Apr;24(8):2125–2137.
46. Garber M, Guttman M, Clamp M, Zody MC, Friedman N, Xie X. Identifying Novel Constrained Elements by Exploiting Biased Substitution Patterns. Bioinformatics 2009 Jun;25(12):i54–i62.
47. Davydov EV, Goode DL, Sirota M, Cooper GM, Sidow A, Batzoglou S. Identifying a High Fraction of the Human Genome to Be under Selective Constraint Using GERP++. PLoS Computational Biology 2010 Dec;6(12):e1001025.
48. Shihab HA, Rogers MF, Gough J, Mort M, Cooper DN, Day INM, et al. An Integrative Approach to Predicting the Functional Effects of Non-Coding and Coding Sequence Variation. Bioinformatics (Oxford, England) 2015 May;31(10):1536–1543.
49. Liu X, Wu C, Li C, Boerwinkle E. dbNSFP v3.0: A One-Stop Database of Functional Predictions and Annotations for Human Non-synonymous and Splice Site SNVs. Human mutation 2016 Mar;37(3):235–241.

50. 1000 Genomes Project Consortium, Auton A, Brooks LD, Durbin RM, Garrison EP, Kang HM, et al. A Global Reference for Human Genetic Variation. Nature 2015 Oct;526(7571):68–74.
51. Ionita-Laza I, McCallum K, Xu B, Buxbaum JD. A Spectral Approach Integrating Functional Genomic Annotations for Coding and Noncoding Variants. Nature Genetics 2016 Feb;48(2):214–220.
52. Jagadeesh KA, Paggi JM, Ye JS, Stenson PD, Cooper DN, Bernstein JA, et al. S-CAP Extends Pathogenicity Prediction to Genetic Variants That Affect RNA Splicing. Nature genetics 2019 Feb;51(4):755–763.
53. Smedley D, Schubach M, Jacobsen JOB, Köhler S, Zemojtel T, Spielmann M, et al. A Whole-Genome Analysis Framework for Effective Identification of Pathogenic Regulatory Variants in Mendelian Disease. The American Journal of Human Genetics 2016 Sep;99(3):595–606.
54. Huang YF, Gulko B, Siepel A. Fast, Scalable Prediction of Deleterious Noncoding Variants from Functional and Population Genomic Data. Nature Genetics 2017 Apr;49(4):618–624.
55. Fokkema IFAC, van der Velde KJ, Slofstra MK, Ruivenkamp CAL, Vogel MJ, Pfundt R, et al. Dutch Genome Diagnostic Laboratories Accelerated and Improved Variant Interpretation and Increased Accuracy by Sharing Data. Human Mutation 2019;40(12):2230–2238.
56. Rentzsch P, Schubach M, Shendure J, Kircher M. CADD-Splice—Improving Genome-Wide Variant Effect Prediction Using Deep Learning-Derived Splice Scores. Genome Medicine 2021 Feb;13(1):31.
57. Yeo G, Burge CB. Maximum Entropy Modeling of Short Sequence Motifs with Applications to RNA Splicing Signals. Journal of Computational Biology 2004 Mar;11(2-3):377–394.
58. Shamsani J, Kazakoff SH, Armean IM, McLaren W, Parsons MT, Thompson BA, et al. A Plugin for the Ensembl Variant Effect Predictor That Uses MaxEntScan to Predict Variant Spliceogenicity. Bioinformatics 2019 Jul;35(13):2315–2317.
59. Jian X, Boerwinkle E, Liu X. In Silico Prediction of Splice-Altering Single Nucleotide Variants in the Human Genome. Nucleic Acids Research 2014 Dec;42(22):13534–13544.
60. Wang J, Zhang J, Li K, Zhao W, Cui Q. SpliceDisease Database: Linking RNA Splicing and Disease. Nucleic Acids Research 2012 Jan;40(Database issue):D1055–1059.
61. Xiong HY, Alipanahi B, Lee LJ, Bretschneider H, Merico D, Yuen RKC, et al. The Human Splicing Code Reveals New Insights into the Genetic Determinants of Disease. Science (New York, NY) 2015;347(6218).
62. Rosenberg AB, Patwardhan RP, Shendure J, Seelig G. Learning the Sequence Determinants of Alternative Splicing from Millions of Random Sequences. Cell 2015 Oct;163(3):698–711.
63. Gelfman S, Wang Q, McSweeney KM, Ren Z, La Carpia F, Halvorsen M, et al. Annotating Pathogenic Non-Coding Variants in Genic Regions. Nature Communications 2017 Aug;8(1):236.
64. Avsec Ž, Kreuzhuber R, Israeli J, Xu N, Cheng J, Shrikumar A, et al. The Kipoi Repository Accelerates Community Exchange and Reuse of Predictive Models for Genomics. Nature Biotechnology 2019;37(6):592–600.
65. Lonsdale J, Thomas J, Salvatore M, Phillips R, Lo E, Shad S, et al. The Genotype-Tissue Expression (GTEx) Project. Nature Genetics 2013 Jun;45(6):580–585.
66. Cheng J, Nguyen TYD, Cygan KJ, Çelik MH, Fairbrother WG, Avsec ž, et al. MMSplice: Modular Modeling Improves the Predictions of Genetic Variant Effects on Splicing. Genome Biology 2019 Mar;20(1):48.
67. Danis D, Jacobsen JOB, Carmody LC, Gargano MA, McMurry JA, Hegde A, et al. Interpretable Prioritization of Splice Variants in Diagnostic Next-Generation Sequencing. American Journal of Human Genetics 2021 Sep;108(9):1564–1577.
68. Liu H, Dai J, Li K, Sun Y, Wei H, Wang H, et al. Performance Evaluation of Computational Methods for Splice-Disrupting Variants and Improving the Performance Using the Machine Learning-Based Framework. Briefings in Bioinformatics 2022 Aug;p. bbac334.
69. Sherry ST, Ward MH, Kholodov M, Baker J, Phan L, Smigielski EM, et al. dbSNP: The NCBI Database of Genetic Variation. Nucleic Acids Research 2001 Jan;29(1):308–311.
70. Cartegni L, Wang J, Zhu Z, Zhang MQ, Krainer AR. ESEfinder: A Web Resource to Identify Exonic Splicing Enhancers. Nucleic Acids Research 2003 Jul;31(13):3568–3571.
71. Ke S, Shang S, Kalachikov SM, Morozova I, Yu L, Russo JJ, et al. Quantitative Evaluation of All Hexamers as Exonic Splicing Elements. Genome Research 2011 Aug;21(8):1360–1374.
72. Erkelenz S, Theiss S, Otte M, Widera M, Peter JO, Schaal H. Genomic HEXploring Allows Landscaping of Novel Potential Splicing Regulatory Elements. Nucleic Acids Research 2014;42(16):10681–10697.
73. Takeda Ji, Fukami S, Tamura A, Shibata A, Ohno K. IntSplice2: Prediction of the Splicing Effects of Intronic Single-Nucleotide Variants Using LightGBM Modeling. Frontiers in Genetics 2021;12.
74. Corvelo A, Hallegger M, Smith CWJ, Eyras E. Genome-Wide Association between Branch Point Properties and Alternative Splicing. PLOS Computational Biology 2010 Nov;6(11):e1001016.
75. Zhang Q, Fan X, Wang Y, Sun Ma, Shao J, Guo D. BPP: A Sequence-Based Algorithm for Branch Point Prediction. Bioinformatics 2017 Oct;33(20):3166–3172.
76. Paggi JM, Bejerano G. A Sequence-Based, Deep Learning Model Accurately Predicts RNA Splicing Branchpoints. RNA 2018 Dec;24(12):1647–1658.
77. Zhang P, Philippot Q, Ren W, Lei WT, Li J, Stenson PD, et al. Genome-Wide Detection of Human Variants That Disrupt Intronic Branchpoints. Proceedings of the National Academy of Sciences 2022 Nov;119(44):e2211194119.
78. Zuallaert J, Godin F, Kim M, Soete A, Saeys Y, De Neve W. SpliceRover: Interpretable Convolutional Neural Networks for Improved Splice Site Prediction. Bioinformatics (Oxford, England) 2018 Dec;34(24):4180–4188.
79. Naito T. Predicting the Impact of Single Nucleotide Variants on Splicing via Sequence-based Deep Neural Networks and Genomic Features. Human Mutation 2019 May;p. humu.23794.
80. Soemedi R, Cygan KJ, Rhine CL, Wang J, Bulacan C, Yang J, et al. Pathogenic Variants That Alter Protein Code Often Disrupt Splicing. Nature genetics 2017 Jun;49(6):848–855.
81. Scalzitti N, Kress A, Orhand R, Weber T, Moulinier L, Jeannin-Girardon A, et al. Spliceator: Multi-Species Splice Site Prediction Using Convolutional Neural Networks. BMC Bioinformatics 2021 Nov;22(1):561.
82. Preparing Input for Multiple Splicing Predictors;. https://github.com/PedroBarbosa/Prepare_SplicingPredictors.
83. Grimm DG, Azencott CA, Aicheler F, Gieraths U, MacArthur DG, Samocha KE, et al. The Evaluation of Tools Used to Predict the Impact of Missense Variants Is Hindered by Two Types of Circularity. Human mutation 2015 May;36(5):513–23.
84. Jung H, Lee KS, Choi JK. Comprehensive Characterisation of Intronic Mis-Splicing Mutations in Human Cancers. Oncogene 2021 Feb;40(7):1347–1361.
85. Petersen USS, Doktor TK, Andresen BS. Pseudoexon Activation in Disease by Non-Splice Site Deep Intronic Sequence Variation — Wild Type Pseudoexons Constitute High-Risk Sites in the Human Genome. Human Mutation 2022;43(2):103–127.
86. Adamson SI, Zhan L, Graveley BR. Vex-Seq: High-Throughput Identification of the Impact of Genetic Variation on Pre-mRNA Splicing Efficiency. Genome Biology 2018 Jun;19(1):71.
87. Cheung R, Insigne KD, Yao D, Burghard CP, Wang J, Hsiao YHE, et al. A Multiplexed Assay for Exon Recognition Reveals That an Unappreciated Fraction of Rare Genetic Variants Cause Large-

Effect Splicing Disruptions. Molecular cell 2019 Jan;73(1):183–194.e8.
88. SpliceAI Lookup API;. https://spliceailookup.broadinstitute.org/.
89. Dawes R, Bournazos AM, Bryen SJ, Bommireddipalli S, Marchant RG, Joshi H, et al. SpliceVault Predicts the Precise Nature of Variant-Associated Mis-Splicing. Nature Genetics 2023 Feb;55(2):324–332.
90. Köhler S, Gargano M, Matentzoglu N, Carmody LC, Lewis-Smith D, Vasilevsky NA, et al. The Human Phenotype Ontology in 2021. Nucleic Acids Research 2021 Jan;49(D1):D1207–D1217.
91. Richards S, Aziz N, Bale S, Bick D, Das S, Gastier-Foster J, et al. Standards and Guidelines for the Interpretation of Sequence Variants: A Joint Consensus Recommendation of the American College of Medical Genetics and Genomics and the Association for Molecular Pathology. Genetics in medicine : official journal of the American College of Medical Genetics 2015 May;17(5):405–424.
92. Schoch K, Tan QKG, Stong N, Deak KL, McConkie-Rosell A, McDonald MT, et al. Alternative Transcripts in Variant Interpretation: The Potential for Missed Diagnoses and Misdiagnoses. Genetics in Medicine 2020 Jul;22(7):1269–1275.
93. Canson D, Glubb D, Spurdle AB. Variant Effect on Splicing Regulatory Elements, Branchpoint Usage, and Pseudoexonization: Strategies to Enhance Bioinformatic Prediction Using Hereditary Cancer Genes as Exemplars. Human Mutation 2020;41(10):1705–1721.
94. Grodecká L, Buratti E, Freiberger T. Mutations of Pre-mRNA Splicing Regulatory Elements: Are Predictions Moving Forward to Clinical Diagnostics? International Journal of Molecular Sciences 2017 Jul;18(8):1668.
95. Gebauer F, Schwarzl T, Valcárcel J, Hentze MW. RNA-binding Proteins in Human Genetic Disease. Nature Reviews Genetics 2021 Mar;22(3):185–198.
96. Ching T, Himmelstein DS, Beaulieu-Jones BK, Kalinin AA, Do BT, Way GP, et al. Opportunities and Obstacles for Deep Learning in Biology and Medicine. Journal of The Royal Society Interface 2018 Apr;15(141):20170387.
97. Novakovsky G, Dexter N, Libbrecht MW, Wasserman WW, Mostafavi S. Obtaining Genetics Insights from Deep Learning via Explainable Artificial Intelligence. Nature Reviews Genetics 2022 Oct;p. 1–13.
98. Aicher JK, Jewell P, Vaquero-Garcia J, Barash Y, Bhoj EJ. Mapping RNA Splicing Variations in Clinically-Accessible and Non-Accessible Tissues to Facilitate Mendelian Disease Diagnosis Using RNA-seq. Genetics in medicine : official journal of the American College of Medical Genetics 2020 Jul;22(7):1181.
99. Smith C, Kitzman JO. Benchmarking Splice Variant Prediction Algorithms Using Massively Parallel Splicing Assays. bioRxiv 2023 May;p. 2023.05.04.539398.
100. de Sainte Agathe JM, Filser M, Isidor B, Besnard T, Gueguen P, Perrin A, et al. SpliceAI-visual: A Free Online Tool to Improve SpliceAI Splicing Variant Interpretation. Human Genomics 2023 Feb;17:7.
101. MobiDetails;. https://mobidetails.iurc.montp.inserm.fr/MD.
102. CI-SpliceAI Online Service;. https://ci-spliceai.com/.
103. Wolf T, Debut L, Sanh V, Chaumond J, Delangue C, Moi A, et al., HuggingFace's Transformers: State-of-the-art Natural Language Processing. arXiv; 2020.
104. Avsec Ž, Agarwal V, Visentin D, Ledsam JR, Grabska-Barwinska A, Taylor KR, et al. Effective Gene Expression Prediction from Sequence by Integrating Long-Range Interactions. Nature Methods 2021 Oct;18(10):1196–1203.
105. Meier J, Rao R, Verkuil R, Liu J, Sercu T, Rives A, Language Models Enable Zero-Shot Prediction of the Effects of Mutations on Protein Function. bioRxiv; 2021.
106. McLaren W, Gil L, Hunt SE, Riat HS, Ritchie GRS, Thormann A, et al. The Ensembl Variant Effect Predictor. Genome Biology 2016 Jun;17(1):122.
107. Wilks C, Gaddipati P, Nellore A, Langmead B. Snaptron: Querying Splicing Patterns across Tens of Thousands of RNA-seq Samples. Bioinformatics 2018 Jan;34(1):114–116.
108. Liu X, Li C, Mou C, Dong Y, Tu Y. dbNSFP v4: A Comprehensive Database of Transcript-Specific Functional Predictions and Annotations for Human Nonsynonymous and Splice-Site SNVs. Genome Medicine 2020 Dec;12(1):103.
109. Kent WJ, Sugnet CW, Furey TS, Roskin KM, Pringle TH, Zahler AM, et al. The Human Genome Browser at UCSC. Genome Research 2002 Jun;12(6):996–1006.
110. Pedersen BS, Layer RM, Quinlan AR. Vcfanno: Fast, Flexible Annotation of Genetic Variants. Genome Biology 2016 Jun;17(1):118.
111. Barbosa P, Ribeiro M, Carmo-Fonseca M, Fonseca A. Clinical Significance of Genetic Variation in Hypertrophic Cardiomyopathy: Comparison of Computational Tools to Prioritize Missense Variants. Frontiers in Cardiovascular Medicine 2022;9.
112. SpliceVault Portal;. https://kidsneuro.shinyapps.io/splicevault/.

1615 ## Supplementary figures

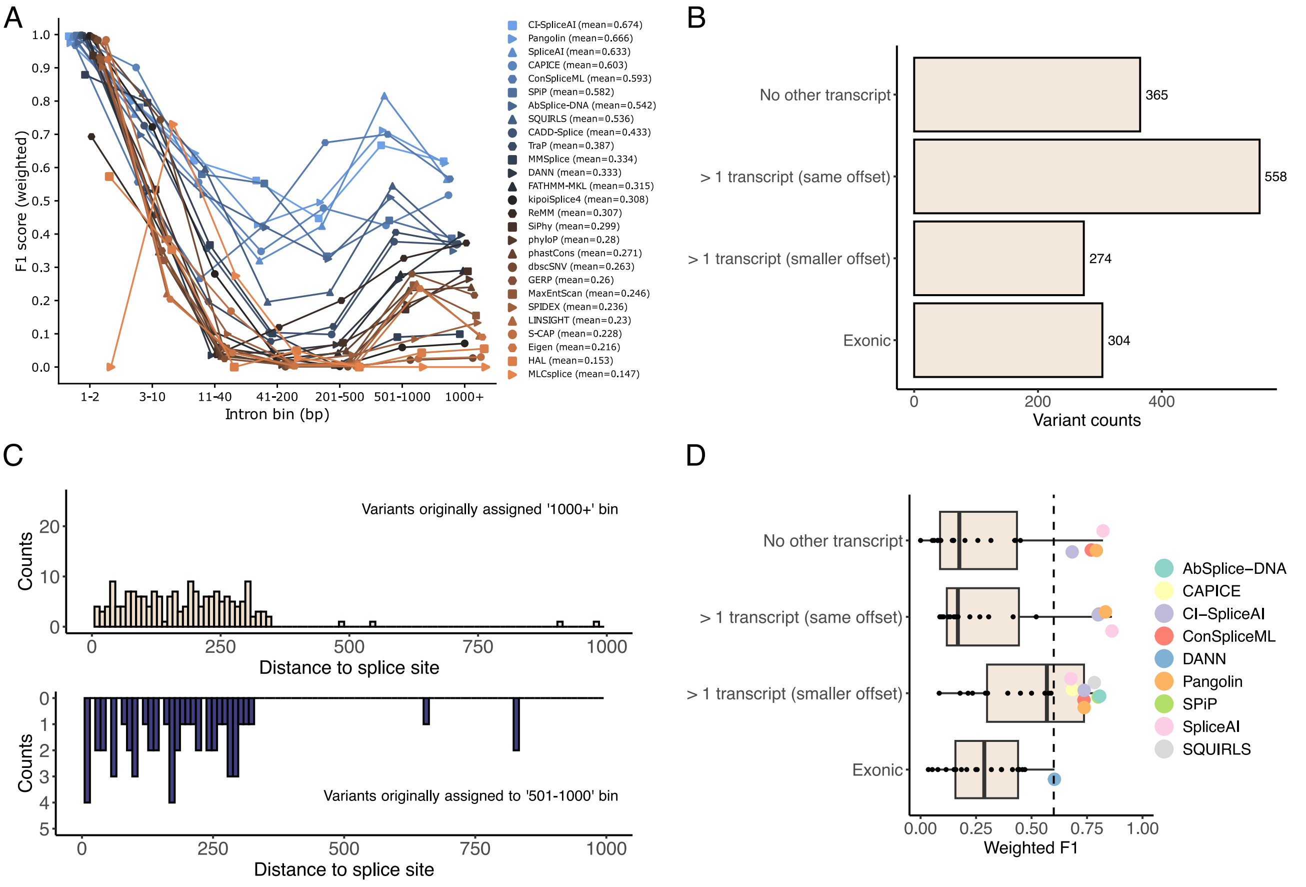

Supplementary Figure S 1. Intronic variant prediction in ClinVar. **A** Performance of all tools considered for the study on the raw ClinVar intronic dataset. Mean values in the legend represent the average weighted F1 score across all intronic bins. **B** - Inspection of intronic variants (after addressing circularity problems) assigned to the "501-1000" and "1000+" intronic bins. The bars reflect the number of variants assigned to each category. The term "No other transcript" refers to all variants that do not have any other RefSeq protein coding transcript of the same gene overlapping with them, besides the transcript originally used (N pathogenic=25, N benign=340). "> 1 transcript (same offset)" refers to variants that overlap with more than one transcript of the same gene but do not have any other transcript where the variant is closer to the splice site than in the original transcript used in the analysis (N pathogenic=31, N benign=527). "> 1 transcript (smaller offset)" refers to variants that overlap with more than one transcript of the same gene, and have at least one other transcript in which the variant is closer to the splice site than in the original transcript used in the analysis (N pathogenic=20, N benign=254). "Exonic" refers to variants that overlap with more than one transcript of the same gene, and have at least one other transcript where the variant is exonic (N pathogenic=41, N benign=263). **C** - Distribution of the updated intronic distances to the closest splice site for variants assigned to the "> 1 transcript (smaller offset)" category. **D** - Tool performance (measured with weighted F1 score) for each individual category. Tools with performance higher than 0.6 are highlighted.

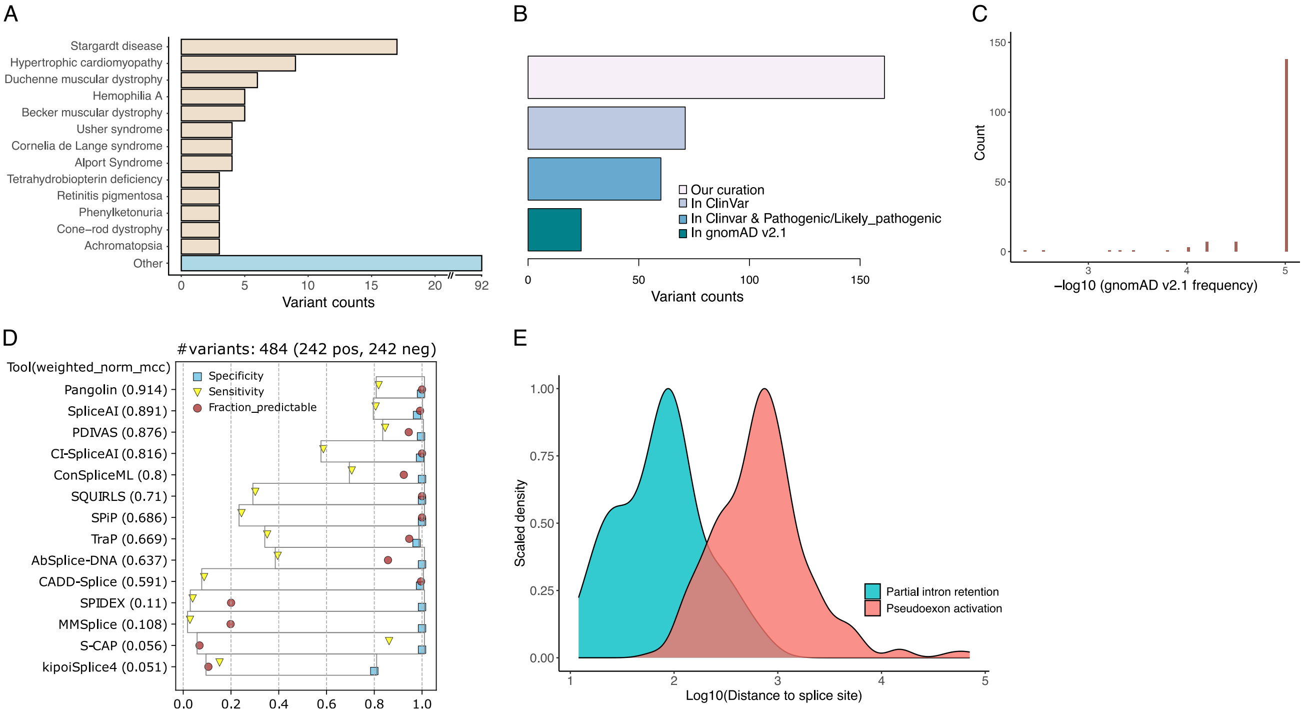

Supplementary Figure S 2. Manually curated dataset of pathogenic intronic variants disrupting RNA splicing. **A** - Number of variants collected per phenotype. Diseases with less than 3 variants were assigned to the 'Other' category. **B** - Number of variants occurring in ClinVar and gnomAD v2.1. **C** - Log transformed allele frequencies of variants in gnomAD v2.1. For those that are absent in the database, a pseudocount of 0.00001 was added (highest histogram peak, at 5). **D** - Tool performance using reference thresholds from Table 1 for variants curated in this manuscript plus those curated by Vaz-Drago et al. [20]. MLCsplice and dbscSNV are not shown as they had more than 95% of missing predictions. **E** - Distance (Log10) of the variants to the closest splice junction. Pseudoexon activation group: 194 variants; Partial intron retention group: 37 variants.

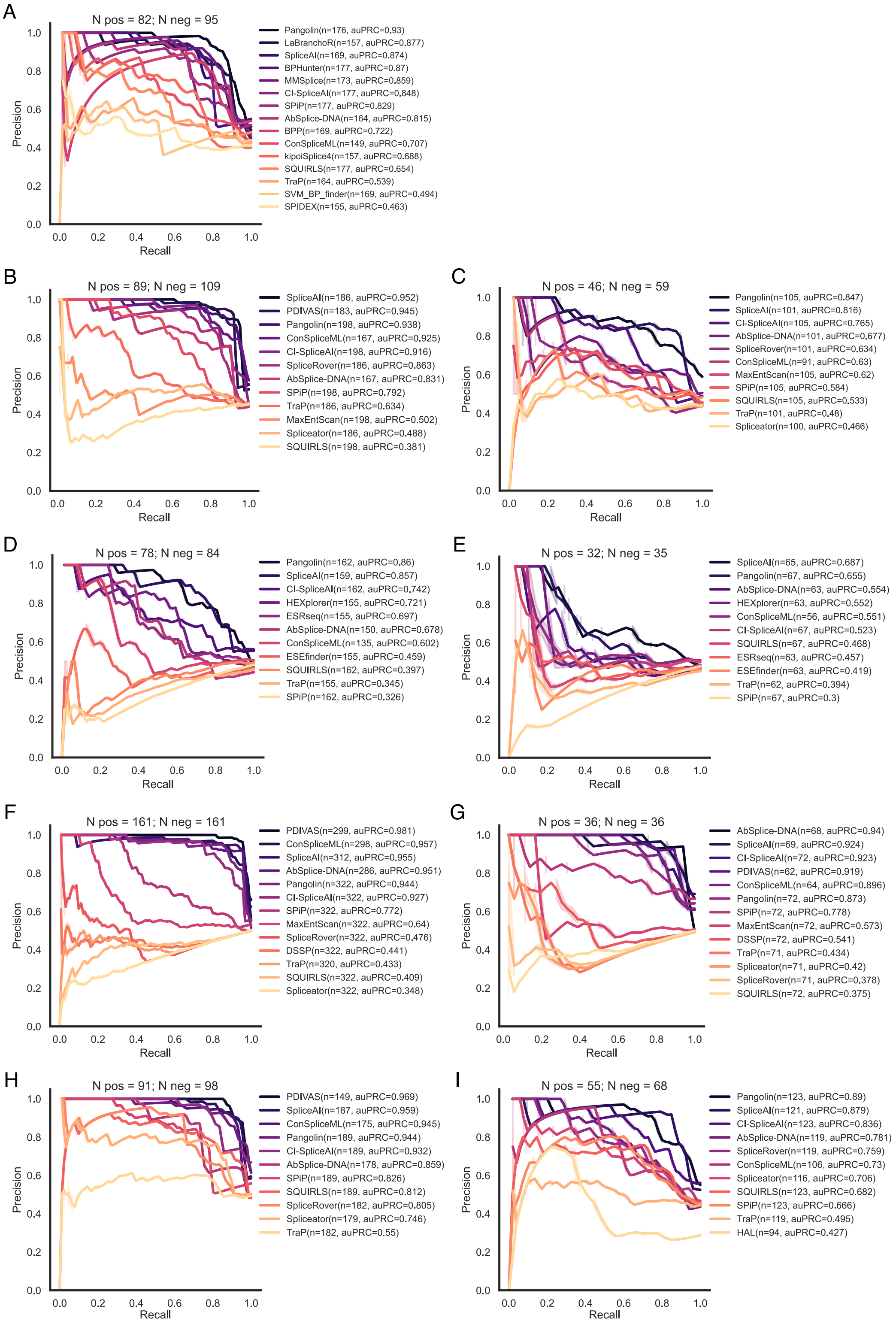

Supplementary Figure S 3. Precision-Recall curves for all splicing-altering variants analyzed in a region-specific manner. Tools are ranked by the auPRC metric and the number of predictions made by each tool is displayed in "n=". The number of variants in each dataset is presented ("N pos" represents the number of positive splicing altering variants; "N neg" is the number of negative splicing variants). Tools with more than 50% of missing predictions or with less than 15 variants in the minority class were excluded from these analyses. For the Exonic-like category, PDIVAS was excluded because control variants in this comparison are exonic, which is outside of the scope of this model. **A** - Branchpoint associated variants. **B** - Acceptor-associated variants that trigger pseudoexon inclusion. **C** - Acceptor-associated variants that lead to partial intron retention. **D** - Exonic-like variants that trigger pseudoexon inclusion. **E** - Exonic-like variants that lead to partial intron retention. **F** - Variants that create new splice donors and activate pseudoexons. **G** - Variants that create new splice donors and lead to partial intron retention. **H** - Variants that activate existing upstream cryptic splice donors and trigger pseudoexon activation. **I** - Variants that activate existing upstream cryptic splice donors and lead to partial intron retention.

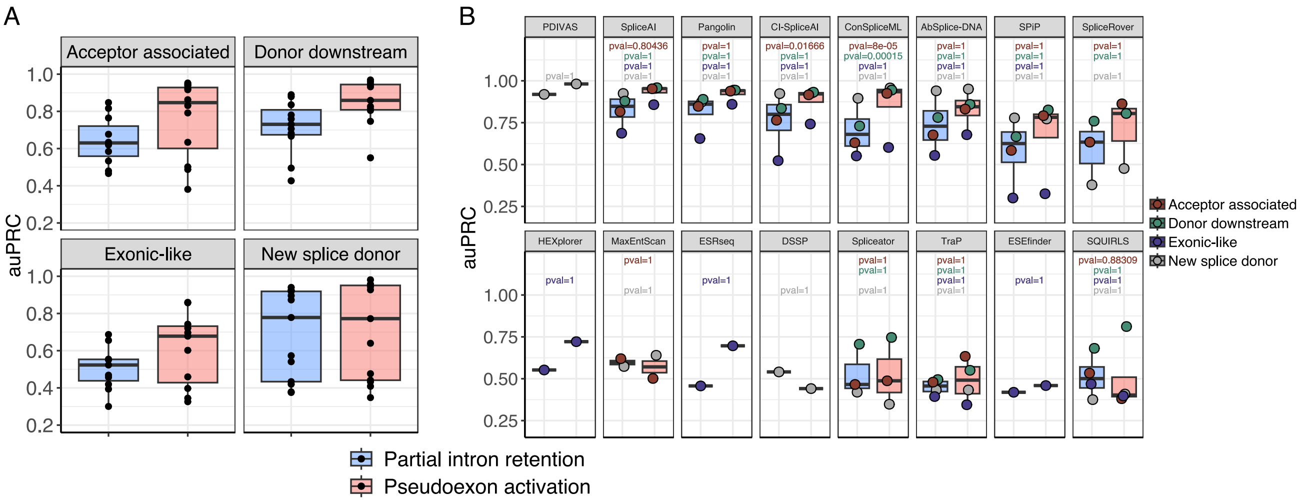

Supplementary Figure S 4. Comparing performance between all pseudoexon activation versus partial intron retention variants collected in this study. **A** - Distribution of the auPRC scores of the tools for each variant region. **B** - Per-tool auPRC distribution across the four variant regions considered. A Fisher's exact test was conducted separately for each tool and variant region to determine statistical significance for the performance differences between the pseudoexon activation and partial intron retention groups. The true positives plus true negatives were considered successful outcomes, while false positives plus false negatives were considered failures. The p-values displayed in the figure were corrected for multiple comparisons using the Holm method. For each tool, we excluded the variant regions that did not have performance measurements in both groups.

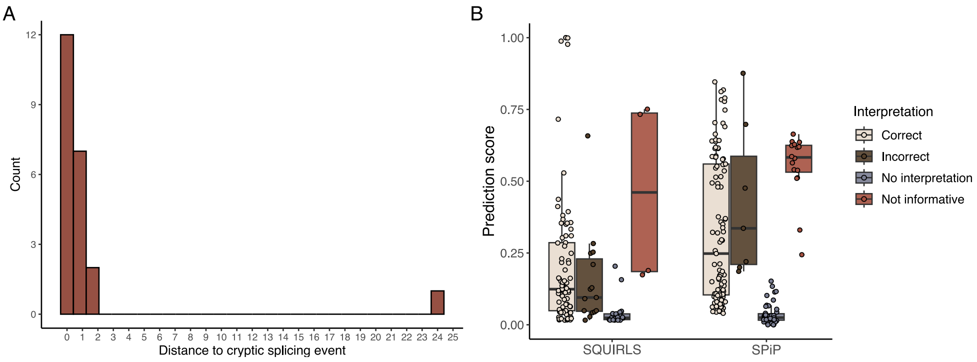

Supplementary Figure S 5. Interpretability assessment. A) Distance of the variants to the activated cryptic splice site (relative to the first splice site position) in SpliceVault analysis for the variants assigned to the "No interpretation" tag. B) Distribution of SPiP and SQUIRLS prediction values for each of the interpretation categories.

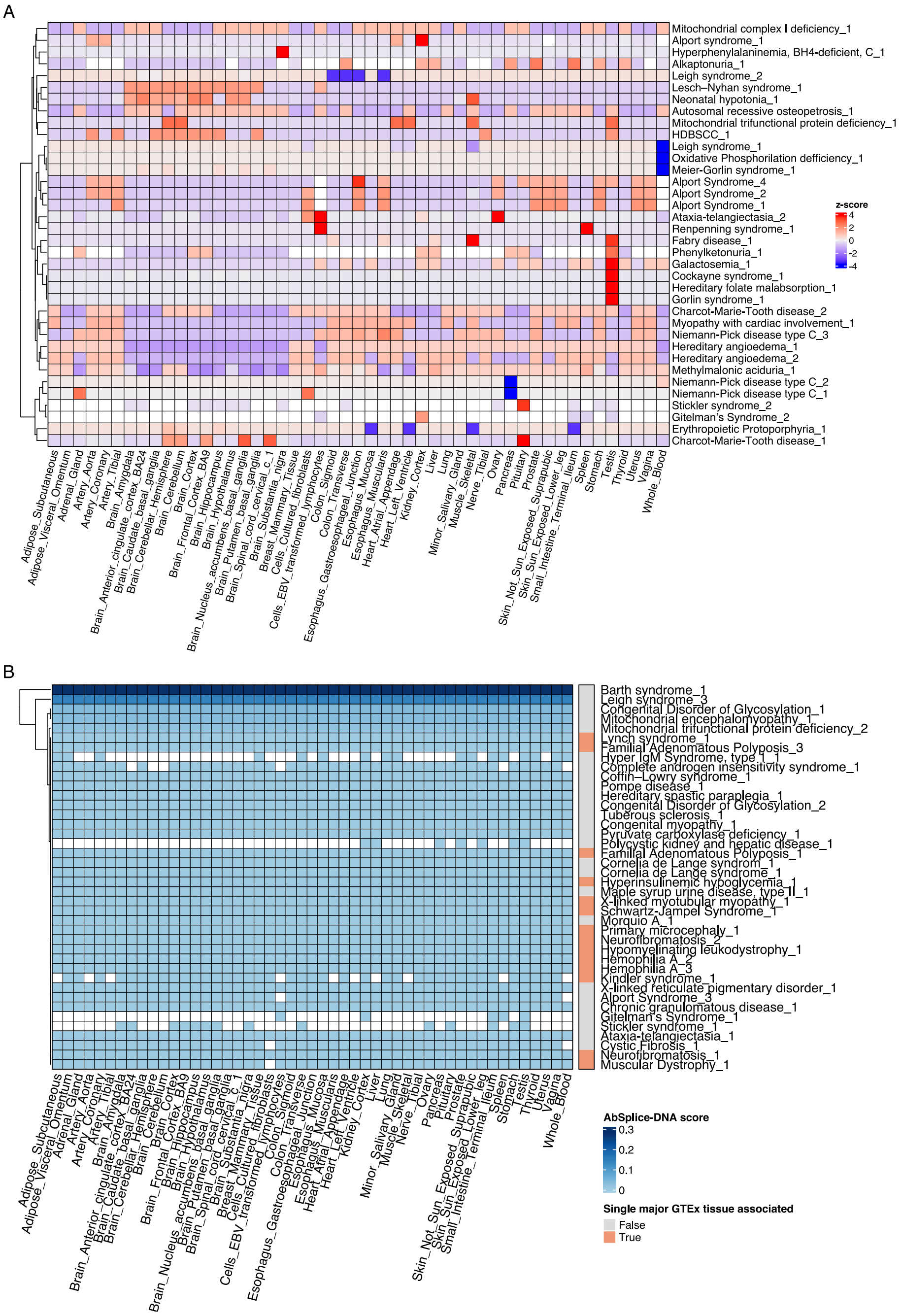

Supplementary Figure S 6. Tissue-specific predictions made by AbSplice-DNA for a set of disease-causing variants associated with aberrant splicing. **A** - Disease variants associated with multiple GTEx tissues that displayed variable scores across tissues. **B** - Disease variants with no tissue-specificity. All tissues got the same AbSplice-DNA score. Disease variants associated with one or more GTEx tissues are displayed in a single heatmap annotation.

Click here to access/download;Figure;plots_fig_clinvar.pdf

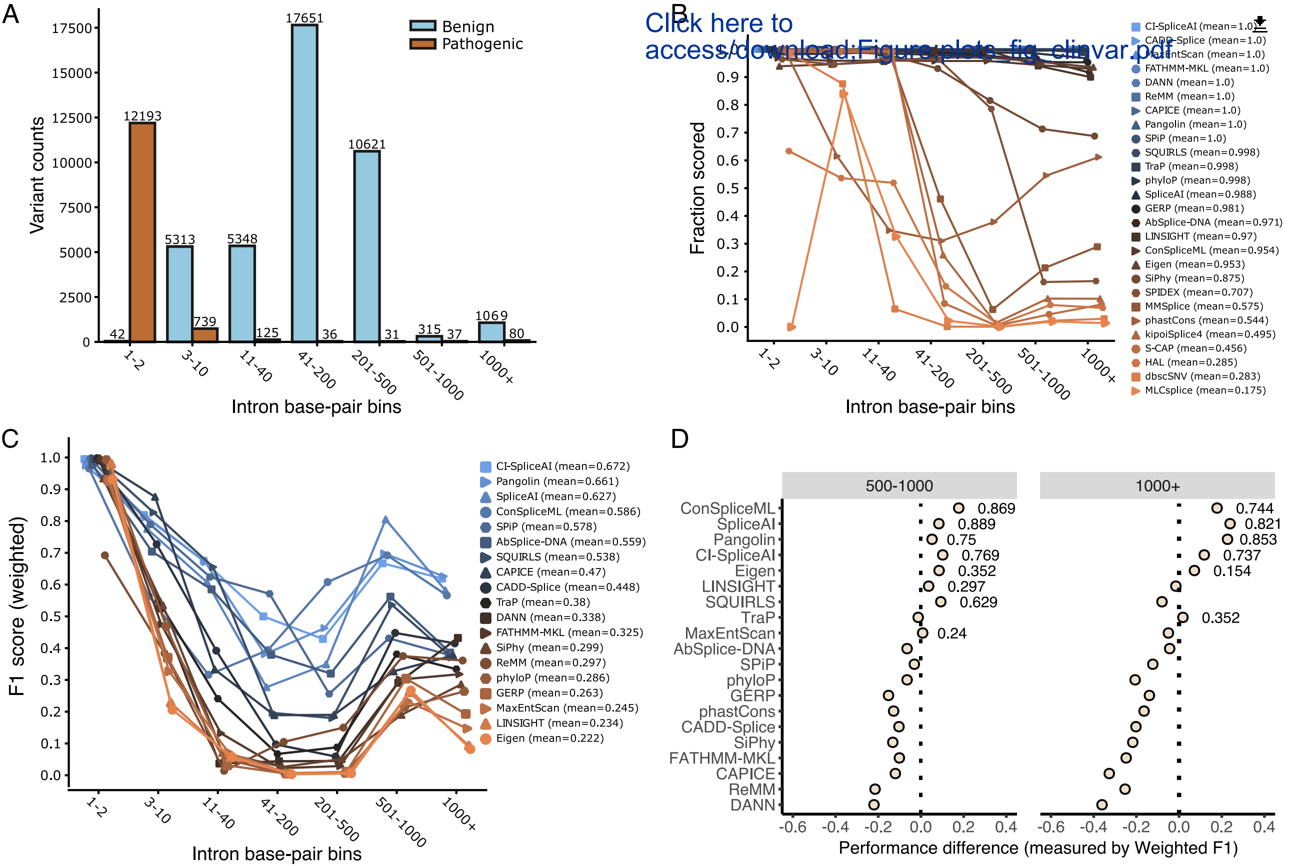

Click here to access/download;Figure;plots_fig_interpretability_and_usability.pdf

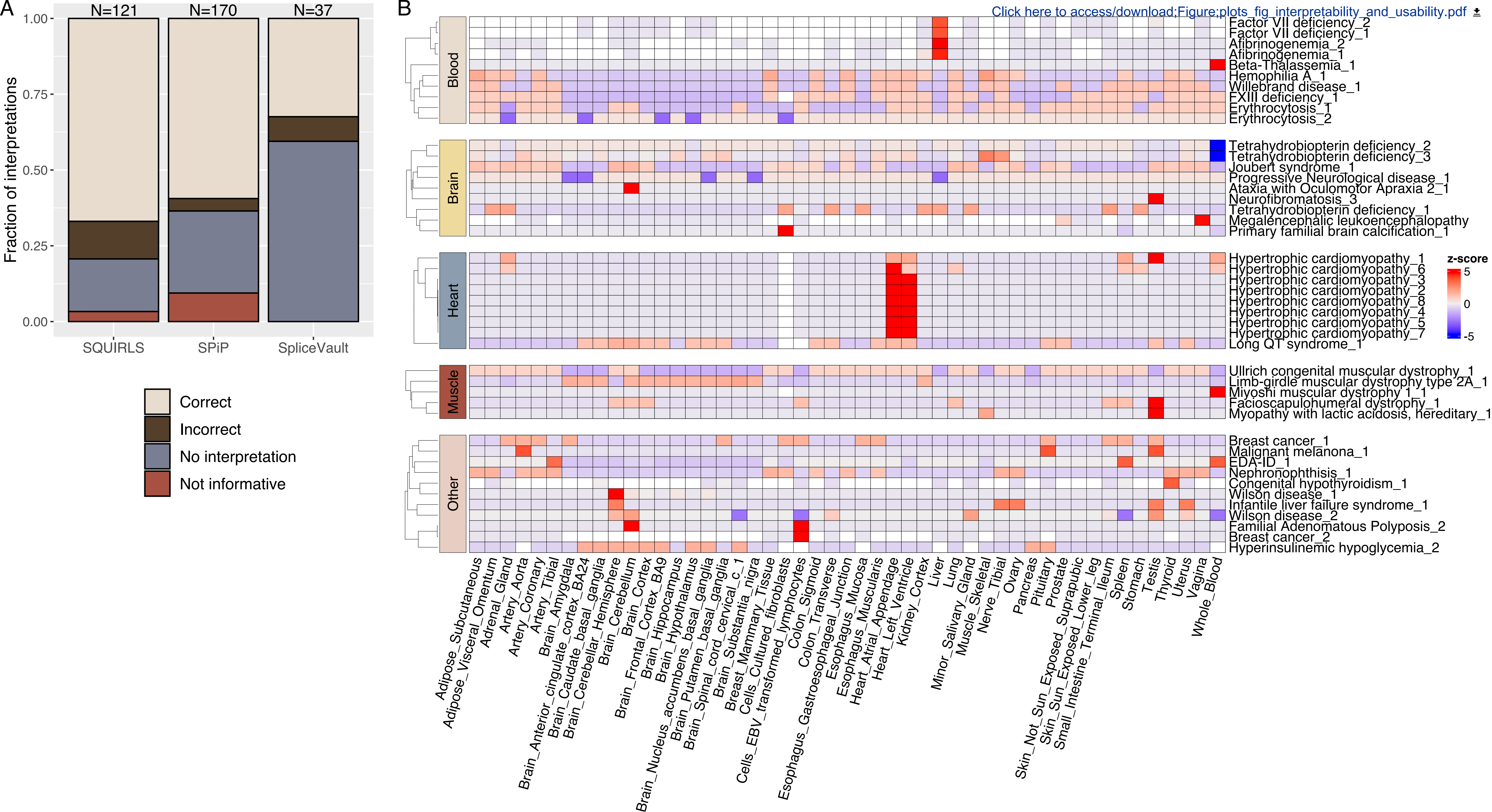

Click here to access/download;Figure;plots_fig_manual_curated.pdf

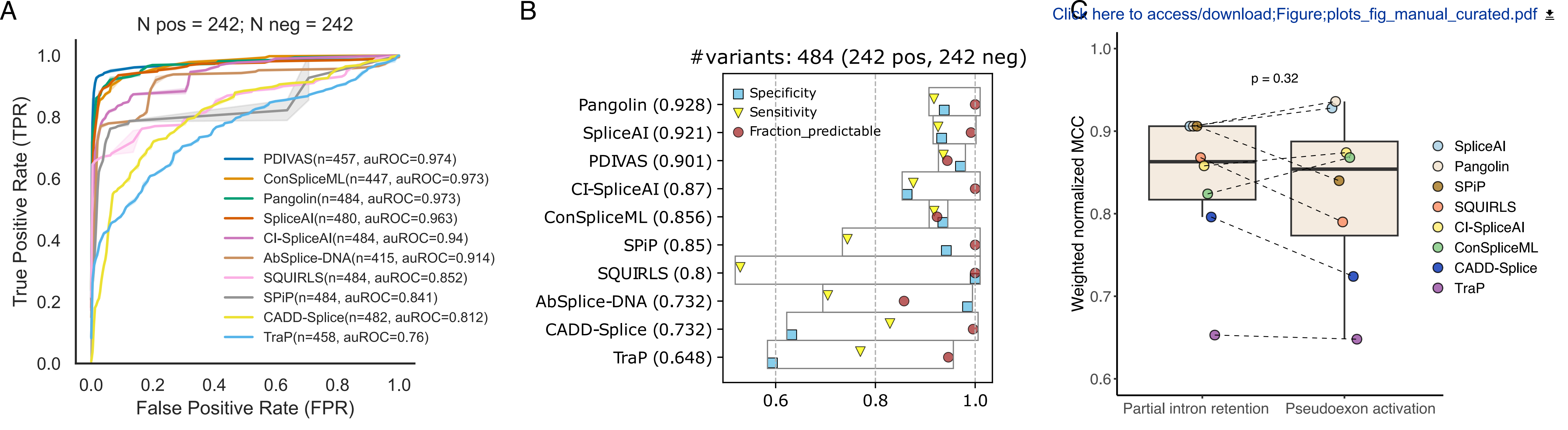

Click here to access/download;Figure;plots_fig_splicing_altering-eps-converted-to.pdf

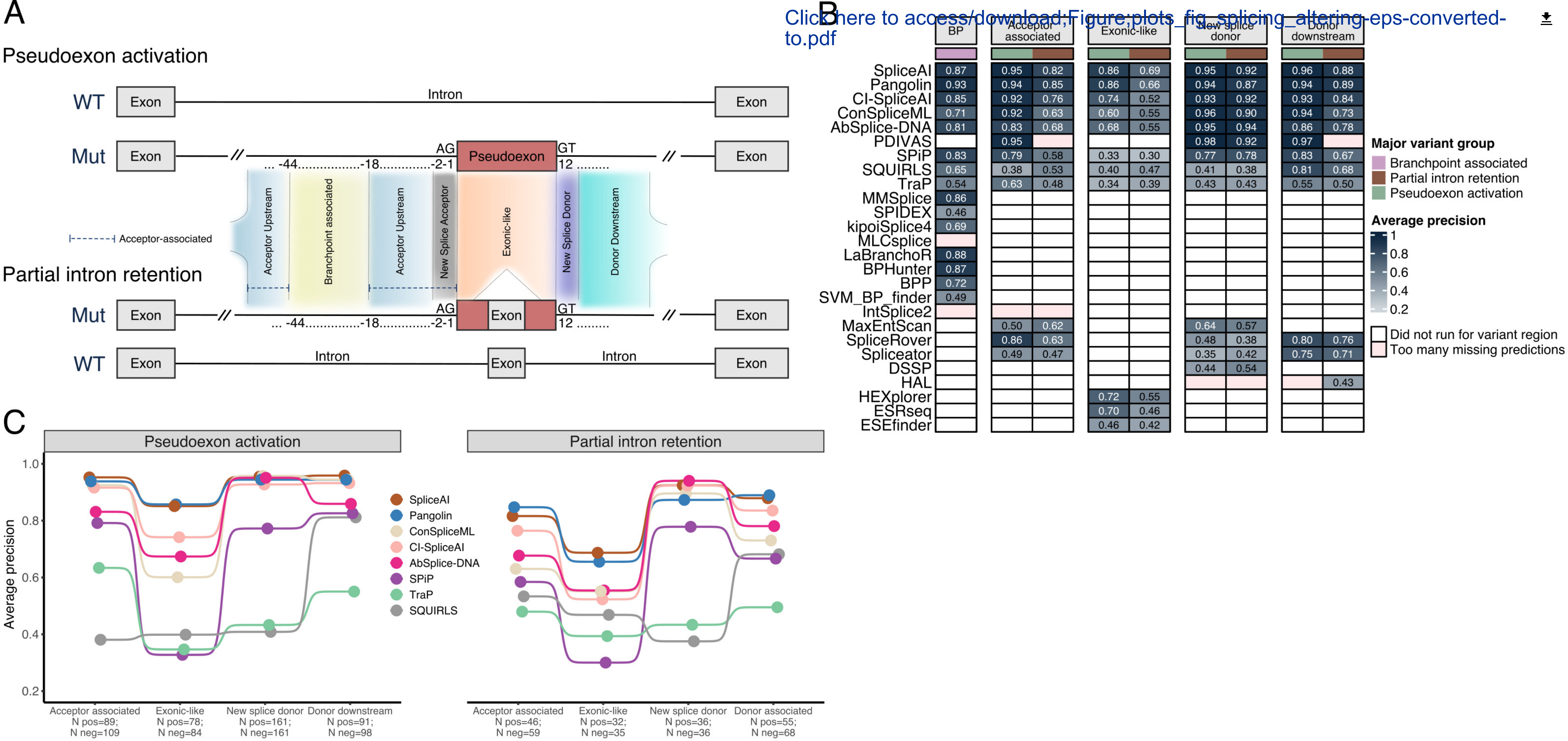

Click here to access/download;Figure;plots_fig_splicing_altering.eps

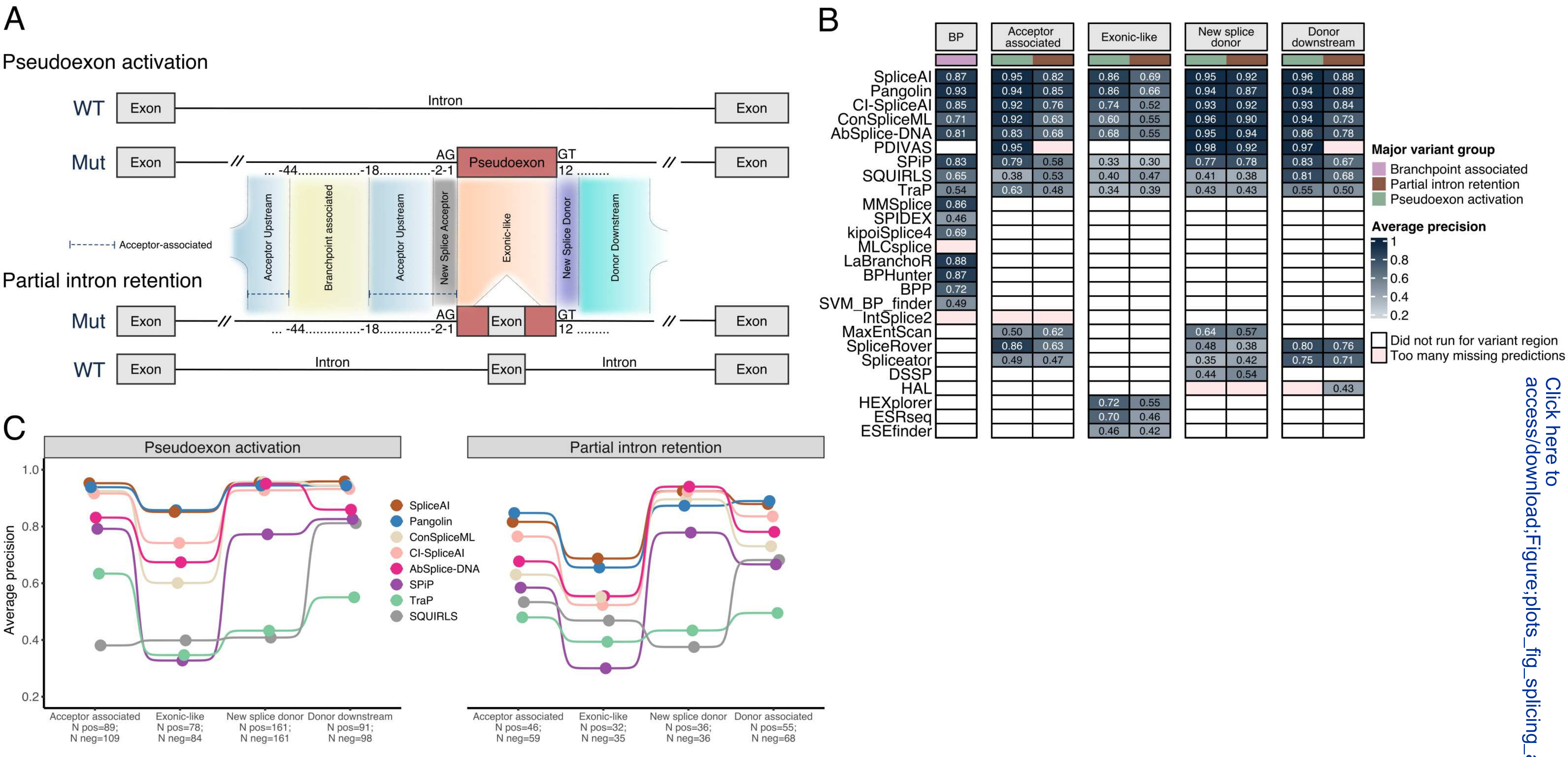

Click here to access/download;Figure;plots_fig_splicing_altering.pdf

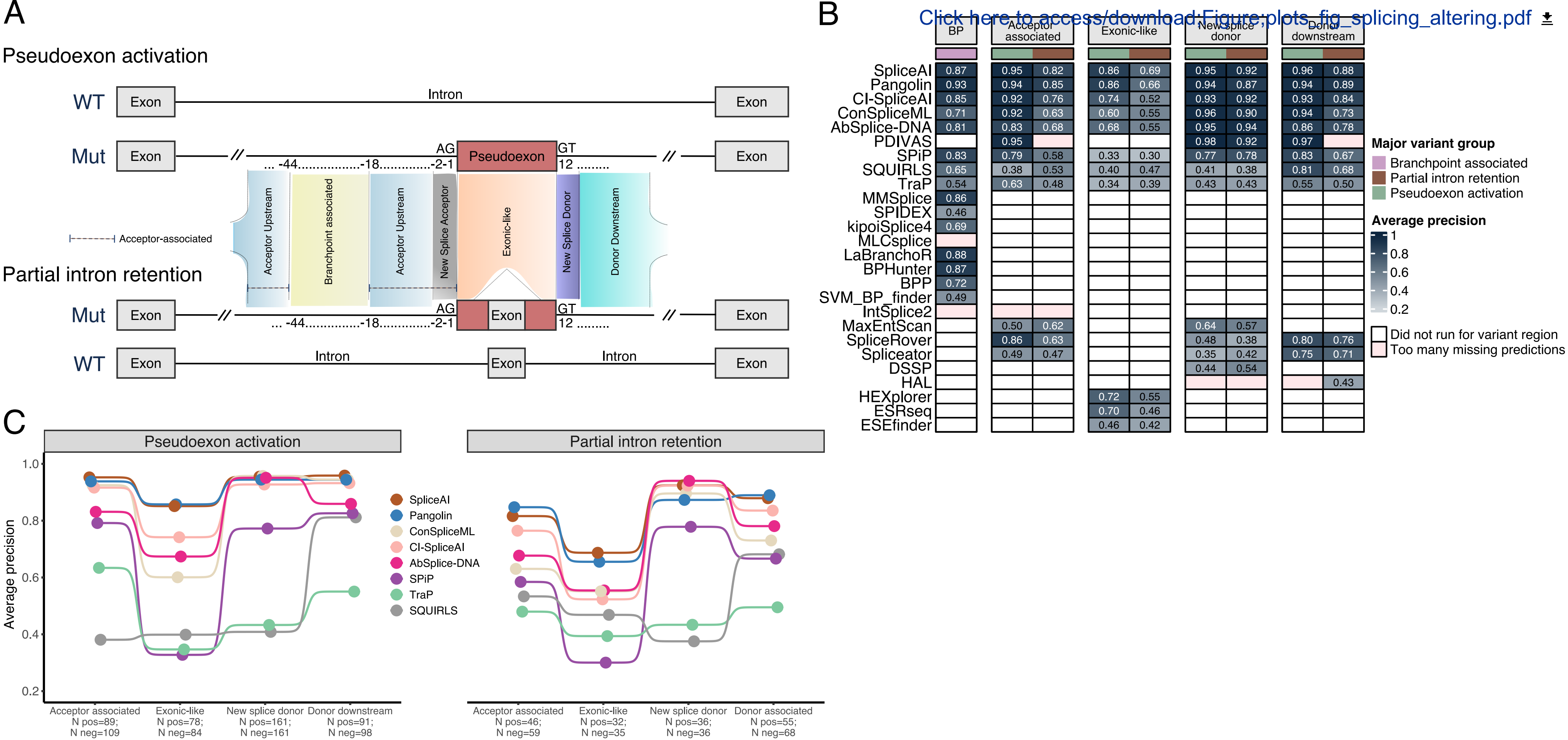

Click here to access/download
Supplementary Material
main.tex

Click here to access/download
Supplementary Material
oup-contemporary.cls

Click here to access/download
Supplementary Material
paper_alcides.sty

Click here to access/download
Supplementary Material
refs.bib

Click here to access/download
Supplementary Material
gigascience-logo.pdf

Click here to access/download
Supplementary Material
oup.pdf

Click here to access/download
Supplementary Material
plots_supp_fig_manual_curated.pdf

Click here to access/download
Supplementary Material
vancouver-authoryear.bst

Click here to access/download
Supplementary Material
plots_fig_splicing_altering.svg

Click here to access/download
Supplementary Material
plots_figure_1_clinvar.svg

Click here to access/download
Supplementary Material
plots_figure_2_splicing_pathogenic.svg

Click here to access/download
Supplementary Material
plots_figure_3_splicing_altering.svg

Click here to access/download
Supplementary Material
plots_figure_4_interpretability_tissue_specificity.svg

Click here to access/download
Supplementary Material
plots_supp_figure_S1_clinvar.svg

Click here to access/download
Supplementary Material
plots_supp_figure_S2_splicing_pathogenic.svg

Click here to access/download
Supplementary Material
plots_supp_figure_S3_splicing_altering.svg

Click here to access/download
Supplementary Material
plots_supp_figure_S4_pseudoexon_vs_partial_ir.svg

Click here to access/download
Supplementary Material
plots_supp_figure_S5_interpretability.svg

Click here to access/download
Supplementary Material
plots_supp_figure_S6_tissue_specificity.svg

Click here to access/download
Supplementary Material
table_S1_clinvar_data.tsv

Click here to access/download
Supplementary Material
table_S2_clinvar_data_results.tsv

Click here to access/download
Supplementary Material
table_S3_splicing_pathogenic_curation.tsv

Click here to access/download
Supplementary Material
table_S4_splicing_pathogenic_curation_results.tsv

Click here to access/download
Supplementary Material
table_S5_adjusted_tool_thresholds.tsv

Click here to access/download
Supplementary Material
table_S6_splicing_per_region_datasets.tsv

Click here to access/download
Supplementary Material
table_S8_spliceAI_manual_lookup.tsv

Click here to access/download
Supplementary Material
table_S7_splicing_per_region_datasets_results.tsv

Click here to access/download
Supplementary Material
table_S9_SpliceVault_presults.tsv

Click here to access/download
Supplementary Material
table_S10_AbSplice_DNA_tissue_assignments.tsv

Click here to access/download
Supplementary Material
plots_supp_fig_absplice.pdf

Click here to access/download
Supplementary Material
plots_supp_fig_clinvar.pdf

Click here to access/download
Supplementary Material
plots_supp_fig_pseudoexon_vs_partial_ir.pdf

Click here to access/download
Supplementary Material
plots_supp_fig_splicing_altering_prROCs.pdf

Click here to access/download
Supplementary Material
plots_supp_fig_interpretability.pdf

Click here to access/download
Supplementary Material
supplementary_tables_supplementary_tables.xlsx

Click here to access/download;Personal Cover;submission_reviews_giga_science_2nd_round.docx

Reviewer #1:
The authors have responded to all my comments and they have provided a solid update of their manuscript and results. I thank them, as their revisions extend beyond the specific points I raised. The quality and scope of this work have still increased, and I praise the authors' initiative to include one recently published predictor.

Minor point: I am very sorry to annoy again the authors with NTRK1. I truly hope my final comment won't delay anything as I think this variant won't make any significant difference.
That being said, I can't see how NTRK1 NM_001007792.1:c.851-798C>T and NTRK1 ENST00000524377.1:c.851-708C>T could be equivalent. According to VEP (https://rest.ensembl.org/vep/human/region/1:156872925/T?/1&mane=1/1&merged=1/1&hgvs=1), the variant chr1(hg38)-156872925-C-T has indeed the ENST00000524377.7:c.851-708C>T nomenclature, but also the NM_001007792.1:c.761-708C>T nomenclature (not c.851-798C>T). Moreover, the keegan table S1 displays a genomic region chr1(hg38):156872677-156872858, which seems incompatible with the variant 1:156872925. Finally, the keegan variant seems to be 2 substitutions, not one.

We are really grateful for the reviewer's feedback. About the specific variant that was mentioned, the reviewer is correct. Yet, there are no problems with the datasets, the variant is properly reported in the latest version of Table S6, which indeed displays the correct c.851-798C>T nomenclature as well as the hg38 position.

The problem was that we answered the NTRK1 issue before re-inspecting the whole datasets, so we forgot to update the answer in the document accordingly. The datasets and analyzes are fine, we apologize for the oversight.

Nevertheless, the reviewer raised an important point. The original paper reports the phenotype as having two substitutions occurring simultaneously, which we do not test in our experiments. One variant creates the splice site motif (c.851-794) and the other occurs 4 base-pairs upstream, within the pseudoexon (c.851-798). Because it does not seem plausible that the pseudoexon would be activated by the c.851-798 alone, we have removed that variant from the dataset and reanalyzed the section accordingly. As a sanity check, when we run SpliceAI with just the c.851-798 variant, the splice site score for the donor position is 0. When we run SpliceAI with just the c.851-794 variant the score is 0.59. Lastly, running the sequence with both c.851-794 and c.851-798 returns a score of 0.64, suggesting that the c.851-798 variant slightly enhances the splice site generated by c.851-794.

Reviewer #2:

I thank the authors for this review. Overall, the authors successfully addressed my comments and the modified manuscript is more precise and clearer. I hence strongly support its publication. I have few minor comments:

- According to my comment #1 of the first review, the authors corrected the set of ClinVar data to remove the training variants of CAPICE tool. Consequently, the

performance of CAPICE was largely reduced with a top-ranking of 8 against 4 in the initial version. Interestingly, the new performance of CAPICE was in line with other tools that used ClinVar data in their development (ex: SQUIRLS, 7th-top ranking or CADD-splice, 9th-top ranking). I agree with the discussion of the authors, the use of ClinVar data did not seem to induce a major bias as the best tools of this study were not the tools using ClinVar data in their training.

We're glad to see that the reviewer found the alterations we made to the paper useful.

- About my comment #6 of the first review, I can understand that my explanation was not enough clear. I think is better if I take some examples. In *BRCA2* gene (NM_000059) the variant c.682-2A>C led to, as expected for a canonical variant, an exon skipping but the major aberrant splicing event was the use of an acceptor splice site at 73 nt downstream of the natural splice site (http://dx.doi.org/10.1016/j.jmoldx.2014.01.005). In *NF1* gene (NM_000267) the variant c.204+1G>A led to the use of a donor splice site at 104 nt upstream of the natural splice site (http://dx.doi.org/10.1002/humu.20826). In both two case, the new sites do not seem to be impact by the variant and their use is not predicting by the tools, ex: SpliceAI: c.682-2A>C AL: 1.00 (2); AG: 0.11 (13) and c.204+1G>A DL: 1.00 (-1); DG: 0.02 (-46). It would be reasonable to think that the authors have similar variants in their collection of variants.

We thank the reviewer for further clarification on this point. It is indeed reasonable to think that some variants possibly display such an outcome. However, as far as we remember from the curation procedure, we do not recall any particular case with validation studies that report such a case. As most of the variants trigger pseudoexon inclusion (hence activate something), we would argue that it is less likely to happen than with a splice site that breaks a canonical exon (disrupts something). The variants where it does seem more likely to happen were those 6 branchpoint variants that triggered exon skipping and partial intron retention.

Computationally, we think SpliceAI can capture such outcomes if we increase the max distance parameter, as we recommend in the discussion. When running those two variants with SpliceAI lookup with max distance of 500, SpliceAI gives higher scores for the gains (0.3 for BRCA2, 0.72 for NF1).
