# Supplementary material for: Computational prediction of human deep intronic variation
Source: Gigascience. 2023 Oct 25;12:giad085. doi: 10.1093/gigascience/giad085 (PMC10599398; doi:10.1093/gigascience/giad085)
Supplement: giad085_Supplemental_Files [file giad085_supplemental_files.zip › Additional file1.docx]

Tables

**Table S1:** A summary of the ClinVar variants used in the study, including the intronic bins they were assigned to.

**Table S2:** Performance statistics at each intronic bin.

**Table S3:** Pathogenic splicing-altering variants curated for this study (it includes vazDrago et.al, 2017 variants).

**Table S4:** Performance statistics for the manual curated dataset of splicing pathogenic mutations (this study plus Vaz-Drago et al. 2017).

**Table S5:** Optimized thresholds that maximize performance for non-canonical intronic splicing variants at different levels of importance given to precision and recall.

**Table S6:** Multiple splicing-associated datasets evaluated in this study.

**Table S7:** Performance evaluation results for all variants associated with splicing alterations.

**Table S8:** SpliceAI lookup output (with max distance = 500) for the Exonic-like splicing-altering variants that were missed by SpliceAI using pre-computed scores.

**Table S9:** SpliceVault output for variants used in the analysis.

**Table S10:** Data used to analyse tissue-specificity of AbSplice-DNA.

Figures

**Supplementary Fig. S1:** Intronic variant prediction in ClinVar. **A -** Performance of all tools considered for the study on the raw ClinVar intronic dataset. Mean values in the legend represent the average weighted F1 score across all intronic bins. **B -** Inspection of intronic variants (after addressing circularity problems) assigned to the “501-1000” and “1000+” intronic bins. The bars reflect the number of variants assigned to each category. The term “No other transcript” refers to all variants that do not have any other RefSeq protein coding transcript of the same gene overlapping with them, besides the transcript originally used (N pathogenic=25, N benign=340). “> 1 transcript (same offset)” refers to variants that overlap with more than one transcript of the same gene but do not have any other transcript where the variant is closer to the splice site than in the original transcript used in the analysis (N pathogenic=31, N benign=527). “> 1 transcript (smaller offset)” refers to variants that overlap with more than one transcript of the same gene, and have at least one other transcript in which the variant is closer to the splice site than in the original transcript used in the analysis (N pathogenic=20, N benign=254). “Exonic” refers to variants that overlap with more than one transcript of the same gene, and have at least one other transcript where the variant is exonic (N pathogenic=41, N benign=263). **C -** Distribution of the updated intronic distances to the closest splice site for variants assigned to the “> 1 transcript (smaller offset)” category. **D -** Tool performance (measured with weighted F1 score) for each individual category. Tools with performance higher than 0.6 are highlighted.

**Supplementary Fig. S2:** Manually curated dataset of pathogenic intronic variants disrupting RNA splicing. **A -** Number of variants collected per phenotype. Diseases with less than 3 variants were assigned to the ’Other’ category. **B -** Number of variants occurring in ClinVar and gnomAD v2.1. **C -** Log transformed allele frequencies of variants in gnomAD v2.1. For those that are absent in the database, a pseudocount of 0.00001 was added (highest histogram peak, at 5). **D -** Tool performance using reference thresholds from Table 1 for variants curated in this manuscript plus those curated by Vaz Drago et al., 2017. MLCsplice and dbscSNV are not shown as they had more than 95% of missing predictions. **E -** Distance (Log10) of the variants to the closest splice junction. Pseudoexon activation group: 194 variants; Partial intron retention group: 37 variants.

**Supplementary Fig. S3:** Precision-Recall curves for all splicing-altering variants analyzed in a region-specific manner. Tools are ranked by the auPRC metric and the

number of predictions made by each tool is displayed in “n=”. The number of variants in each dataset is presented (“N pos” represents the number of positive splicing altering variants; “N neg” is the number of negative splicing variants). Tools with more than 50% of missing predictions or with less than 15 variants in the minority class were excluded from these analyses. For the Exonic-like category, PDIVAS was excluded because control variants in this comparison are exonic, which is outside of the scope of this model. **A -** Branchpoint associated variants. **B -** Acceptor-associated variants that trigger pseudoexon inclusion. **C -** Acceptor-associated variants that lead to partial intron retention. **D -** Exonic-like variants that trigger pseudoexon inclusion. **E -** Exonic-like variants that lead to partial intron retention. **F -** Variants that create new splice donors and activate pseudoexons. G - Variants that create new splice donors and lead to partial intron retention. **H -** Variants that activate existing upstream cryptic splice donors and trigger pseudoexon activation. I - Variants that activate existing upstream cryptic splice donors and lead to partial intron retention.

**Supplementary Fig. S4:** Comparing performance between all pseudoexon activation versus partial intron retention variants collected in this study. **A -** Distribution of the auPRC scores of the tools for each variant region. **B -** Per-tool auPRC distribution across the four variant regions considered. A Fisher’s exact test was conducted separately for each tool and variant region to determine statistical significance for the performance differences between the pseudoexon activation and partial intron retention groups. The true positives plus true negatives were considered successful outcomes, while false positives plus false negatives were considered failures. The p-values displayed in the figure were corrected for multiple comparisons using the Holm method. For each tool, we excluded the variant regions that did not have performance measurements in both groups.

**Supplementary Fig. S5:** Interpretability assessment. **A -** Distance of the variants to the activated cryptic splice site (relative to the first splice site position) in SpliceVault

analysis for the variants assigned to the "No interpretation" tag. **B -** Distribution of SPiP and SQUIRLS prediction values for each of the interpretation categories.

**Supplementary Fig. S6:** Tissue-specific predictions made by AbSplice-DNA for a set of disease-causing variants associated with aberrant splicing. **A -** Disease variants associated with multiple GTEx tissues that displayed variable scores across tissues. **B -** Disease variants with no tissue-specificity. All tissues got the same AbSplice-DNA

score. Disease variants associated with one or more GTEx tissues are displayed in a single heatmap annotation.
