# Supplementary figures and images for: Computational prediction of human deep intronic variation
Source: Gigascience. 2023 Oct 25;12:giad085. doi: 10.1093/gigascience/giad085 (PMC10599398; doi:10.1093/gigascience/giad085)

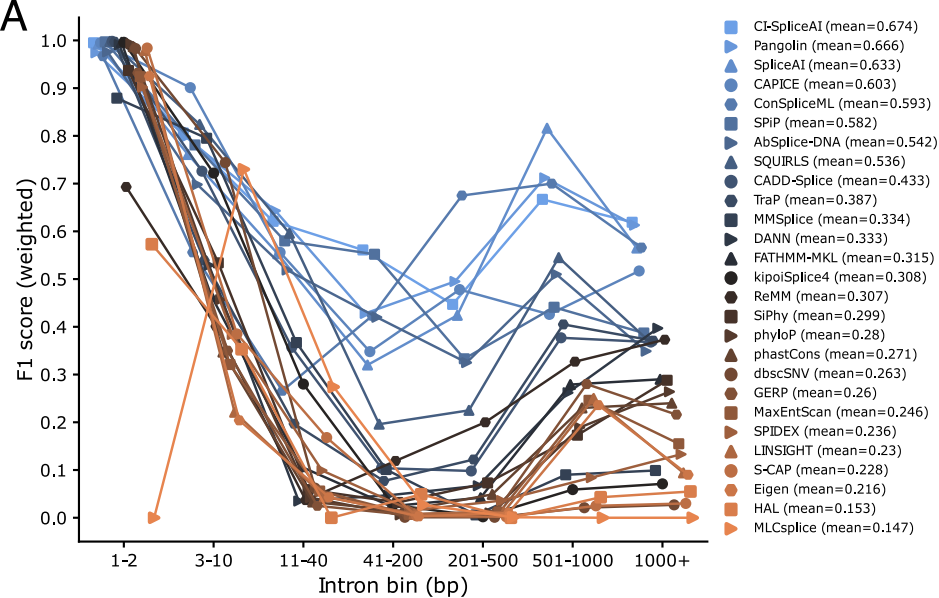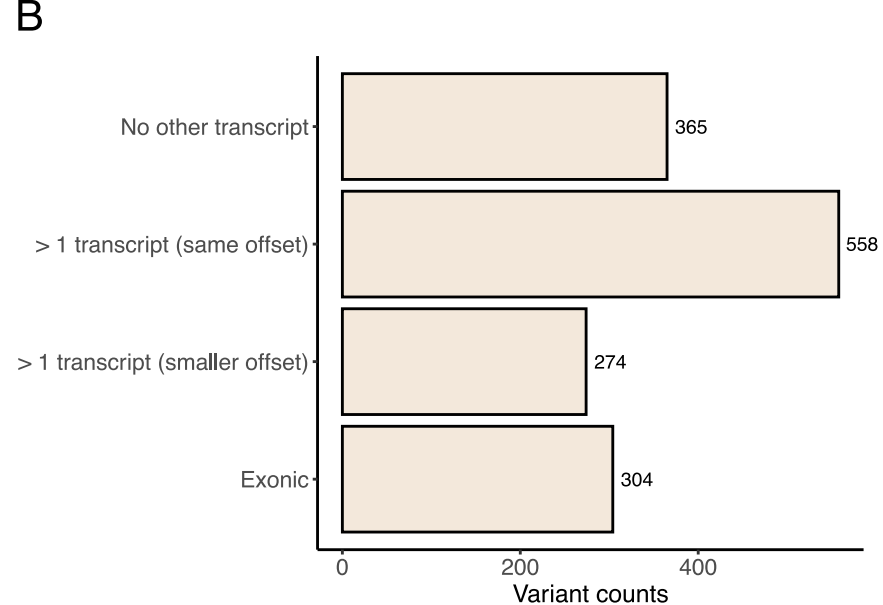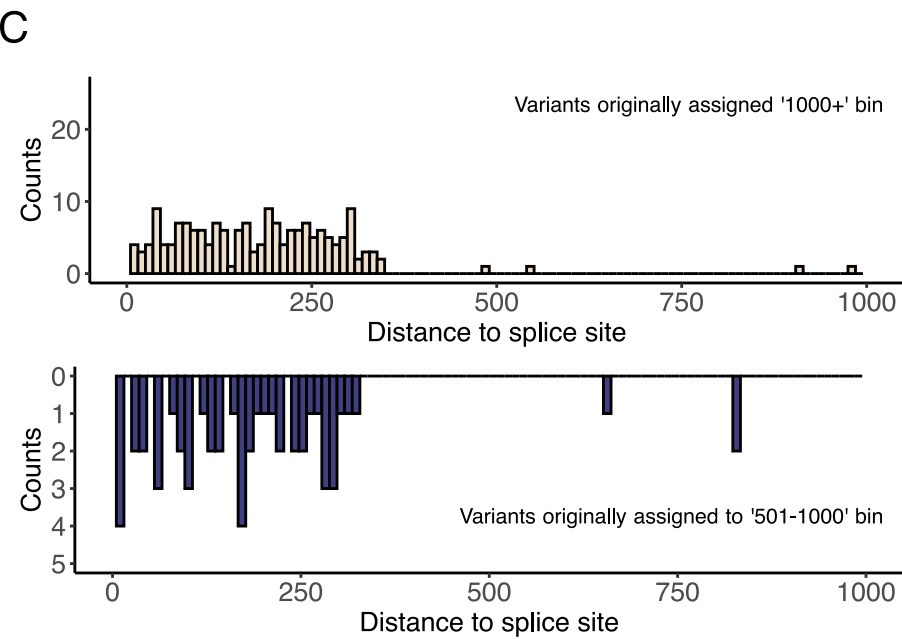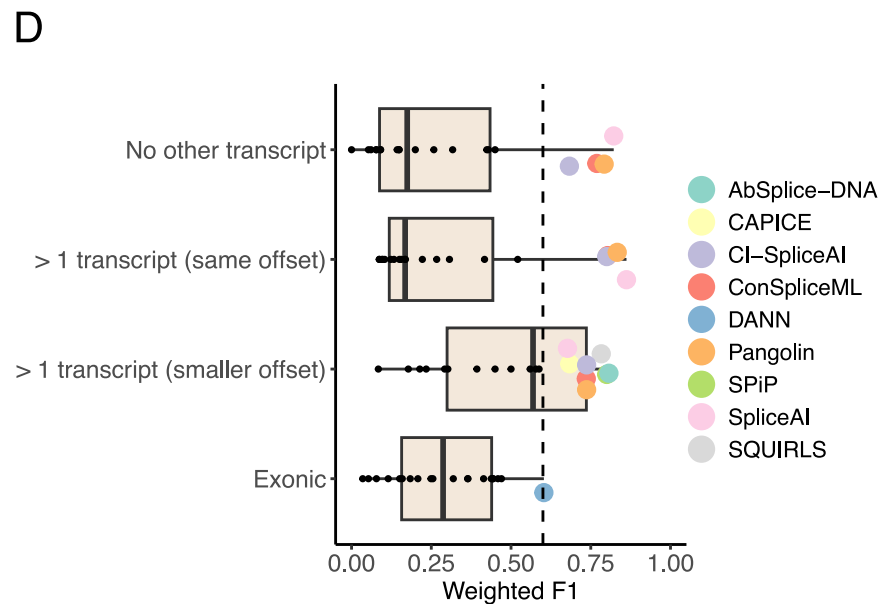

Supplement: giad085_Supplemental_Files [file giad085_supplemental_files.zip › figure_S1_supplementary_material.pdf]

A

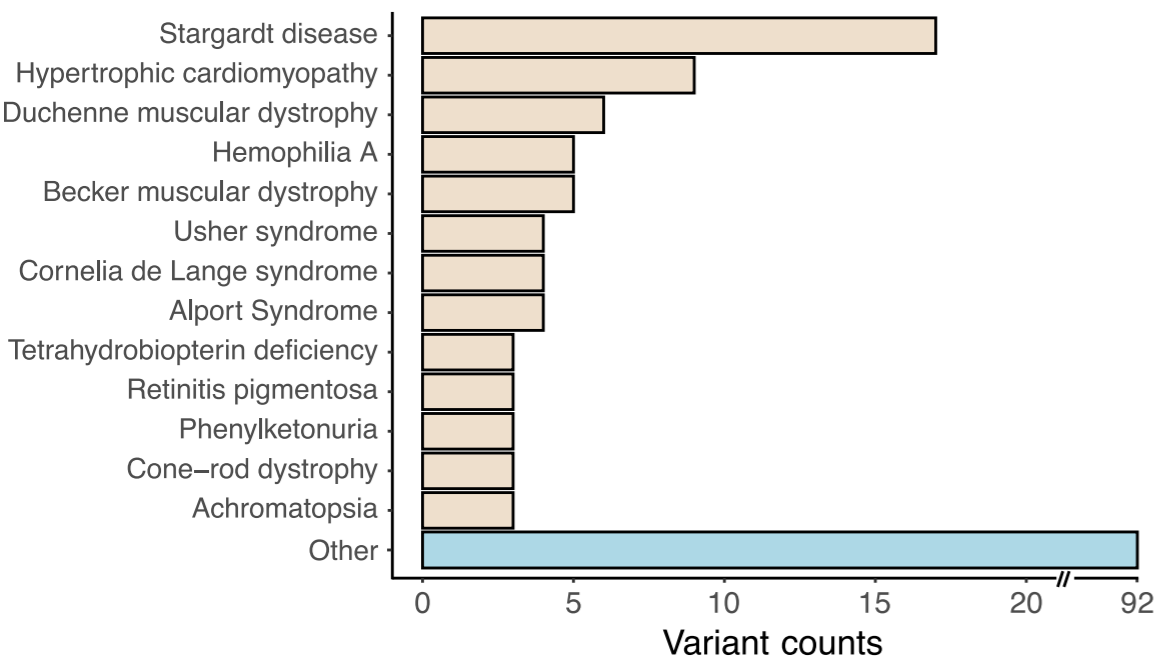

B

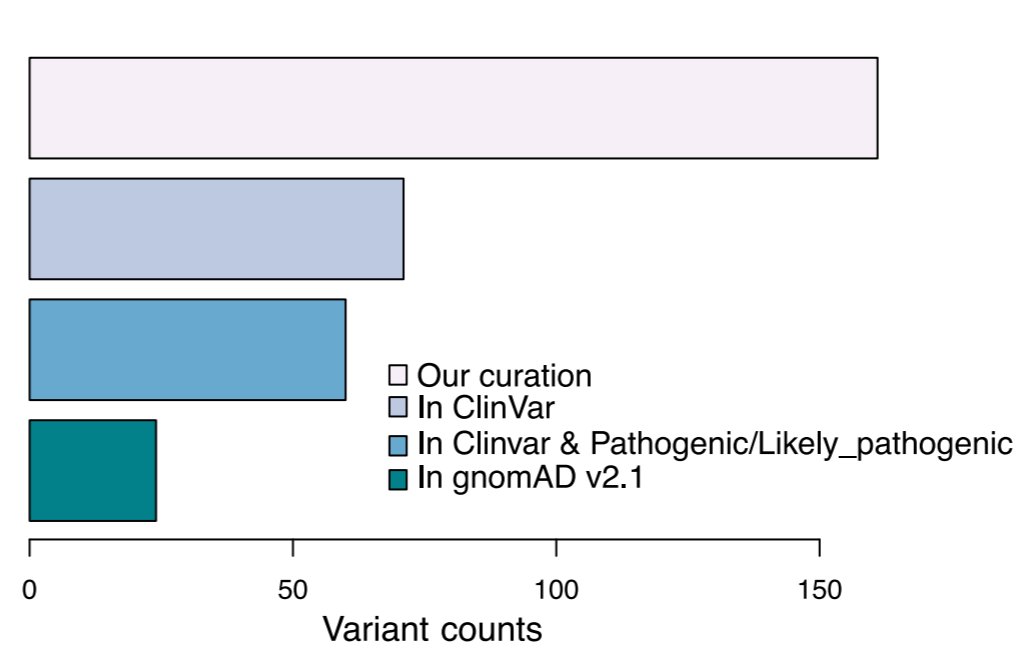

C

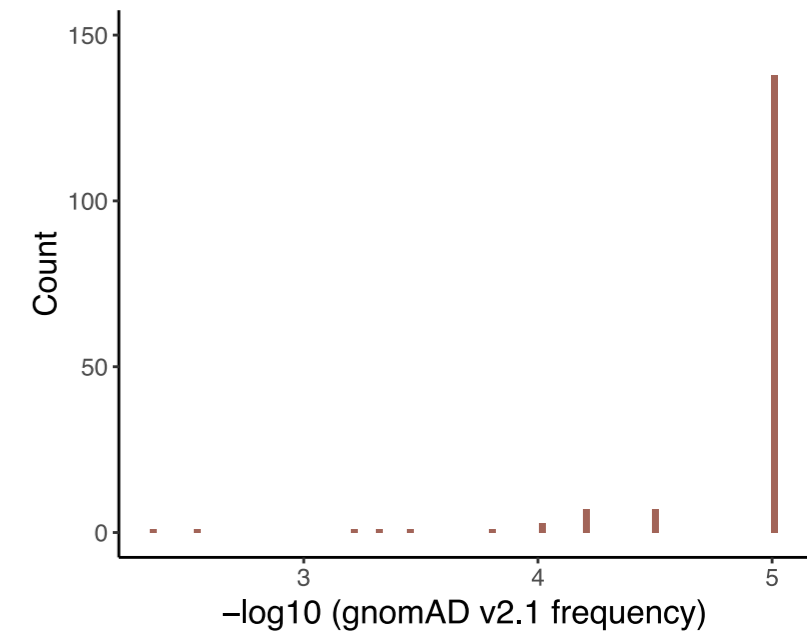

D

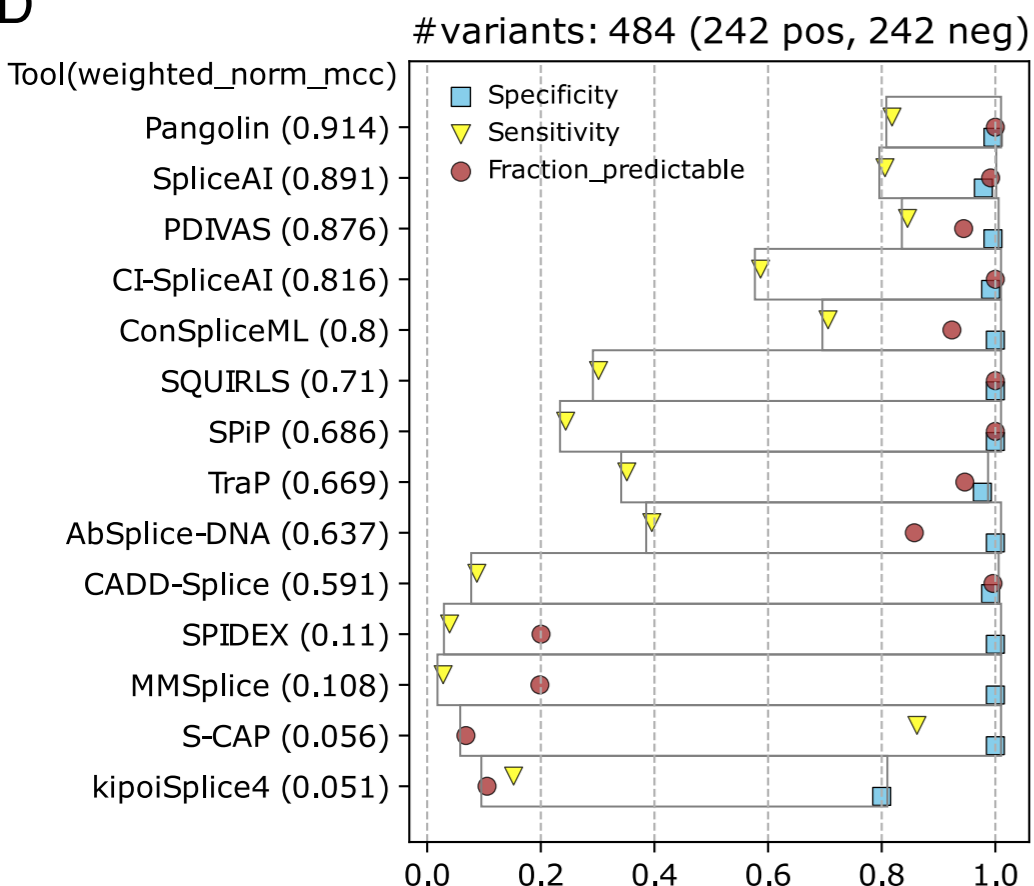

E

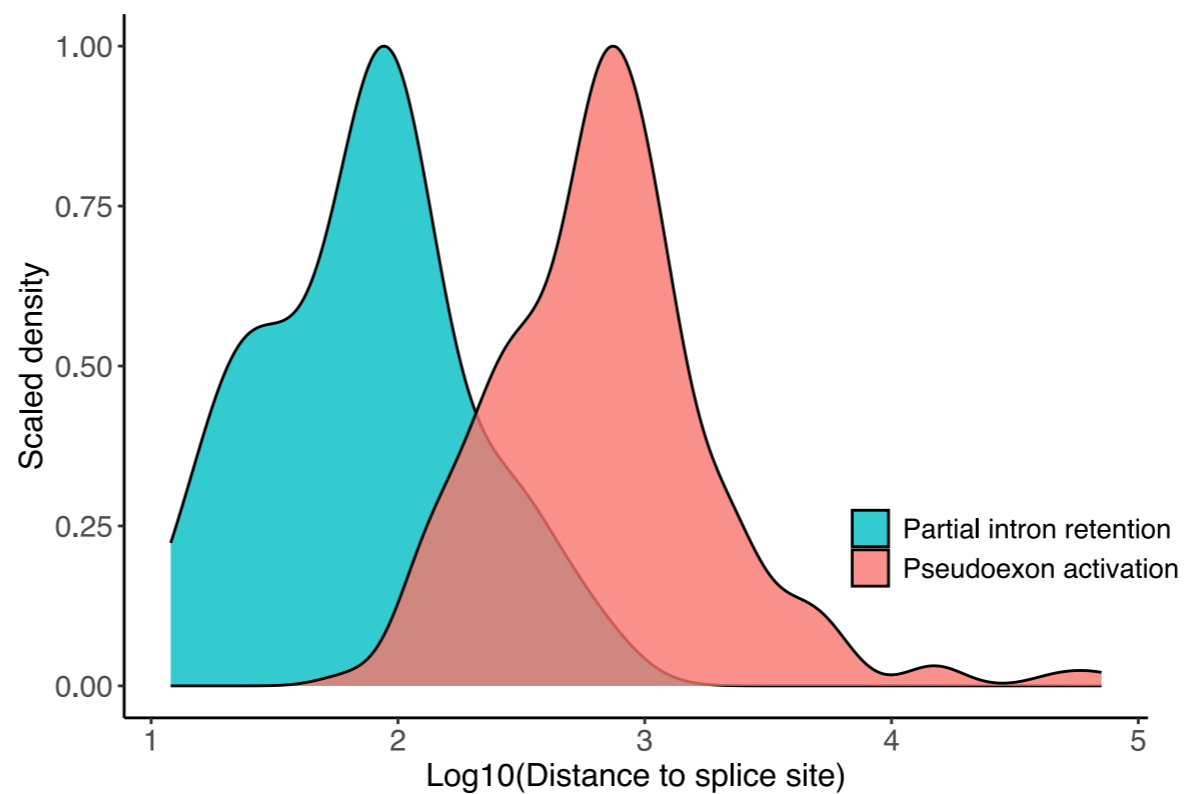

Supplement: giad085_Supplemental_Files [file giad085_supplemental_files.zip › figure_S2_supplementary_material.pdf]

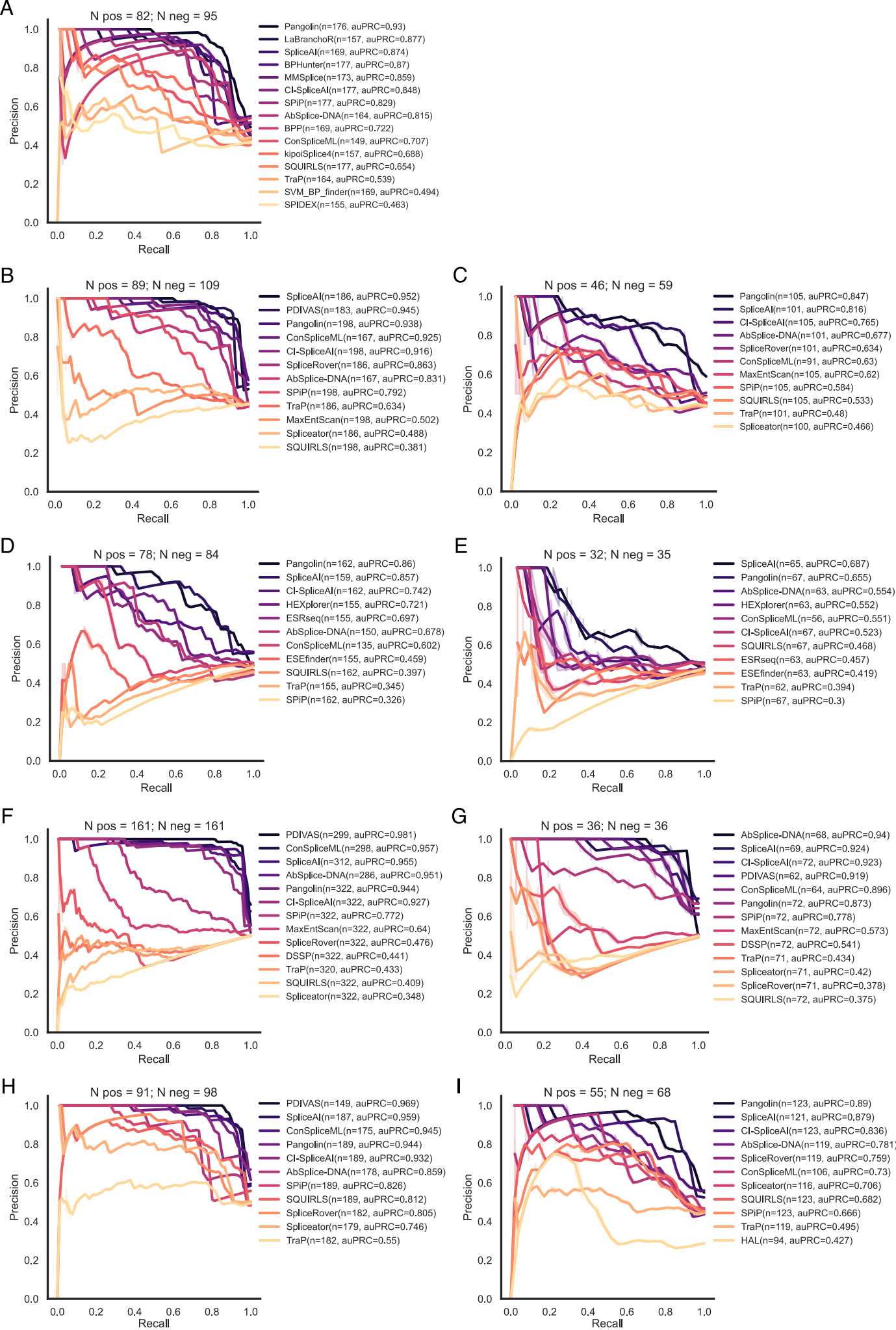

Supplement: giad085_Supplemental_Files [file giad085_supplemental_files.zip › figure_S3_supplementary_material.pdf]

A

auPRC

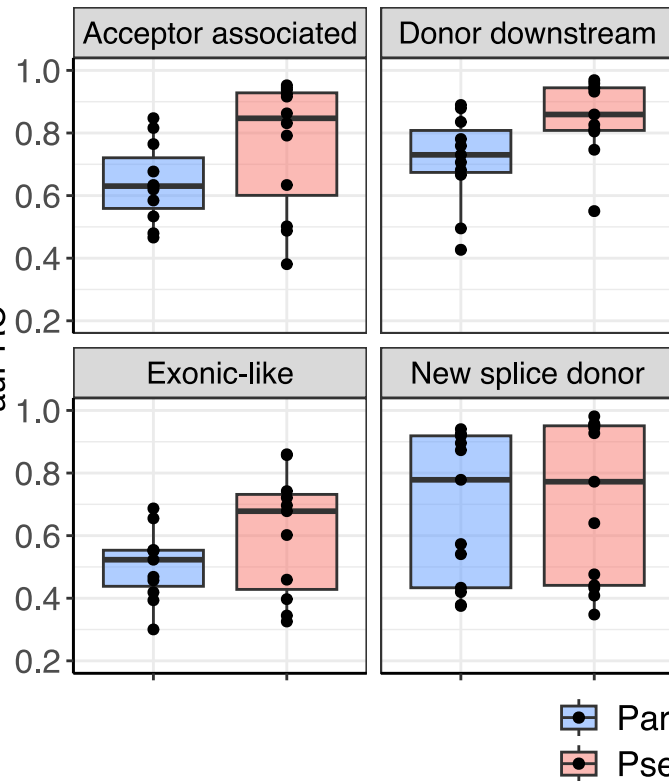

B

auPRC

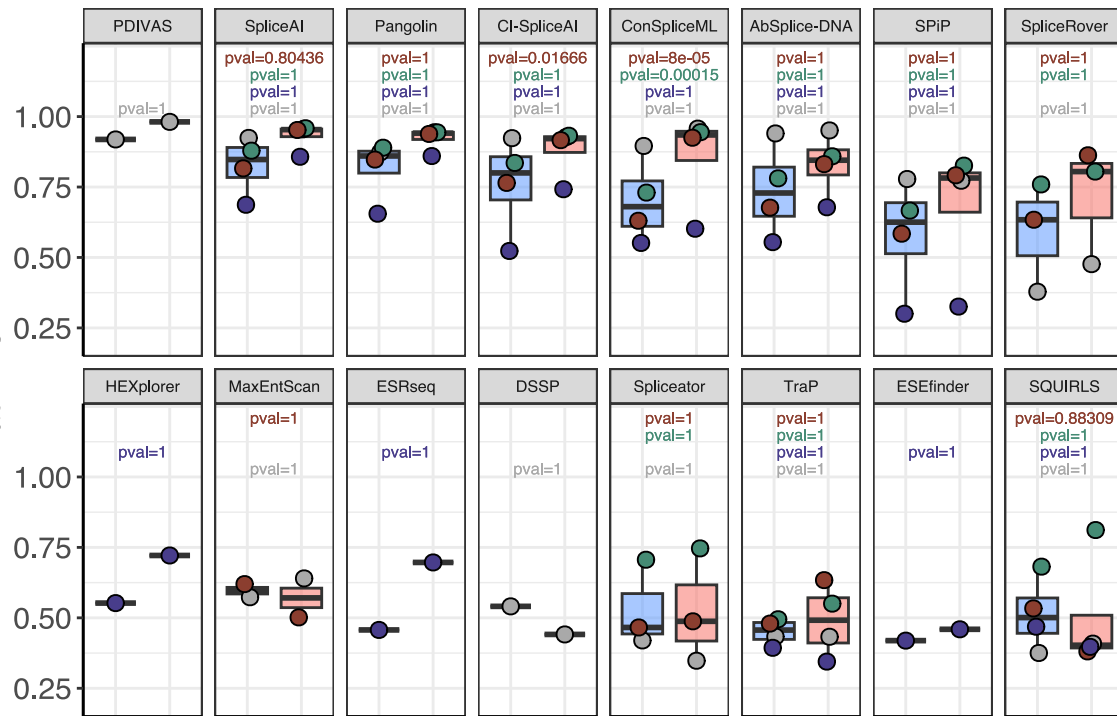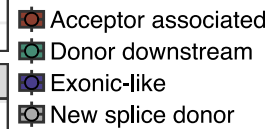

Supplement: giad085_Supplemental_Files [file giad085_supplemental_files.zip › figure_S4_supplementary_material.pdf]

A

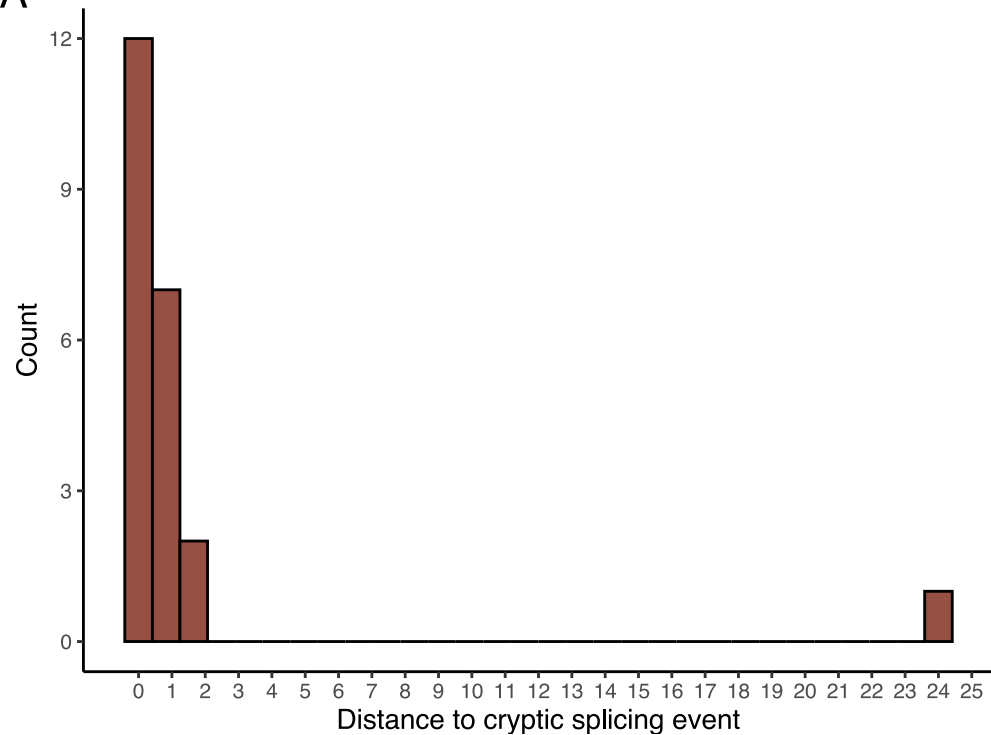

B

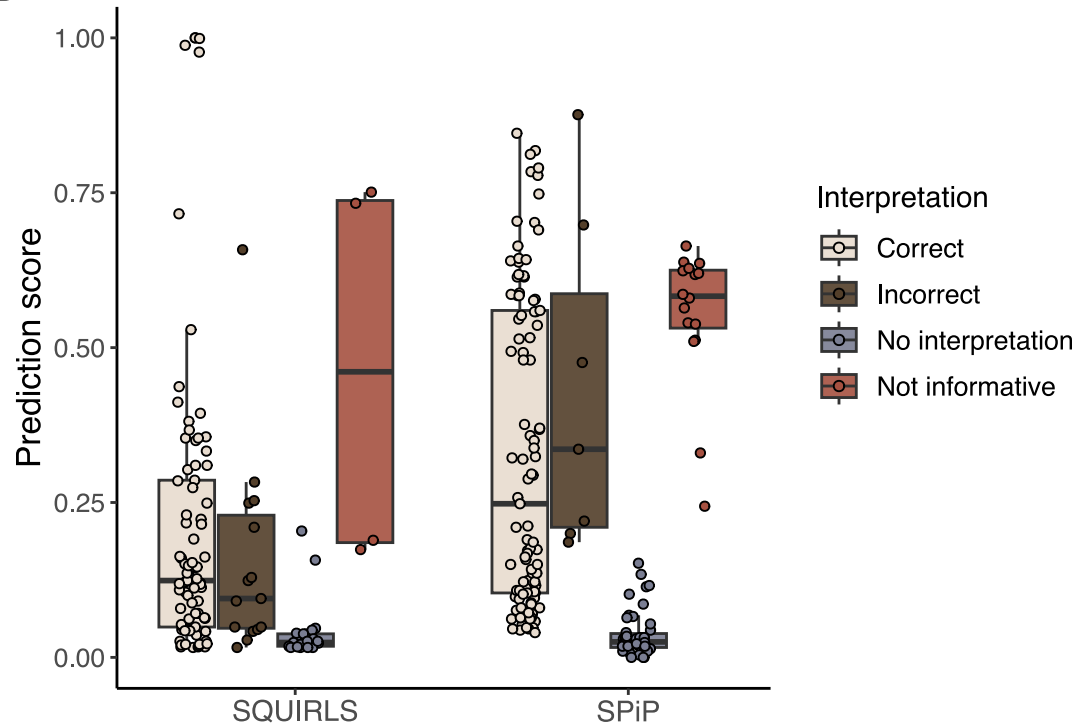

Supplement: giad085_Supplemental_Files [file giad085_supplemental_files.zip › figure_S5_supplementary_material.pdf]

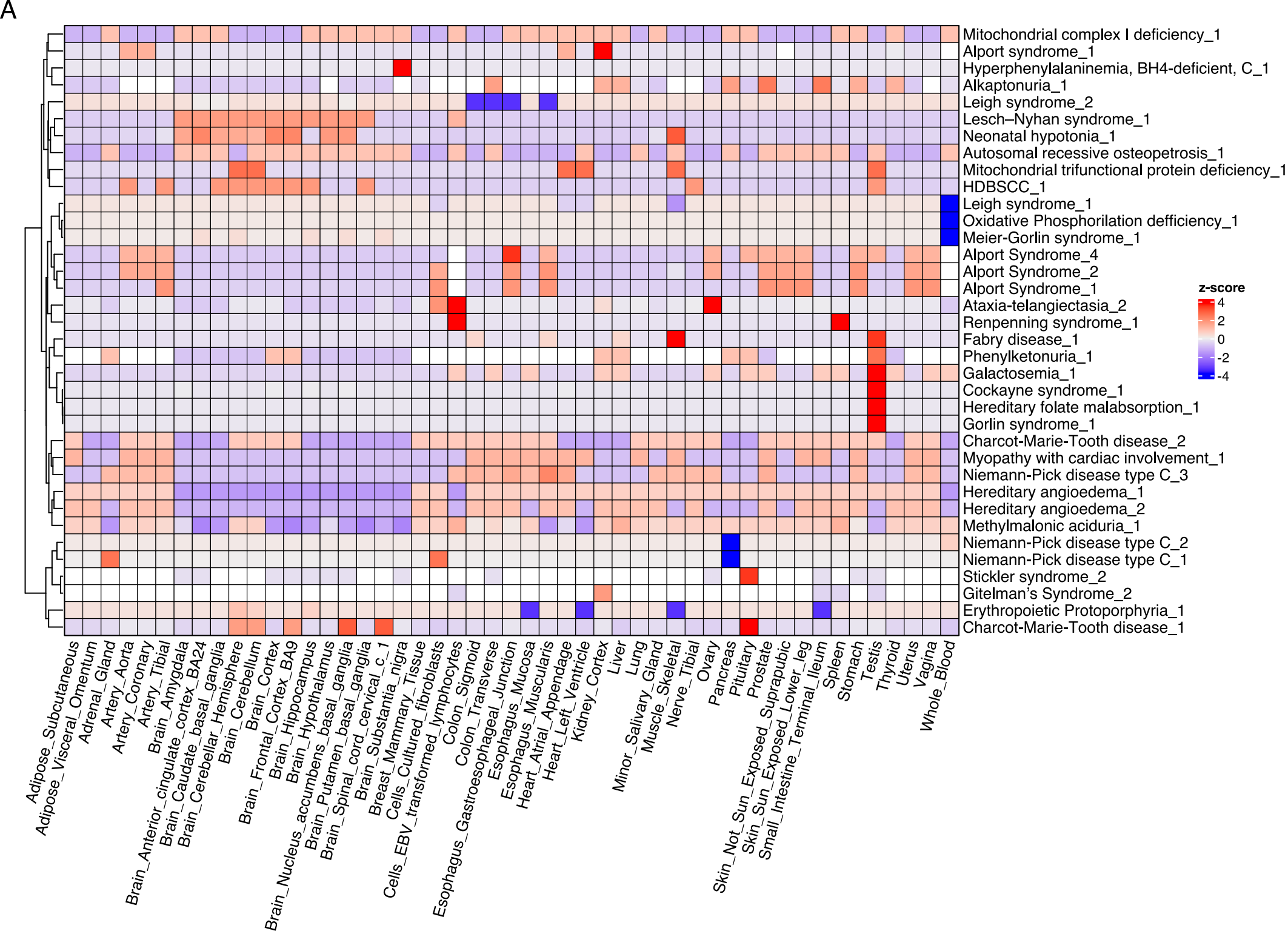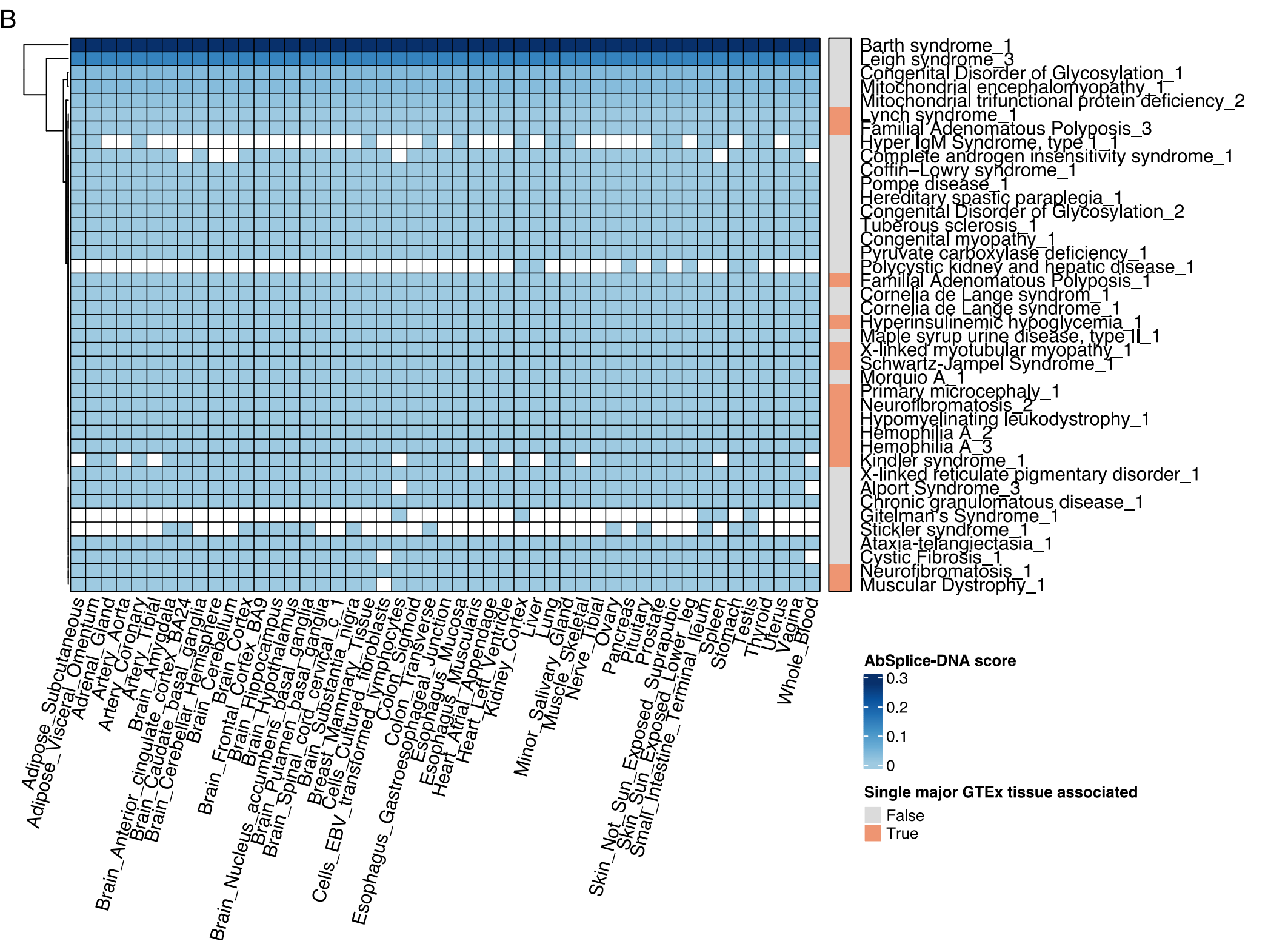

Supplement: giad085_Supplemental_Files [file giad085_supplemental_files.zip › figure_S6_supplementary_material.pdf]
