# Supplementary material for: Computational prediction of human deep intronic variation
Source: Gigascience. 2023 Oct 25;12:giad085. doi: 10.1093/gigascience/giad085 (PMC10599398; doi:10.1093/gigascience/giad085)
Supplement: giad085_Response_to_Reviewer_Comments_Original_Submission [file giad085_response_to_reviewer_comments_original_submission.pdf]

Author's Response To Reviewer Comments

Dear Editor and Reviewers,

We appreciate your effort and dedication in reviewing the previous version of this work. We have addressed your concerns in this new version that we are submitting, and we include a response_to_reviewers.docx, in which we detail how we addressed each of your feedback points individually. Due to your feedback, we manually reinspected the datasets to exclude and correct misclassified data. As such, the values reported in the paper might have slightly changed, but the overall results and conclusions remain the same. We have also improved the interpretability section by adding SpliceVault to the analysis. Additionally, we included a new tool (PDIVAS) that has been published in the meanwhile, that specifically targets deep intronic regions.

Overall, we thank the reviewers for the insightful feedback that we took to heart and we believe it vastly improved the manuscript to the current version.
