# Supplementary material for: Computational prediction of human deep intronic variation
Source: Gigascience. 2023 Oct 25;12:giad085. doi: 10.1093/gigascience/giad085 (PMC10599398; doi:10.1093/gigascience/giad085)
Supplement: giad085_Response_to_Reviewer_Comments_Revision_1 [file giad085_response_to_reviewer_comments_revision_1.pdf]

Author's Response To Reviewer Comments

Dear Editor and Reviewers,

Thank you for your previous reviews. We addressed all your feedback, and we detailed each change in the document submission_reviews_giga_science_2nd_round. Hope we met your expectations.
