# Supplementary material for: Computational prediction of human deep intronic variation
Source: Gigascience. 2023 Oct 25;12:giad085. doi: 10.1093/gigascience/giad085 (PMC10599398; doi:10.1093/gigascience/giad085)
Supplement: giad085_Reviewer_1_Report_Original_Submission — Jeanâ€′Madeleine de Sainte Agathe -- 3/20/2023 Reviewed [file giad085_reviewer_1_report_original_submission.pdf]

Reviewer Report

Title: Computational prediction of human deep intronic variation

Version: Original Submission **Date:** 3/20/2023

Reviewer name: Jeanâ€'Madeleine de Sainte Agathe

Reviewer Comments to Author:

This manuscript presents an important and very exhaustive benchmark concerning intronic variant splicing predictors. The focus on deep-intronic variants is highly appreciated as it addresses a very crucial challenge of today's genetics. The authors present the different tools in a very clear and pedagogical way. I should add that this manuscript is pleasant to read. The authors use the average precision score, allowing a refined comparison between tools. They give practical recommendations. They emphasize the use of SpliceAI and pangolin for intronic variants. For branchpoint regions, they recommend Pangolin and LabRanchoR.
It should be noted that this study is to my knowledge the first independent benchmark of Pangolin, CI-SpliceAI, ConSpliceML, AbSplice-DNA, SQUIRLS, BPHunter, LaBranchoR and SPiP together.
Overall, this study is important as it will be very helpful for the interpretation of intronic variants. I hence fully and strongly support its publication.
I have several comments that (I think) should be addressed before publication, especially the first point:
1) I admit that the curation of such large datasets is challenging, however, I failed to find some of the Table S6 variants in the referenced work. Please, could you kindly point me to the referenced variation for the following variants?
- The variant "1 hg38_156872925 C T NTRK1 ENST00000524377.1:c.851-708C>T pseudoexon_inclusion keegan_2022" is classified as 'affects_splicing'. However, I did not find it in Keegan 2022 (reference 20). In Keegan, the table S1 mentions NTRK1 variants but not c.851-708C>T. For these NTRK1 variants, keegan et al refers to another publication Geng et al 2018 (PMC6009080), where I can't find the ENST00000524377.1:c.851-708C>T variants neither.
- Same for "COL4A3 ENST00000396578.3:c.4462+443A>G 2:g.228173078A>G"
- Same for "ABCA4 ENST00000370225.3:c.1937+435C>G 1:g.94527698G>C"
- Same for "FECH ENST00000382873.3:c.332+668A>C 18:g.55239810T>G"
- Concerning "MYBPC3 ENST00000545968.1:c.1224-52G>A 11:g.47364865C>T" , I did not find it in pbarbosa as stated, but in another reference which, I think, should be mentioned in this manuscript: https://pubmed.ncbi.nlm.nih.gov/33657327/
- "BRCA2 ENST00000544455.1:c.8332-13T>G 13:g.32944526T>G" is classified as splicing neutral based on moles-fernÃ¡ndez_2021, but it has previously been shown to alter splicing (https://pubmed.ncbi.nlm.nih.gov/31343793/), please clarify.
If these variants were somehow erroneously included, the authors should reprocess their results with the corrected datasets.
2) Although it has been done before, the usage of gnomAD variants as a base of splicing-neutral variants is questionable. Indeed, it is theoretically possible that such variants truly alter splicing. For example,

genuine splicing alterations can result in mild inframe consequences on the gene products. Or splicing alterations can damage non-essential genes. I suggest that the authors:

-either select another gnomAD variants list located in disease-associated genes, where benign splicing alterations seem less plausible.

-or discuss this putative limitation in their results.

3) Table S8: "Variants above 0.05, the optimized SpliceAI threshold for non-canonical intronic splicing variation" Is that a recommendation of this work? Or was it found elsewhere? Please clarify.

More generally, this manuscript uses Average Precision scores, but the authors should explain to their non-statistician readers how it relates to the delta scores of each tool (Fig 3C). Indeed, any indication (or even recommendation, but not necessarily) concerning the use of cut-off values would be very appreciated by the geneticist community.

4) p.3 "If the model is run twice, once with the reference and once with the mutated sequence, it is possible to measure splice site alterations caused by genetic variants." This study makes only use of the delta scores, which have previously been shown to be misleading in some rare cases (PMID 36765386). The authors would be wise to mention this. For example, in Table S3, "ENST00000267622.4:c.5457+81T>A 14(hg19):g.92441435A>T" is predicted by SpliceAI DG=0.16, but as the reference prediction is already at 0.84, this 0.16 is the maximal delta score possible, yielding donor score = 1.

5) p.12 "Among the tools that predict across whole introns, SQUIRLS and SPiP are the only ones designed to provide some interpretation of the outcome." Concerning the nature of the mis-splicing event, I think the authors should mention SpliceVault, which has been specifically built for this task (pmid 36747048).

6) p.14: "SpliceAI and Pangolin […]. If usability is a concern and users do not have a large number of predictions to make, SpliceAI is preferred since the Broad Institute has made available a web app for the task" Now, the broad institute web app includes pangolin (at least for hg38 variants). Please, rephrase of delete this sentence.

7) Concerning complex delins, which are not annotated with the current version of SpliceAI, the authors should give recommendations. For example, the complex delins from tableS9 "hg19_chr7 5354081 GC AT" is correctly predicted by CI-SpliceAI and SpliceAI-visual, both tools allowing the annotation of complex delins with the SpliceAI model.

8) p.8 "Unfortunately, BPHunter only reported the variants predicted to disrupt the BP, rendering the Precision-Recall Curves (PR Curves) analysis impossible." I agree with the authors. However, I think it is sometimes assumed (wrongly?) that all variants unannotated by BPhunter have BPH_score=0. Maybe the authors could explicit this. For example, by saying that the lack of prediction cannot be safely equated with a negative prediction.

Methods

Are the methods appropriate to the aims of the study, are they well described, and are necessary controls included? Choose an item.

Conclusions

Are the conclusions adequately supported by the data shown? Choose an item.

Reporting Standards

Does the manuscript adhere to the journal's guidelines on [minimum standards of reporting?]() Choose an item.

Choose an item.

Statistics

Are you able to assess all statistics in the manuscript, including the appropriateness of statistical tests used? Choose an item.

Quality of Written English

Please indicate the quality of language in the manuscript: Choose an item.

Declaration of Competing Interests

Please complete a declaration of competing interests, considering the following questions:

- Have you in the past five years received reimbursements, fees, funding, or salary from an organisation that may in any way gain or lose financially from the publication of this manuscript, either now or in the future?
- Do you hold any stocks or shares in an organisation that may in any way gain or lose financially from the publication of this manuscript, either now or in the future?
- Do you hold or are you currently applying for any patents relating to the content of the manuscript?
- Have you received reimbursements, fees, funding, or salary from an organization that holds or has applied for patents relating to the content of the manuscript?
- Do you have any other financial competing interests?
- Do you have any non-financial competing interests in relation to this paper?

If you can answer no to all of the above, write 'I declare that I have no competing interests' below. If your reply is yes to any, please give details below.

I declare that I have no competing interests

I agree to the open peer review policy of the journal. I understand that my name will be included on my report to the authors and, if the manuscript is accepted for publication, my named report including any attachments I upload will be posted on the website along with the authors' responses. I agree for my report to be made available under an Open Access Creative Commons CC-BY license (http://creativecommons.org/licenses/by/4.0/). I understand that any comments which I do not wish to

be included in my named report can be included as confidential comments to the editors, which will not be published.

Choose an item.

To further support our reviewers, we have joined with Publons, where you can gain additional credit to further highlight your hard work (see: https://publons.com/journal/530/gigascience). On publication of this paper, your review will be automatically added to Publons, you can then choose whether or not to claim your Publons credit. I understand this statement.

Yes Choose an item.
