# Supplementary material for: Computational prediction of human deep intronic variation
Source: Gigascience. 2023 Oct 25;12:giad085. doi: 10.1093/gigascience/giad085 (PMC10599398; doi:10.1093/gigascience/giad085)
Supplement: giad085_Reviewer_2_Report_Original_Submission — Raphael Leman -- 4/14/2023 Reviewed [file giad085_reviewer_2_report_original_submission.pdf]

Reviewer Report

Title: Computational prediction of human deep intronic variation

Version: Original Submission **Date:** 4/14/2023

Reviewer name: Raphael Leman

Reviewer Comments to Author:

Summary:
In this work Barbosa et al., presented a benchmarking of several splicing predictors for human intronic variants. Overall, the results of this study shown that deep learning based tools such as SpliceAI outperformed the other splicing predictors to detect splicing disturbing variants and so pathogenic variants. The authors also detailed the performances of these tools on several subsets of data according to the collection origins of variants and according to the genomic localization of variants.
This work is one of the first large and independent studies about splicing prediction performances among intronic variants and in particular among deep intronic variants in a context of molecular diagnosis. This work also highlights the need to have reliable prediction tools for these variants and that the splicing impact of these variants are often underestimated.
However, I estimated that major points should to be solved before considering the article to publication.
Major points
1 The most important point is that authors shown results in the main text but in following paragraphs they claimed that these results were biased. In addition, the results, taking into account these biases, were only shown in supplementary data and the readers should make the correction themselves to get the "true" results. Indeed, the interpretation of biased results and "true" results changes drastically. The two main biases were: i) the use of ClinVar data already used for the training of CAPICE (see my following comment nÂ°2-), ii) the intronic tags of variants and the relative distance to the nearest splice site were wrong (see my following comment nÂ°5-). Consequently, the authors should remove these biased results and only show results after bias correction.
2 Importantly, several tools used ClinVar variants or published data to train and/or validate their models. Therefore, to perform a benchmark on true independent collection of variants, the authors should ensure the lack of overlapping between variants used for the tool development and this present study.
3 As authors shown by the comparison between the ClinVar classification (N = 54,117 variants) and impact on RNA from in vitro studies (N = 162 variants), there was discrepancies between this two information (N = 13/74 common variants, 18%). Consequently, using ClinVar classification to assay the performance of splicing prediction tools is not optimal.
To partially fix this point, I think further studying (ex: get minor allele frequency, availability of in vitro RNA studies, …) the intronic variants with positive splicing predictions from two or more tools with a ClinVar classification benign or likely benign and inversely, the intronic variants with negative splicing predictions from two or more tools with a ClinVar classification pathogenic or likely pathogenic could be interesting.

4 The authors used pre-computed databases for 19 tools, but the most of these databases do not include small insdels and so add artificially missing data in disfavor of the tool although the same tool could score these indels variants in de novo way.
5 The authors said that "We hypothesized that variability in transcript structures could be the reason [increase in performance in the deepest intronic bins]: despite these variants being assigned as occurring very deep within introns (> 500bp from the splice site of the canonical isoform) in the reference isoform, they may be exonic or near-splice site variants of other isoforms of the associated gene". To solve this transcript structure variability, firstly the authors could use weighted relative distance as following: |(|Pos_(nearest splice site)-Pos_variant |)-Intron_Size |â„(Intron_Size). Secondly, the ClinVar data contains the RefSeq transcript ID on which the variant was annotated (except for large duplications/deletions), so the authors should make the correspondence between these RefSeq transcript IDs and the transcripts used to perform splicing predictions.
6 With respect to the six categories of splice-altering variants, it is unclear how the authors considered cases in which variants alter physiological splice motives (e.g., natural consensus sequences 3'SS/5'SS, branch point, or ESR) but, instead of exon skipping, the spliceosome recruits another distant splice site that is partially or not affected by the variant.
7 In the table 1 listing the tools considered for this study, please explicit for each tool on which collections of data (ClinVar or splicing altering variants) and for which genomic regions the benchmark was done. This information will facilitate the reading of the article.
8 Accordingly to my comment nÂ°3-, all spliceogenic variants are not necessary pathogenic. The mutant allele could produce aberrant transcripts without a frame-shift and without impact the functional domains of the protein. In addition, the transcription could also lead to a mix between aberrant transcript and full-length transcript. As a result, the main goal of splicing prediction tools is to detect splicing altering varaints. Considering variants with positive splicing prediction as pathogenic is a dangerous shortcut and only an in vitro RNA study could confirm the pathogenicity of a variant. The discussion section should be update in this sense.
9 The authors claimed that: "The models [SQUIRLS and SPiP] were frequently able to correctly identify the type of splicing alteration, yet they still fail to propose higher-order mechanistic hypotheses for such predictions.". I think that the authors over-interpreted the results (see my comment nÂ° 21-).
10 The authors recommended prioritizing intronic variants using CAPICE, It is still true once the bias was corrected (see my comment nÂ°1-).
Minor points
11 In the introduction the authors could clearly define the canonical splice site regions (AG/GT dinucleotides in 3'SS: -1/-2 and 5'SS: +1/+2) to make the difference with the consensus splice sites commonly define as: 3'SS: -12 (or -18)/+2 and 5'SS: -3/+6.
12 In the introduction, please also add that splice site activation could be also due to disruption of silencer motif.
13 In the ref [17], the authors did not say that the enrichment of splicing related variants within splice site regions was linked to exons and splice sites sequencing. They proved that whole genome sequencing increased the diagnostic rate of rare genetic disease, actually they did not focus on splicing variants. This enrichment was more probably induced by the fact that geneticists mainly studied variants with positive splicing predictions.

14 In the paragraph 'The prediction tools studied are diverse in methodology and objectives', please add that most of prediction tools target consensus splice sites (ex: MES, SSF, SPiCE, HSF, Adaboost, …).
15 In the paragraph 'The prediction tools studied are diverse in methodology and objectives', the authors claimed that 'sequence-based deep learning models such as SpliceAI, which do not accept genetic variants as input.' but it is wrong as SpliceAI could accept VCF file as input.
16 In the paragraph 'Pathogenic splicing-affecting variants are captured well by deep learning based methods', this is further explained in the section method, but I think a sentence explaining that the 243 variants were from 81 variants described in ref [19] and 162 variants from a new collection will clarify the reading of article
17 In the paragraph 'Pathogenic splicing-affecting variants are captured well by deep learning based methods', among the 13 variants incorrectly classified, please detailed how many variants were classified as benign and VUS.
18 Due to the blue gradient, the Fig 1C is hard to analyze.
19 In the paragraph 'Branchpoint-associated variants', the variant rapported in the ref [79] were studied within tumoral context and so the observed impact could not be the same in healthy tissue.
20 In the paragraph 'Exonic-like variants', the authors changed the parameters of SpliceAI predictions, from the original prarameters used for the precomputed scores, to take into account variants located deep inside the pseudoexon. Please ensure whether other prediction tools have also user-defined optimizable parameters to take into account these variants.
21 In the paragraph 'Assessing interpretability', the authors observed that non-informative SPiP annotations presented a high score level. This could be explained by the fact of the tool report a positive prediction without annotation only because the model score was high without a relation to a particular splicing mechanism.
22 In the paragraph 'Assessing interpretability', the authors could compare the SpliceAI annotations regarding the abolition/creation of splice sites and their relative positions to the variants to the observed effect on RNA.
23 In the paragraph 'Predicting splicing changes across tissues', by my count the analysis of AbSplice-DNA predictions was done on 89 variants (154 - 65 = 89), if true please indicate clearly in the text.
24 In the method section, paragraph "ClinVar", the 13 variants with discordance between the classification and the observed splicing impact, how many did they have confidence stars.
25 In the method section, paragraph "Disease-causing intronic variants affecting RNA splicing", the authors filtered out variants within the 10 pb around the nearest splice site, please explicit why.
26 In the method section, paragraph "Disease-causing intronic variants affecting RNA splicing", the authors used gnomAD variants as control set, however their threshold of variant frequency is too low (1%). Indeed, some pathogenic variants involved in recessive genetic disorders have a high frequency in population. A threshold of 5% is more appropriate.
27 In the method section, paragraph "Variants that affect RNA splicing", the authors should describe how they considered variants leading to multiple aberrant transcripts and variants with partial effect (i.e., allele mutant still producing full length transcript).
28 In the method section, paragraph "Variants that affect RNA splicing", regarding the six categories defined by the authors:
How the indels variants were annotated if they overlapped between several categories.

The new splice donor/acceptor categories included only variants creating new AG/GT or variants occurring within the consensus sequences of cryptic splice sites.
Among the category Donor-downstream, please make the distinction between variants located between [+3; +6] bp (i.e. consensus sequence) and variant beyond +6 bp.
The exonic-like variants could be variants that did not impact ESRs motives (see my comment nÂ°6-).
29 In the method section, paragraph "Variants that affect RNA splicing", the authors select for the control datasets, variants generating the CAGGT and GGTAAG motives. However, this approach lead to an over-enrichment of false positives. Moreover, it could be also interesting if among the variants creating new splice sites or pseudoexons to identify the presence of GC donor motif or U12-minor spliceosome motif (AT/AC) and how the different splicing tools can detect them.
30 In Fig S3C, scale the gnomAD population frequency in -logâ,â,€(P) to make the figure more readable.
31 I saw several times double spaces in the text please correct them. English is not my native language so I am not the best judge, but some sentences seem syntactically incorrect (ex: "The splicing tools with the smallest and largest performance drop between the splice site bin ("1-2") and the "11-40" bin were Pangolin and TraP, with weighted F1 scores decreasing by 0.334 and 0.793, respectively"). Please have the article proofread by someone who is fluent in English.

Methods

Are the methods appropriate to the aims of the study, are they well described, and are necessary controls included? Choose an item.

Conclusions

Are the conclusions adequately supported by the data shown? Choose an item.

Reporting Standards

Does the manuscript adhere to the journal’s guidelines on minimum standards of reporting? Choose an item.

Choose an item.

Statistics

Are you able to assess all statistics in the manuscript, including the appropriateness of statistical tests used? Choose an item.

Quality of Written English

Please indicate the quality of language in the manuscript: Choose an item.

Declaration of Competing Interests

Please complete a declaration of competing interests, considering the following questions:

- Have you in the past five years received reimbursements, fees, funding, or salary from an organisation that may in any way gain or lose financially from the publication of this manuscript, either now or in the future?
- Do you hold any stocks or shares in an organisation that may in any way gain or lose financially from the publication of this manuscript, either now or in the future?
- Do you hold or are you currently applying for any patents relating to the content of the manuscript?
- Have you received reimbursements, fees, funding, or salary from an organization that holds or has applied for patents relating to the content of the manuscript?
- Do you have any other financial competing interests?
- Do you have any non-financial competing interests in relation to this paper?

If you can answer no to all of the above, write 'I declare that I have no competing interests' below. If your reply is yes to any, please give details below.

I declare that I have no competing interests

I agree to the open peer review policy of the journal. I understand that my name will be included on my report to the authors and, if the manuscript is accepted for publication, my named report including any attachments I upload will be posted on the website along with the authors' responses. I agree for my report to be made available under an Open Access Creative Commons CC-BY license (http://creativecommons.org/licenses/by/4.0/). I understand that any comments which I do not wish to be included in my named report can be included as confidential comments to the editors, which will not be published.

Choose an item.

To further support our reviewers, we have joined with Publons, where you can gain additional credit to further highlight your hard work (see: https://publons.com/journal/530/gigascience). On publication of this paper, your review will be automatically added to Publons, you can then choose whether or not to claim your Publons credit. I understand this statement.

Yes Choose an item.
