# Supplementary material for: Computational prediction of human deep intronic variation
Source: Gigascience. 2023 Oct 25;12:giad085. doi: 10.1093/gigascience/giad085 (PMC10599398; doi:10.1093/gigascience/giad085)
Supplement: giad085_Reviewer_2_Report_Revision_1 — Raphael Leman -- 6/22/2023 Reviewed [file giad085_reviewer_2_report_revision_1.pdf]

Reviewer Report

Title: Computational prediction of human deep intronic variation

Version: Revision 1 **Date:** 6/22/2023

Reviewer name: Raphael Leman

Reviewer Comments to Author:

I thank the authors for this review. Overall, the authors successfully addressed my comments and the modified manuscript is more precise and clearer. I hence strongly support its publication. I have few minor comments:

- According to my comment #1 of the first review, the authors corrected the set of ClinVar data to remove the training variants of CAPICE tool. Consequently, the performance of CAPICE was largely reduced with a top-ranking of 8 against 4 in the initial version. Interestingly, the new performance of CAPICE was in line with other tools that used ClinVar data in their development (ex: SQUIRLS, 7th-top ranking or CADD-splice, 9th-top ranking). I agree with the discussion of the authors, the use of ClinVar data did not seem to induce a major bias as the best tools of this study were not the tools using ClinVar data in their training.
- About my comment #6 of the first review, I can understand that my explanation was not enough clear. I think is better if I take some examples. In BRCA2 gene (NM_000059) the variant c.682-2A>C led to, as expected for a canonical variant, an exon skipping but the major aberrant splicing event was the use of an acceptor splice site at 73 nt downstream of the natural splice site (http://dx.doi.org/10.1016/j.jmoldx.2014.01.005). In NF1 gene (NM_000267) the variant c.204+1G>A led to the use of a donor splice site at 104 nt upstream of the natural splice site (http://dx.doi.org/10.1002/humu.20826). In both two case, the new sites do not seem to be impact by the variant and their use is not predicting by the tools, ex: SpliceAI: c.682-2A>C AL: 1.00 (2); AG: 0.11 (13) and c.204+1G>A DL: 1.00 (-1); DG: 0.02 (-46). It would be reasonable to think that the authors have similar variants in their collection of variants.

Methods

Are the methods appropriate to the aims of the study, are they well described, and are necessary controls included? Choose an item.

Conclusions

Are the conclusions adequately supported by the data shown? Choose an item.

Reporting Standards

Does the manuscript adhere to the journal's guidelines on [minimum standards of reporting?]() Choose an item.

Choose an item.

Statistics

Are you able to assess all statistics in the manuscript, including the appropriateness of statistical tests used? Choose an item.

Quality of Written English

Please indicate the quality of language in the manuscript: Choose an item.

Declaration of Competing Interests

Please complete a declaration of competing interests, considering the following questions:

- Have you in the past five years received reimbursements, fees, funding, or salary from an organisation that may in any way gain or lose financially from the publication of this manuscript, either now or in the future?
- Do you hold any stocks or shares in an organisation that may in any way gain or lose financially from the publication of this manuscript, either now or in the future?
- Do you hold or are you currently applying for any patents relating to the content of the manuscript?
- Have you received reimbursements, fees, funding, or salary from an organization that holds or has applied for patents relating to the content of the manuscript?
- Do you have any other financial competing interests?
- Do you have any non-financial competing interests in relation to this paper?

If you can answer no to all of the above, write 'I declare that I have no competing interests' below. If your reply is yes to any, please give details below.

I declare that I have no competing interests

I agree to the open peer review policy of the journal. I understand that my name will be included on my report to the authors and, if the manuscript is accepted for publication, my named report including any attachments I upload will be posted on the website along with the authors' responses. I agree for my report to be made available under an Open Access Creative Commons CC-BY license (http://creativecommons.org/licenses/by/4.0/). I understand that any comments which I do not wish to be included in my named report can be included as confidential comments to the editors, which will not be published.

Choose an item.

To further support our reviewers, we have joined with Publons, where you can gain additional credit to further highlight your hard work (see: https://publons.com/journal/530/gigascience). On publication of this paper, your review will be automatically added to Publons, you can then choose whether or not to claim your Publons credit. I understand this statement.

Yes Choose an item.
